# A readily accessible CH anion transfer reagent for the preparation of a molybdenum methylidyne complex

**DOI:** 10.1039/d5sc07469j

**Published:** 2026-02-19

**Authors:** Rajesh Mukkera, Nghia Le, Chandler I. Woo, Charles Edwin Webster, Sidney E. Creutz

**Affiliations:** a Department of Chemistry, Mississippi State University Mississippi State MS 39762 USA screutz@chemistry.msstate.edu ewebster@chemistry.msstate.edu

## Abstract

A route to a readily accessible source of an anionic methylidyne group (CH^−^) was developed *via* lithium–halogen exchange of 11-iodo-9,10-dihydro-9,10-methanoanthracene (MA-I). Upon reaction of this alkyllithium precursor with the complex (TMS-TREN)MoCl as a test platform, the methylidyne complex (TMS-TREN)Mo≡CH is quantitatively and rapidly delivered with concomitant loss of anthracene. The kinetics and mechanisms of this reaction are investigated experimentally and computationally and suggest the intermediacy of a metal cycloalkyl complex that releases anthracene through a stepwise pathway *via* a radical intermediate; the rate of this bond cleavage reaction is more than six orders of magnitude faster than the previously reported route to this complex *via* ethylene loss from a metal cyclopropyl complex.

## Abstract 2

A new reagent for delivering a “CH” anion is developed and demonstrated in the preparation of a molybdenum methylidyne complex. Computational and kinetic studies suggest a stepwise pathway *via* a radical intermediate.

## Graphical abstract

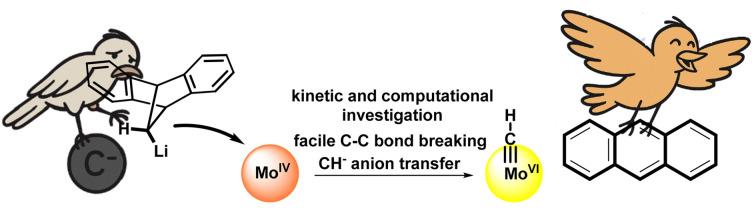

## Introduction

Terminal methylidyne complexes (M≡CH) are of considerable scientific interest due to their potential relevance to technologically important reactions including small-molecule activation (*e.g.*, CO_2_ reduction and functionalization) and metathesis, and as potential surface species in heterogeneous catalytic reactions including methane activation and the Fischer–Tropsch process.^1–4^ They are also of fundamental interest in elucidating the electronic structure and reactivity of metal-carbon multiple bonds. To date, terminal methylidyne complexes have been characterized primarily on early 2nd- and 3rd-row transition metals, and crystallographically characterized examples are reported only for Mo, Nb, and W.^5–8^ This suggests an ongoing need for new methodologies to access these species.

**Fig. 1:**
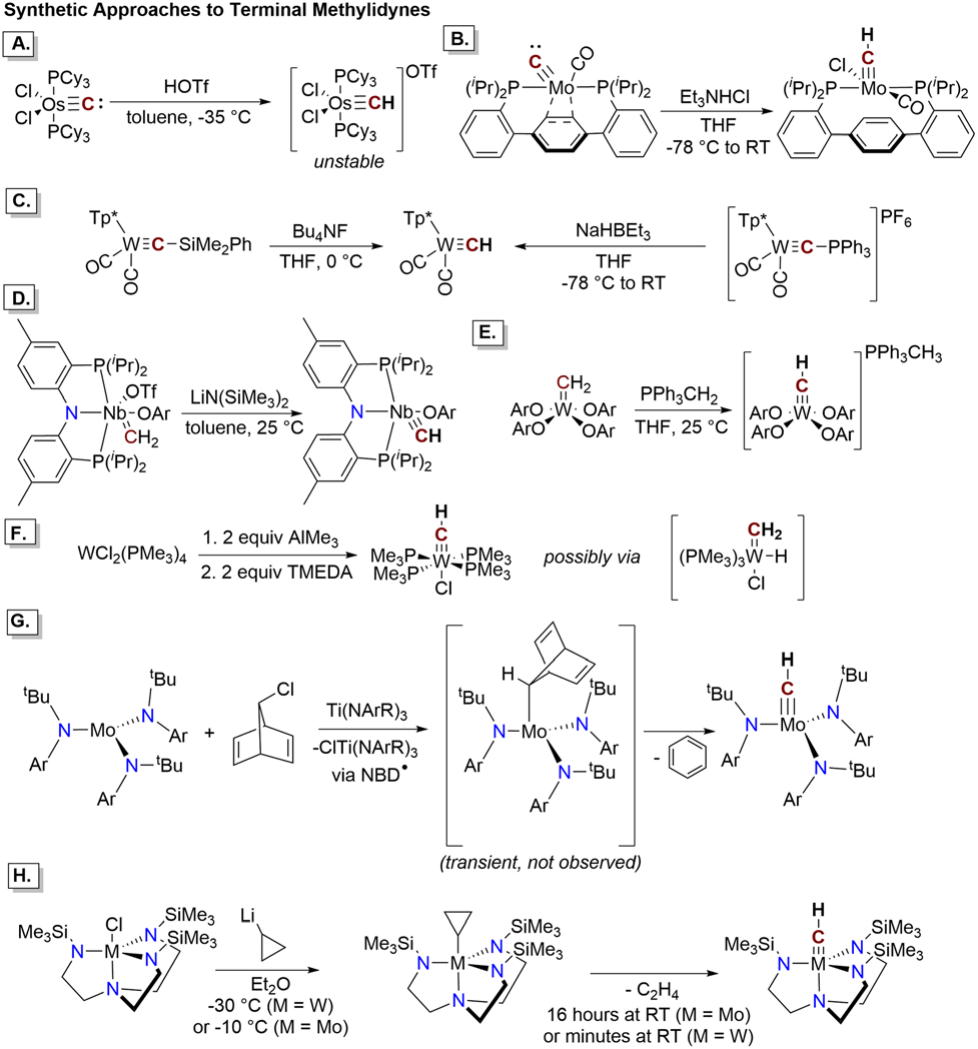
Previous methods to access terminal transition metal methylidyne complexes. Corresponding citations are given in the text. (A and B) Protonation of a terminal carbido. (C) Two methods to access a tungsten methylidyne by functional group exchange on a heterocarbyne. Tp* = tris(3,5-dimethyl-1-pyrazolyl)borate. (D and E) Deprotonation of a terminal methylidene. Ar = 2,6-diisopropylphenyl. (F) Formal H_2_ extrusion from a transient tungsten methyl complex *via* a methylidene hydride. (G) Loss of benzene from a norbornadienyl complex. The norbornadienyl complex is formed by reaction of the molybdenum(iii) trisanilide with norbornadiene radical generated *in situ*. Ar = 3,5-dimethylphenyl. (H) Loss of ethylene from a molybdenum or tungsten cyclopropyl complex.

Examples of prior strategies to access terminal methylidyne ligands are shown in Fig. 1. These commonly include protonation of a terminal carbido ligand (Fig. 1A and B), itself often generated by oxide or sulfide abstraction from a CO or CS ligand.^6,9–11^ Additionally, several methods for the exchange of a heteroatom substituent on a heterocarbyne species (Fig. 1C) have been explored.^12–14^ On the other hand, deprotonation of a methylidene ligand to provide a methylidyne (Fig. 1D and E) has been successfully applied to tungsten and niobium complexes.^5,8,15^ Formal extrusion of H_2_ from a metal methyl complex is a facile route to several examples of tungsten methylidynes (Fig. 1F).^16–18^ Finally, loss of a stable olefin (ethylene or benzene) from a metal cycloalkyl complex has been applied successfully to molybdenum and tungsten complexes (Fig. 1G and H).^18–20^

Dimetallic µ_2_–CH species are also relatively rare and have often been produced by similar routes, including formal loss of H_2_ from a methyl ligand or hydride loss (*via* abstraction or α-hydride elimination) from a µ_2_-CH_2_ group; studies on dimetallic systems have also revealed novel synthetic mechanisms including heterobimetallic carbido-hydrido coupling.^4,21–24^

**Fig. 2:**
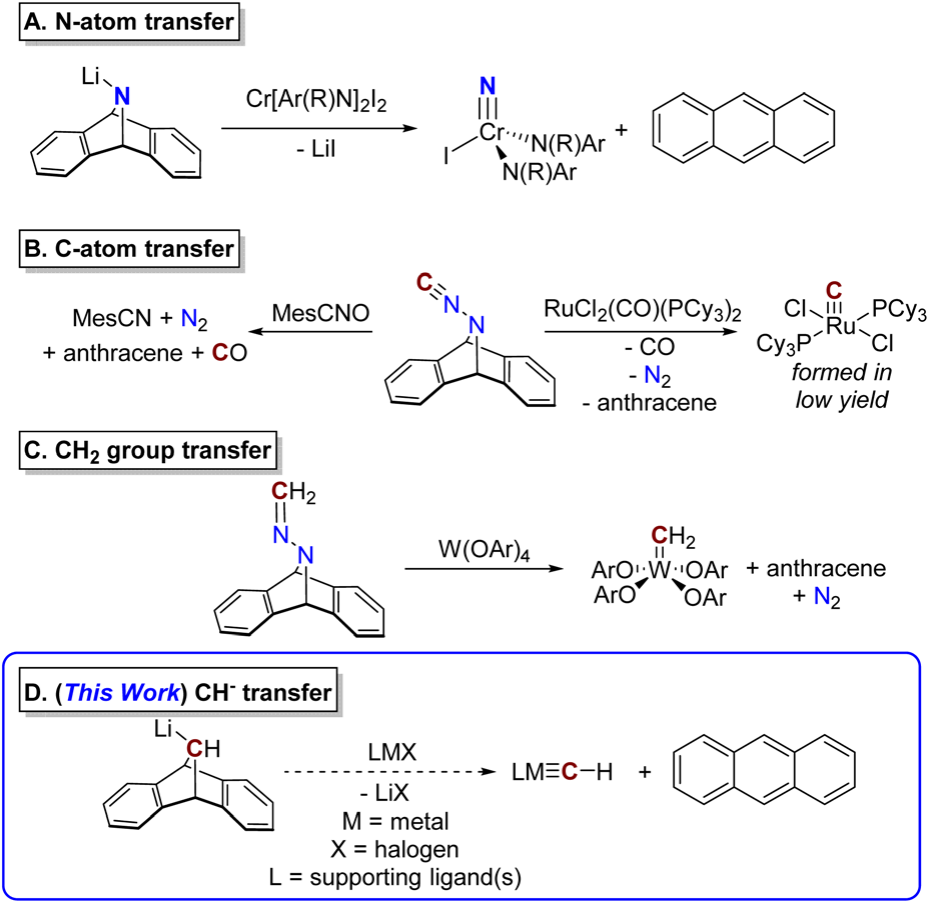
Group transfer to transition metals using “anthracene releasing” reagents. Corresponding references are given in the text. (A) N-atom transfer to a chromium complex. R = –C(CD_3_)CH_3_, Ar = 2-fluoro-5-methylphenyl. (B) C-atom transfer with concomitant loss of N_2_. (C) Methylene transfer with loss of N_2_. Ar = 2,6-diisopropylphenyl. (D) Transfer of a methylidyne group with loss of anthracene as reported in this work.

One very successful strategy developed over the last several decades to install metal–ligand multiply bonded groups has been atom- or group-transfer from anthracene-releasing reagents, including 7-azadibenzonorbornadiene-derived scaffolds (Fig. 2). In addition to the NH-bridged dibenzoazabicycloheptadiene species, which is used to prepare metal nitrides (after lithiation, Fig. 2A),^25^ derivatives with bridging –N_2_C and –N_2_CH_2_ groups have been developed, envisioned as sources of a C atom and a methylidene group, respectively, with concomitant loss of N_2_ (Fig. 2B and C).^8,26^

## Results and discussion

An analogous anthracene-releasing reagent capable of directly delivering a methylidyne (CH) moiety has not yet been reported in the literature, although preliminary efforts in this area have been described in a thesis.^27^ We envisioned that this gap could be filled through the development of a reagent that would act as a source of the organolithium species MA-Li or the analogous Grignard reagent, MA-MgX (Fig. 2D; MA = 9,10-dihydro-9,10-methanoanthracene, see below). This species could itself be generated by lithium–halogen exchange of a stable halide precursor, MA-X. The carbanion synthon MA-Li or MA-MgX could formally deliver the “CH^−^” anion to a metal complex with loss of anthracene, as generally illustrated in Fig. 2D. As a source of an anionic group, this reagent would act as an X-type ligand and would be well-suited to reactivity with metal complexes bearing exchangeable X-type ligands.

**Scheme 1:**
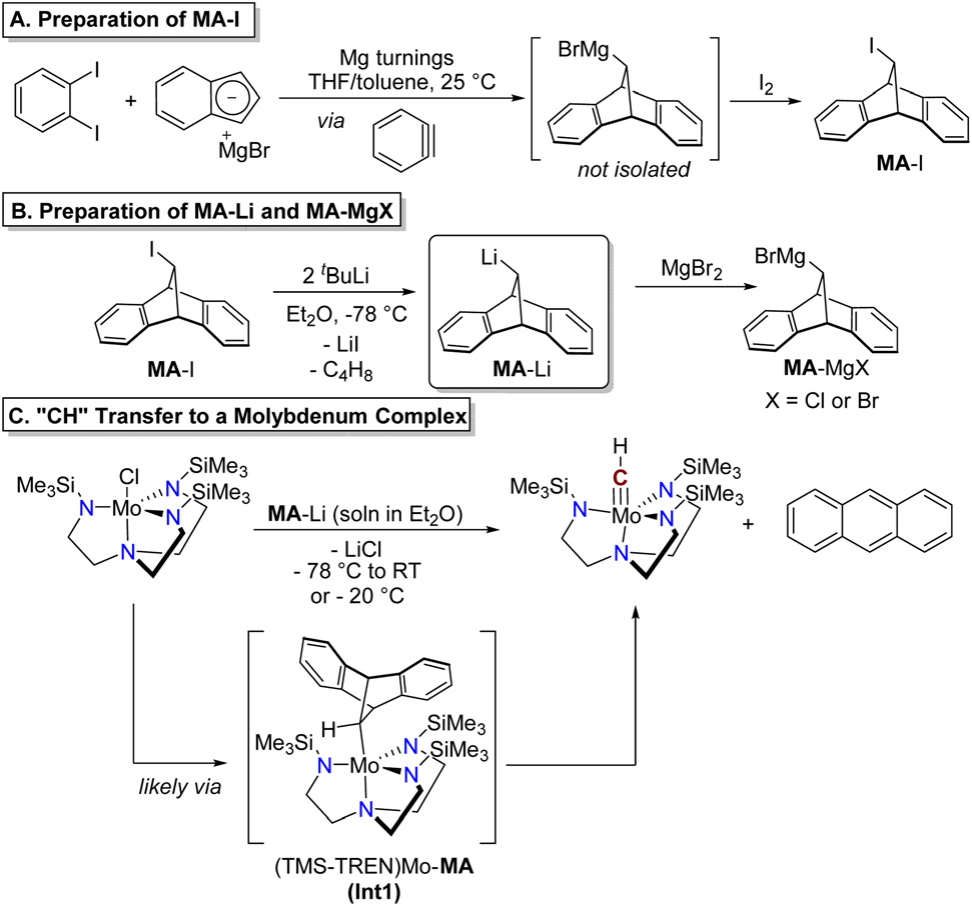
Preparation of MA-I (A), metalation to give MA-Li or MA-MgBr (B), and reaction with (TMS-TREN)MoCl to demonstrate methylidyne transfer (C).

Construction of the functionalized 9,10-dihydro-9,10-methanoanthracene (MA) scaffold was achieved through a cycloaddition reaction between benzyne and indenylmagnesium bromide (Scheme 1A). The benzyne intermediate was generated *in situ* from 1,2-diiodobenzene by stirring over magnesium turnings at room temperature in a mixture of THF and toluene. This cycloaddition reactivity was previously studied by Huebner and Donohue, who observed the formation of up to 22% yield of MA-H after hydrolysis of a similar reaction between indenylmagnesium bromide, *o*-bromofluorobenzene, and magnesium turnings, presumably initially forming the alkyl Grignard intermediate MA-MgBr prior to quenching.^28,29^ In order to set the stage for further reactivity at this site, we quenched this intermediate with I_2_ to provide the product MA-I. This reagent is indefinitely stable in air at room temperature and was readily isolated in pure form by recrystallization from acetonitrile and diethyl ether. Although the isolated yield of MA-I was modest (29%), the reaction could be successfully carried out on a multigram scale (see SI). The reaction and product isolation are operationally simple and, including the preparation of indenylmagnesium bromide, synthesis of MA-I requires only two steps from commercially available reagents.

The alkyllithium reagent MA-Li was prepared as a solution in Et_2_O by lithium–halogen exchange with two equivalents of ^*t*^BuLi (Scheme 1B) at −78 °C. This species degrades in Et_2_O solution at room temperature under air-free conditions, giving protonated MA-H as the only product identifiable by NMR; in solution at −20 °C only 10% degradation is observed after four hours (Fig. S9). No anthracene was detected from the degradation of MA-Li in solution in the absence of an added transition metal complex. Alternatively, the alkyl Grignard reagent MA-MgBr could be prepared *in situ* by treating MA-Li with anhydrous magnesium bromide (Scheme 1B). The Grignard reagent is more stable in Et_2_O solution, with minimal degradation observed after storage at room temperature for ten days (Fig. S8).

We identified the Mo(iv) complex [(Me_3_SiNCH_2_CH_2_)_3_N]MoCl (abbreviated as (TMS-TREN)MoCl) as a test platform to determine the feasibility of anthracene release and “CH” transfer from MA-Li, hypothesized to occur *via* an intermediate alkyl complex as shown in Scheme 1C, (TMS-TREN)Mo-MA. Treatment of a solution of (TMS-TREN)MoCl with a solution of MA-Li in Et_2_O at −78 °C resulted in a rapid color change from orange to brown; after warming of the solution to room temperature and concentration *in vacuo*, NMR analysis of the crude residue showed the presence of the known diamagnetic molybdenum(vi) methylidyne complex (TMS-TREN)Mo≡CH and anthracene in a 1 : 1 ratio (Scheme 1C). Some protonated MA-H was also detected; no other products could be identified in the ^1^H NMR spectrum (Fig. S6).

**Fig. 3:**
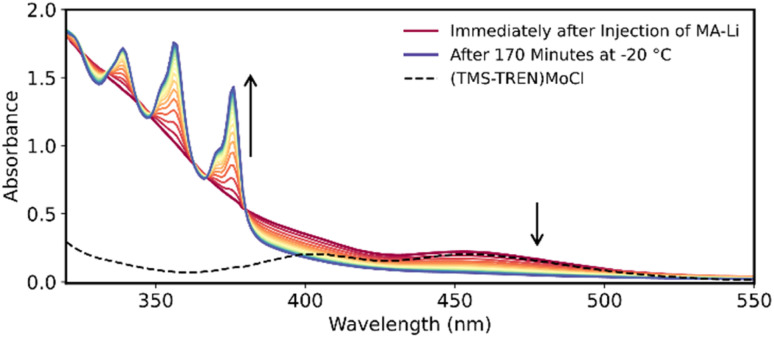
UV-vis spectra of the reaction between (TMS-TREN)MoCl (0.2 mM in Et_2_O) and MA-Li (0.4 mM in Et_2_O) at −20 °C. Dashed black line shows the spectrum of (TMS-TREN)MoCl prior to injection of MA-Li; solid-colored spectra show reaction after injection at 10-minute intervals (progressing from red to blue). Arrows show direction of change of the adjacent peaks.

The apparently rapid formation of (TMS-TREN)Mo≡CH is noteworthy because it contrasts with the slow (16 hours at room temperature) formation of (TMS-TREN)Mo≡CH by ethylene release from a related molybdenum(iv) cyclopropyl complex (Fig. 1H), suggesting that cleavage of MA in (TMS-TREN)Mo-MA to release anthracene is significantly more facile. In order to probe the kinetics of this process in more detail, we monitored the reaction by low-temperature UV-vis spectroscopy. Fig. 3 shows the spectra of a reaction between 0.2 mM (TMS-TREN)MoCl and 0.4 mM MA-Li at −20 °C in Et_2_O; spectra are shown at ten minute intervals. The starting molybdenum(iv) complex shows absorbance features at 405 and 460 nm, attributed to d*–*d transitions (Table S8); the d^0^ methylidyne product (TMS-TREN)Mo≡CH is nearly colorless and does not exhibit significant absorbance at wavelengths above 350 nm. The prepared MA-Li reagent solution exhibits a mostly featureless sloping absorption spectrum below approximately 450 nm (Fig. S9), which accounts for the immediate increase in absorbance in the UV region upon initial injection (Fig. 3, dark red spectrum).

**Fig. 4:**
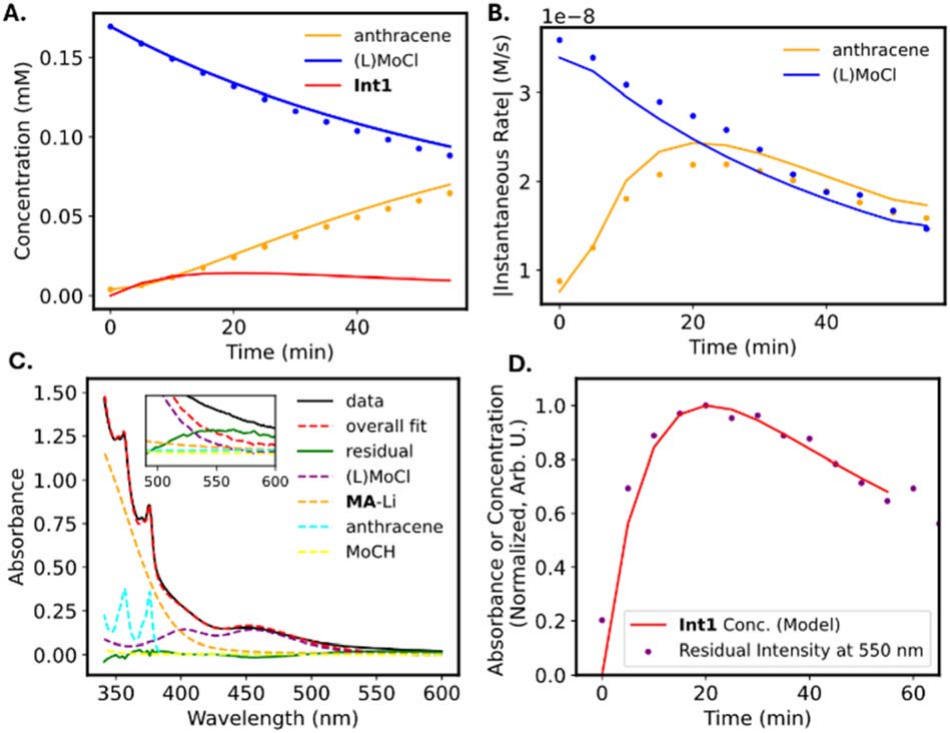
Kinetic analysis of methylidyne transfer based on UV-vis spectroscopy. Analysis corresponds to the spectra shown in Fig. 3 (A) Concentrations of anthracene and (TMS-TREN)MoCl (L = TMS-TREN) over the first 60 minutes of reaction. Dots represent measured concentrations determined from analysis of the UV-vis spectra; lines represented calculated concentrations based on the kinetic model described in the text. The calculated concentration of the proposed intermediate Int1 is also shown. (B) Absolute values of the gradients of the measured (dots) and calculated (line) concentrations of anthracene and (TMS-TREN)MoCl. (C) Decomposed spectrum of the reaction mixture after 15 minutes of reaction, showing residual peak at approximately 550 nm. Dashed lines represent fitted component spectra for known species. (D) Comparison of the (normalized) intensity of the residual peak shown in part C with the calculated (normalized) concentration of Int1 based on the kinetic model.

The progress of the reaction is evidenced by the growth of the sharp absorbance features associated with anthracene (345, 365, and 378 nm). An apparent isosbestic point is observed at 380 nm, along with several higher-energy crossing points that deviate slightly from isosbesticity. Decomposition of the spectra at each time point into contributions from the reactants and products (see discussion in SI) allowed us to extract the approximate concentrations of (TMS-TREN)MoCl and anthracene over the course of the reaction (Fig. 4A). Inspection of the resulting data shows that there is an apparent lag in the formation of anthracene relative to the consumption of (TMS-TREN)MoCl; this effect is also clear in a plot of the rate of production/consumption of the starting material and product (Fig. 4B). This is consistent with a two-step kinetic model (eqn (1) and (2)) with an initial second-order reaction between (TMS-TREN)MoCl and MA-Li to give an intermediate, Int1, which decays in a first order reaction to produce anthracene; both steps are assumed to be essentially irreversible.1
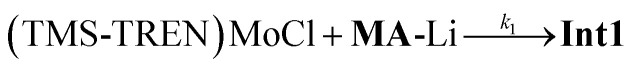
2
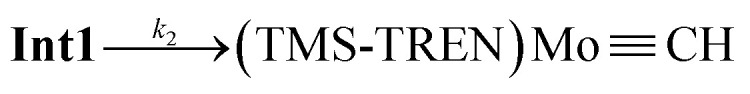


A small concentration of Int1 would be expected to build up early in the reaction. Fitting of this model to our data provides values of 29 ± 2 M^−1^ s^−1^ for *k*_1_ and 0.10 ± 0.01 s^−1^ for *k*_2_ under these conditions (−20 °C); similar values have been obtained from experiments with different starting concentrations of (TMS-TREN)MoCl and MA-Li (see SI, Table S1).

We hypothesized that the identity of Int1 could be the molybdenum alkyl complex shown in Scheme 1. Previously characterized examples of Mo(iv) cycloalkyl complexes on this ligand platform exhibit d*–*d absorbance features near 540–560 nm.^19^ Close examination of our data shows a weak residual peak near 550 nm whose intensity cannot be attributed to any of the other known species in solution (Fig. 4C); the intensity of this feature reaches a maximum after approximately 20 minutes and follows a trajectory close to that predicted by the kinetic model for Int1 (Fig. 4D). Computational analysis of Int1 in the triplet state confirms that this species is predicted to have a *d*–*d* transition absorbing in the visible region (see Fig. S24 and accompanying discussion), although other possible intermediates are also predicted to absorb in this region (Fig. S26–S28); therefore, we cannot exclude the possibility that the observed absorbance corresponds to a different intermediate or to a mixture of species. The instability of this species and its presence at only relatively low concentrations has so far prevented further detailed experimental study. Regardless of the precise assigment, the decay rate of the intermediate to provide (TMS-TREN)Mo≡CH is more than six orders of magnitude faster than that of the (TMS-TREN)Mo(cyclopropyl) complex (which decomposes according to a first-order rate constant of 1.4× 10^−8^ s^−1^ at −20 °C, extrapolated from reported higher temperature data),^19^ validating the utility of MA-Li as kinetically competent vehicle for the formal delivery of the methylidyne anion.

Despite the utility of anthracene-releasing reagents related to MA (Fig. 2), limited atomistic information about the mechanism of bond cleavage and anthracene loss during group transfer to transition metal complexes has been reported based on either experimental or computational studies.^26,30^ In particular, bond cleavage in non-strained cyclic systems is typically associated with a high kinetic barrier. Therefore, the observed rapid process involving the breaking of two C–C bonds is of considerable mechanistic interest.

**Scheme 2:**
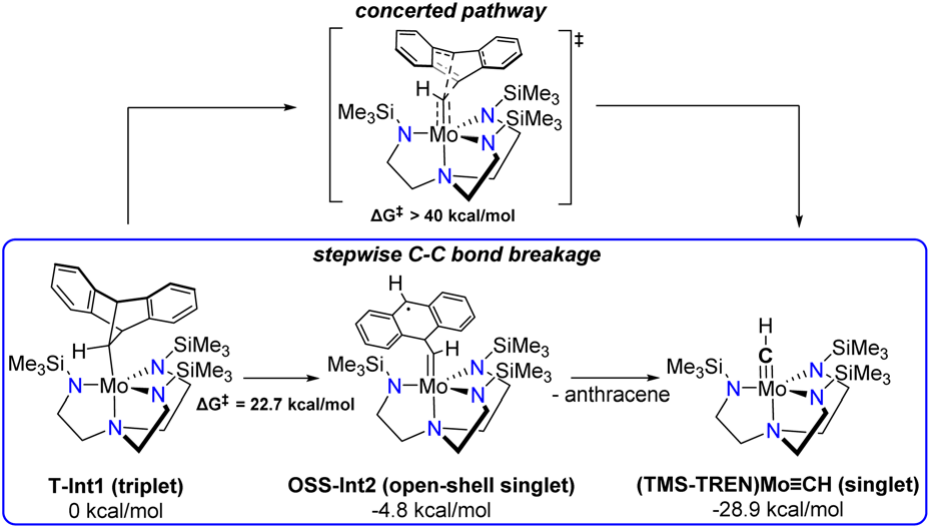
Concerted *vs.* stepwise C–C bond cleavage pathways considered computationally, not including Li^+^ coordination.

The proposed intermediate Int1 can exist in either singlet or triplet states. DFT computations show that the triplet T-Int1 is more stable than the singlet S-Int1 by 10.1 kcal mol^−1^, consistent with the triplet ground states observed for previously characterized (TMS-TREN)Mo(iv)-alkyl complexes.^19^ The calculated free energy diagram for the subsequent bond cleavage steps is shown in Fig. S19. The first C–C bond cleavage in the stepwise mechanism (Scheme 2) is most facile on the triplet surface, with the transition state T-TS1 having a calculated free energy of activation (Δ*G*^‡^) of 22.7 kcal mol^−1^. The resulting intermediate, Int2 (Scheme 2), is calculated to be quite stable, lying slightly lower in energy than the starting complex, despite bearing significant radical character on the nascent anthracene moiety. This stability can likely be attributed at least in part to delocalization of the radical character over the aromatic rings, as illustrated in the spin–density plots of this species (Fig. S19). The open-shell singlet state of the biradical (OSS-Int2) was found to be slightly lower in energy (by 2.1 kcal mol^−1^) than the triplet state (T-Int2).

After the first bond cleavage, the computations predict that Int2 undergoes a second C–C bond cleavage, ultimately producing the (TMS-TREN)Mo≡CH product as a closed-shell singlet. During this transformation, we located a crossing between the closed-shell singlet and open-shell surfaces with the minimum energy crossing point (MECP) at Δ*E* = 13.0 kcal mol^−1^ (Fig. S22). Spin–density plots during the elongation of the second C–C bond reveal delocalization of electron density between the Mo center and the anthracene system, which becomes increasingly localized at the Mo center as the bond distance increases (Fig. S22).

In contrast to the stepwise pathway, a concerted pathway for anthracene dissociation could not be located but is estimated to have a barrier higher than 40 kcal mol^−1^ on the constrained surfaces, effectively ruling it out. A number of other possibilities, including pathways involving C–C bond cleavage leading to a metallacycloalkene intermediate as suggested by Schrock *et al.*,^19^ α-hydride elimination, or proton transfer to the ligand were considered but no lower–barrier pathways to the observed products were found.

From the free energy diagram (Fig. S20), the rate-determining step corresponds to the cleavage of the first C–C bond. DFT computations predict a Δ*G*^‡^ of 22.7 kcal mol^−1^, higher than the transition state energy suggested by the experimental rate constant (∼16 kcal mol^−1^). We have explored the possibility that this discrepancy could be related to interactions with other species in the reaction mixture not yet explicitly considered in our computations, such as halide ion or lithium cation. Our computations showed some evidence for stabilization of the transition state for the initial C–C bond cleavage by interaction of lithium cation with the aromatic rings of the methanoanthracene-derived ligand (Fig. S21). This builds conceptually off of prior computational work by Perez and Domingo who showed that Li^+^ coordination lowers the barriers involved in C–C bond formation reactions on related substrates.^31^ Our calculations show that Li^+^ coordination can lower the Δ*G*^‡^ for the initial C–C bond cleavage to Δ*G*^‡^ = 18.4 kcal mol^−1^ (Fig. S21). In addition, the Li^+^ ion stabilizes the singlet relative to the triplet states after the initial bond cleavage. This stabilization arises from delocalization of the additional positive charge onto the Mo center, which lowers the energy of the occupied orbitals and increases ligand field splitting, thereby favoring the singlet state. Determining the precise energetics of this step in the context of the real reaction mixture is complicated by Li^+^ solvation equilibria and interaction with other species (*e.g.* halide ions). However, these results suggest a potentially significant role for Li^+^ in facilitating C–C bond cleavage and allowing the complex to cross to the singlet manifold earlier along the reaction pathway.

**Fig. 5:**
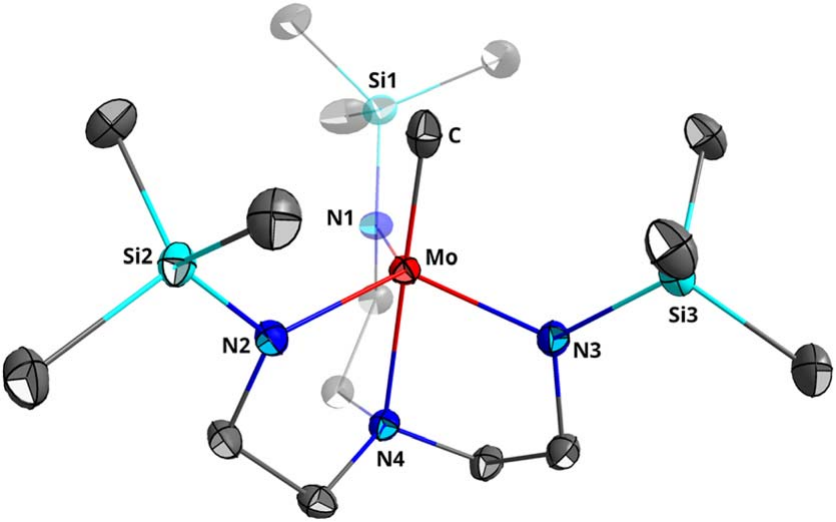
Depiction of the crystal structure of (TMS-TREN)Mo≡CH. Thermal ellipsoids are shown at 50% probability; one of two independent molecules in the unit cell is shown and hydrogen atoms are omitted.

Although (TMS-TREN)Mo≡CH was previously reported by Schrock *et al.*,^19^ it was not crystallographically characterized; in fact, no single crystal structures of molybdenum alkylidyne complexes on a TREN platform, (R′-TREN)Mo≡CR, have been reported. Furthermore, there is a general paucity of terminal transition metal methylidyne crystal structures (16 examples reported in CSD as of April 2025, 4 of which are on molybdenum).^5,7,8,32–37^ Therefore, we carried out a single-crystal X-ray diffraction study of (TMS-TREN)Mo≡CH from crystals grown from a concentrated pentane solution at −35 °C (Fig. 5).

The methylidyne complex crystallized in space group *Pbca* with two crystallographically independent molecules in the asymmetric unit. One of the two molecules suffers from significant disorder in several ligand groups and on the methylidyne moiety; bond lengths are therefore referenced from the less disordered of the two molecules (see SI for further discussion). The refined Mo–C bond length of 1.802(2) Å was slightly longer than that of the only other reported formally Mo(vi) methylidyne complex, the (anilide)_3_Mo≡CH complex shown in Fig. 1G whose Mo–C bond measured 1.703 Å, but is more similar to the Mo–C bond lengths of Mo(v) and Mo(iv) alkylidynes and to substituted Mo(vi) alkylidynes (Table S5). This bond length can also be compared to the much longer Mo–C single bond length of 2.189 Å in the analogous methyl complex (TMS-TREN)MoCD_3_, and to the slightly longer Mo=C double bond length of 1.892 Å in the only crystallographically reported molybdenum(vi) methylidene complex.^38^ The Mo–N bond lengths to the equatorial and apical nitrogens (1.993 ± 0.002 Å and 2.325(2) Å, respectively) are similar to those in the methyl complex.^19^

## Conclusions

In conclusion, we have demonstrated the utility of a new anthracene-releasing methylidyne transfer reagent. The facile preparation of MA-Li from the readily synthesized precursor MA-I should make it accessible to researchers interested in testing its reactivity on new systems of interest. The methanoanthracene (MA) moiety can in principle be installed on any system that will react with alkyllithium and/or alkyl Grignard reagents to give the corresponding alkyl complexes, making this approach potentially suitable to a broader range of metals and complexes, as compared to previously reported methods for preparing methylidyne complexes. Our computational and kinetic studies suggests that the C–C bond-breaking steps involved can be facile, due in part to spin delocalization across the anthracene rings in a radical intermediate.

## Author contributions

R. M., C. I. W., and S. E .C. conducted experiments and analysed data. N. L. and C. E. W. performed theoretical calculations and analysed the results. S. E. C., R. M., N. L. and C. E. W. wrote the manuscript and all authors revised the manuscript. S. E. C. conceived the project. S. E. C. and C. E. W. supervised the work.

## Conflicts of interest

There are no conflicts to declare.

## Supplementary Material

SC-OLF-D5SC07469J-s001

SC-OLF-D5SC07469J-s002

## Data Availability

CCDC 2486403 (for (TMS-TREN)Mo≡CH) contains the supplementary crystallographic data for this paper.^39^ Supplementary information (SI): experimental procedures, NMR data, kinetic analysis, computational analysis and single crystal X-ray data. See DOI: https://doi.org/10.1039/d5sc07469j.
