# Supplementary material for: A readily accessible CH anion transfer reagent for the preparation of a molybdenum methylidyne complex
Source: Chem Sci. 2026 Feb 19;17(17):8780–6. doi: 10.1039/d5sc07469j (PMC12986425; doi:10.1039/d5sc07469j)
Supplement: SC-017-D5SC07469J-s001 [file SC-017-D5SC07469J-s001.pdf]

Supplementary Information (SI) for Chemical Science.
This journal is © The Royal Society of Chemistry 2026

Supporting Information for

A Readily Accessible CH Anion Transfer Reagent for the Preparation of a Molybdenum Methylidyne Complex

Rajesh Mukkera, Nghia Le, Chandler I. Woo, Charles Edwin Webster,* Sidney E. Creutz*

Department of Chemistry, Mississippi State University, Mississippi State, MS 39762, United States

*To whom correspondence should be addressed: ewebster@chemistry.msstate.edu; screutz@chemistry.msstate.edu

Table of Contents

General Information....S2

Synthesis of **indenylmagnesium bromide** S2

Synthesis of **11-iodo-9,10-dihydro-9,10-methanoanthracene (MA-I)** S3

In-situ preparation of **MA-**Li....S6

Synthesis of (TMS-TREN)Mo≡CH S7

In-situ preparation of **MA**-MgBr....S9

UV-vis data and kinetic analysis....S10

Crystallographic data S17

Additional Computational Details and Results....S21

Calculated UV-Vis Spectra....S26

XYZ Coordinates....S33

Supplemental References....S51

General Information:

All manipulations of air-sensitive compounds and reactions were carried out under an atmosphere of argon or nitrogen using standard Schlenk techniques or in a glovebox (LC Technologies, Inc.). Unless otherwise specified, reactions were conducted with magnetic stirring in oven-dried glassware under an inert atmosphere. Solvents were degassed by sparging with argon and purified by passage over molecular sieves in an LC Technologies Solvent Purification System and stored over 4 Å molecular sieves. Tetrahydrofuran (THF) was distilled over sodium/benzophenone and diethyl ether was additionally filtered over activated alumina before storage. (TMS-TREN)MoCl was prepared according to the previously reported procedure.[1] All other chemicals were purchased commercially from Sigma-Aldrich, TCI, or Alfa Aesar and used without further purification unless otherwise stated below. All the NMR spectra were recorded on a 300 or 500 MHz Bruker AVANCE III and referenced internally using the solvent residual proton and carbon peaks. UV–Vis spectra were recorded on a PerkinElmer Lambda 900 spectrometer. Low-temperature UV-Vis measurements were performed using a Unisoku CoolSpeK UV USP-203-B cryostat. High resolution mass spectrometry data is collected on a Bruker MicroToF-QII instrument using an electron spray ionization source.

I. Synthetic Procedures

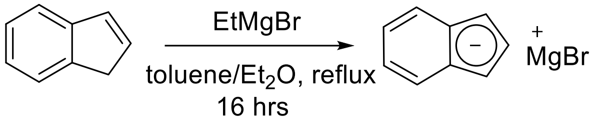

Eq. S1

Indenylmagnesium bromide. The preparation of this compound (Equation S1) was adapted from reported procedures.[2] Under a nitrogen atmosphere, a solution of indene (200 mmol, 23.2 g) in toluene (250 mL) was added dropwise to a solution of EtMgBr in diethyl ether (3 M, 75 mL, 225 mmol) at room temperature. After complete addition, the reaction mixture was refluxed with continuous stirring for 16 h. The reaction mixture was cooled to room temperature, and the solvent was removed *in vacuo* to obtain a pale-yellow solid, which was washed with pentane to obtain indenylmagnesium bromide as an off-white powder (39 g, 88 %). The resulting material was used without further purification.

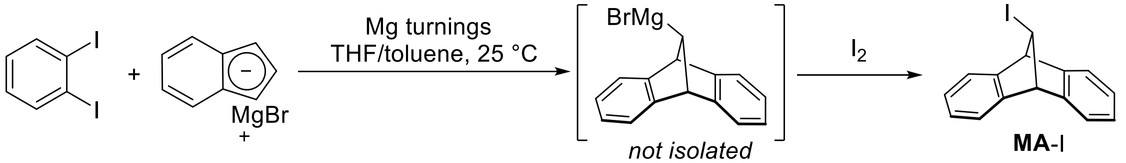

Eq. S2

11-iodo-9,10-dihydro-9,10-methanoanthracene (MA-I). Prepared according to the reaction shown in Equation S2. A 250 mL round-bottom flask was charged with indenylmagnesium bromide (6.58 g, 30 mmol), activated Mg (1.5 g, 37 mmol, activated by treatment with DIBAL-H prior to the reaction[3]), 5 mL of THF and 50 mL of toluene. Separately, an addition funnel was charged with 1,2-diiodobenzene (19.8 g, 60 mmol) and 50 mL toluene, which was added to the reaction mixture slowly dropwise. The reaction mixture was stirred overnight at room temperature and filtered through a pad of Celite on a glass frit to remove residual magnesium turnings. Solid iodine (5 g, 40 mmol) was dissolved in 50 mL Et_2O and added to the solution, followed by stirring for 1 hour. A 10% aqueous

sodium thiosulfate solution (100 mL) was then added to remove excess iodine. The organic phase was separated from the aqueous phase, washed with a saturated brine solution, dried over magnesium sulfate, filtered, and concentrated *in vacuo* to obtain a brown viscous oil mixed with some off-white solid. The desired product is recrystallized from the crude mixture by redissolving it in approximately 10 mL of a 1:1 mixture of acetonitrile and Et_2O and storing the mixture in a freezer for several hours, after which the precipitated crystals are washed with cold Et_2O (2 x 10 mL) and hexane (2 x 10 mL). The resulting light-yellow microcrystalline solid was dried *in vacuo* at 80 °C overnight, giving 2.77 g (8.7 mmol, 29% yield) of **MA**-I. ^{1}H NMR (500 MHz, $CDCl_3$) δ 7.06 (dd, J = 5.3, 3.0 Hz, 2H), 7.01 (dd, J = 5.3, 3.1 Hz, 2H), 4.71 (t, J = 1.5 Hz, 1H), 4.47 (d, J = 1.5 Hz, 2H) ppm. ^{1}H NMR (500 MHz, C_6D_6) δ 7.10 (dd, *J* = 5.3, 3.1 Hz, 2H), 6.92 (dd, *J* = 5.3, 3.1 Hz, 2H), 6.84 (dd, *J* = 5.2, 3.1 Hz, 2H), 6.74 (dd, *J* = 5.3, 3.1 Hz, 2H), 4.27 (t, *J* = 1.5 Hz, 1H), 4.00 (d, *J* = 1.5 Hz, 2H) ppm.^{13}C NMR (126 MHz, $CDCl_3$) δ 147.55, 146.38, 126.08, 125.75, 123.61, 121.65, 59.37, 52.79 ppm. HRMS (ESI) calcd for $[C_{15}H_{11}INa]^+$ ($[M+Na]^+$): *m/z* 340.9798; Found: *m/z* 340.9779. Melting point = 225 °C.

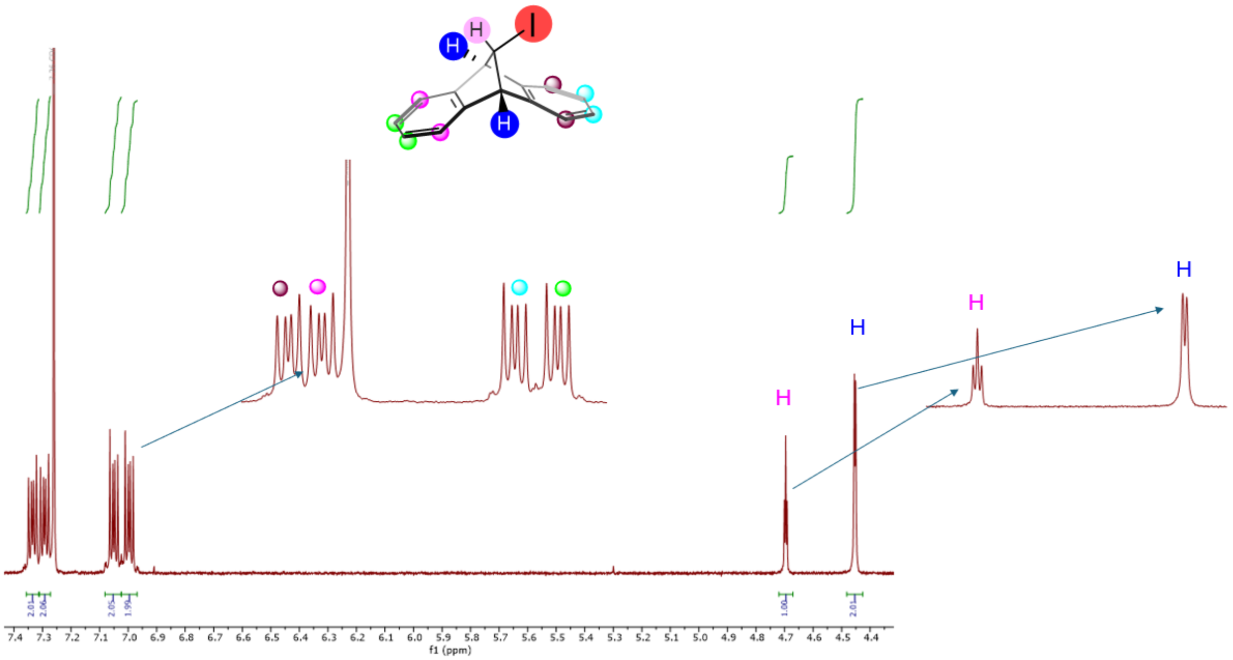

Figure S1. ^{1}H NMR spectrum (500 MHz, 298 K) of 11-iodo-9,10-dihydro-9,10-methanoanthracene (**MA**-I) in $CDCl_3$. Peak assignments were determined based on HMBC (Figure S3).

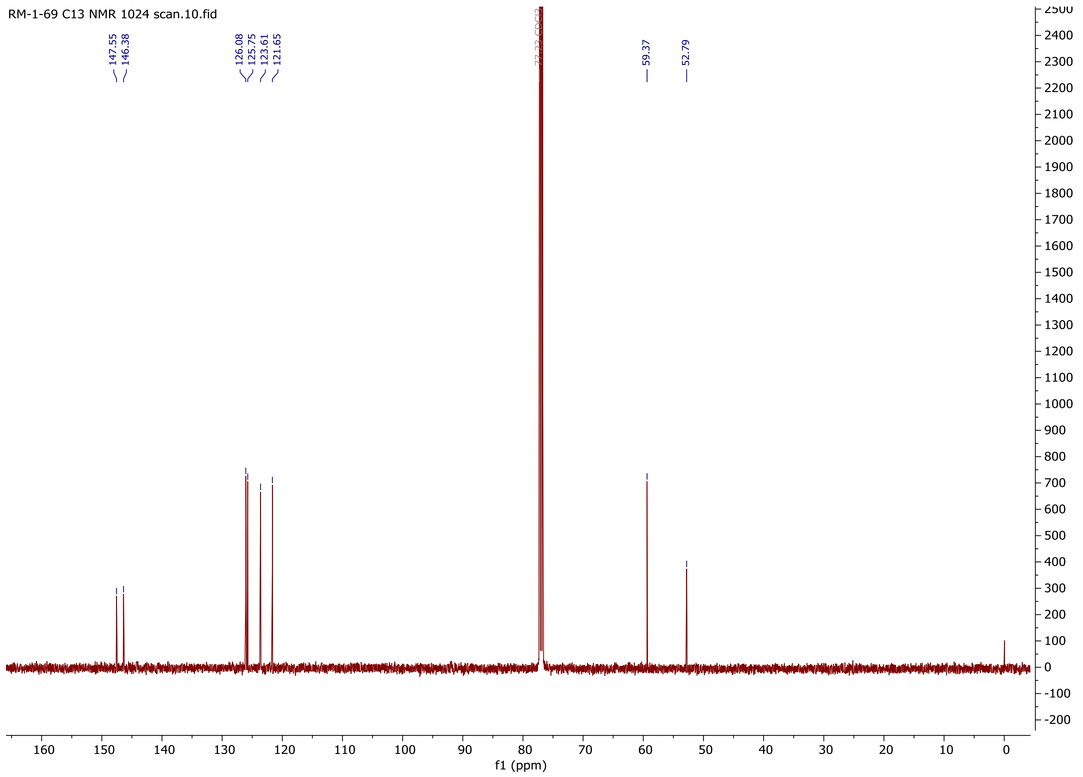

Figure S2. ^{13}C NMR spectrum (126 MHz, 298 K) of 11-iodo-9,10-dihydro-9,10-methanoanthracene (**MA**-I) in $CDCl_3$.

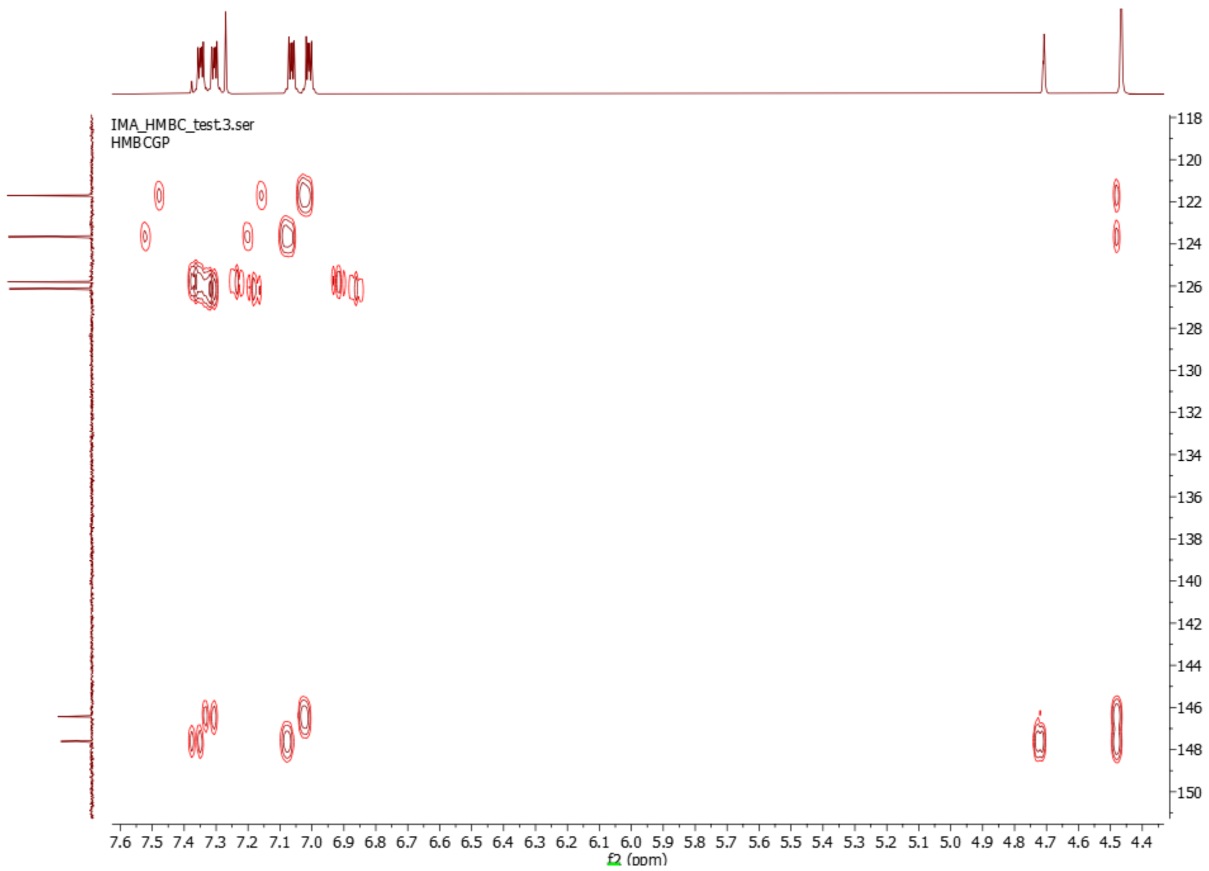

Figure S3. HMBC (^1H-^{13}C) NMR spectrum (500 MHz (^1H), 298 K) of 11-iodo-9,10-dihydro-9,10-methanoanthracene (**MA-I**) in $CDCl_3$.

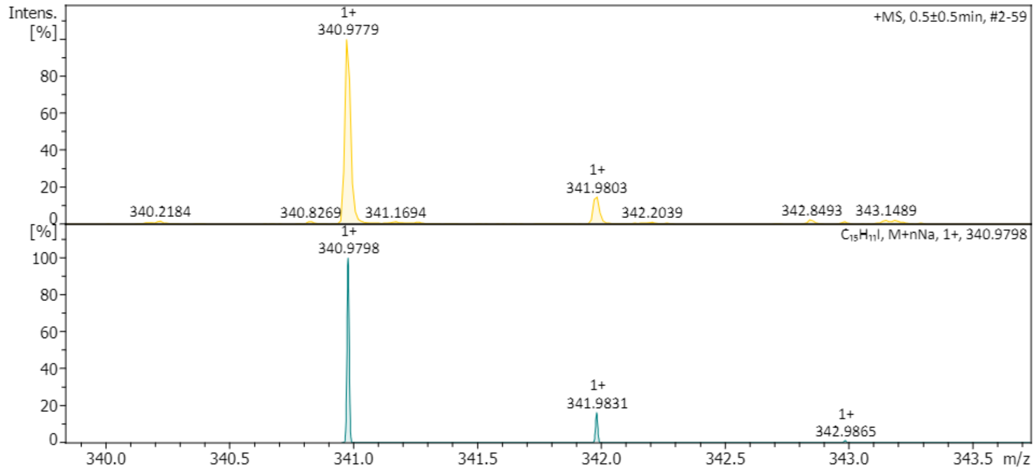

Figure S4. HRMS data for **MA-I**, showing the $[M+Na]^+$ peak. (Top) Data; (Bottom) Simulated data for $[C_{15}H_{11}INa]^+$.

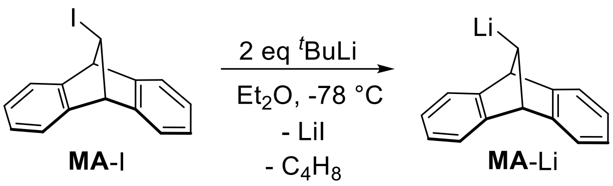

Eq. S3

Preparation of MA-Li (Equation S3): MA-I (10 mg, 0.0314 mmol, 1 equiv) was suspended in approximately 2 mL of diethyl ether in a 20 mL scintillation vial. The suspension was cooled using a -78 °C dry ice/acetone bath. To the cooled suspension, 42 µL of ^{t}BuLi solution (1.5 M in pentane, 0.0628 mmol, 2.0 equiv) was added dropwise. Within a few seconds, the suspension transformed into a homogeneous yellow solution. This solution, which was maintained at -78 °C, was typically used directly for subsequent reactions as described below.

Successful lithiation could be confirmed by quenching the reaction solution with D_2O, which is expected to form the deuterated species **MA**-D (Figure S5). After stirring for 30 minutes at -78 °C, a drop of D_2O was added to the cold reaction mixture; then, additional diethyl ether (5 mL) and deionized water (5 mL) were added to the quenched solution and mixed. The Et_2O layer was decanted from the water, dried over magnesium sulfate, filtered, and concentrated *in vacuo*. The resulting residue was analyzed by ^{1}H NMR, which confirmed that the **MA-I** starting material was completely consumed. By comparison to the known spectrum of 9,10-dihydro-9,10-methanoanthracene (**MA**-H),[4] successful dehalogenation of **MA-I** is confirmed. A 27:1 mixture of the deuterated **MA**-D and protonated **MA-**H are observed, which can be identified by the characteristic resonances of the proton(s) on the bridgehead carbon; the signal appears at 2.54 ppm as a well-resolved triplet for **MA**-H, and at 2.52 ppm as a poorly resolved triplet (broadened by coupling to deuterium) for **MA**-D. The presence of some **MA**-H may be due to side reactions of **MA**-Li with solvent (*vide infra*) or reaction with trace H_2O/HDO during quenching.

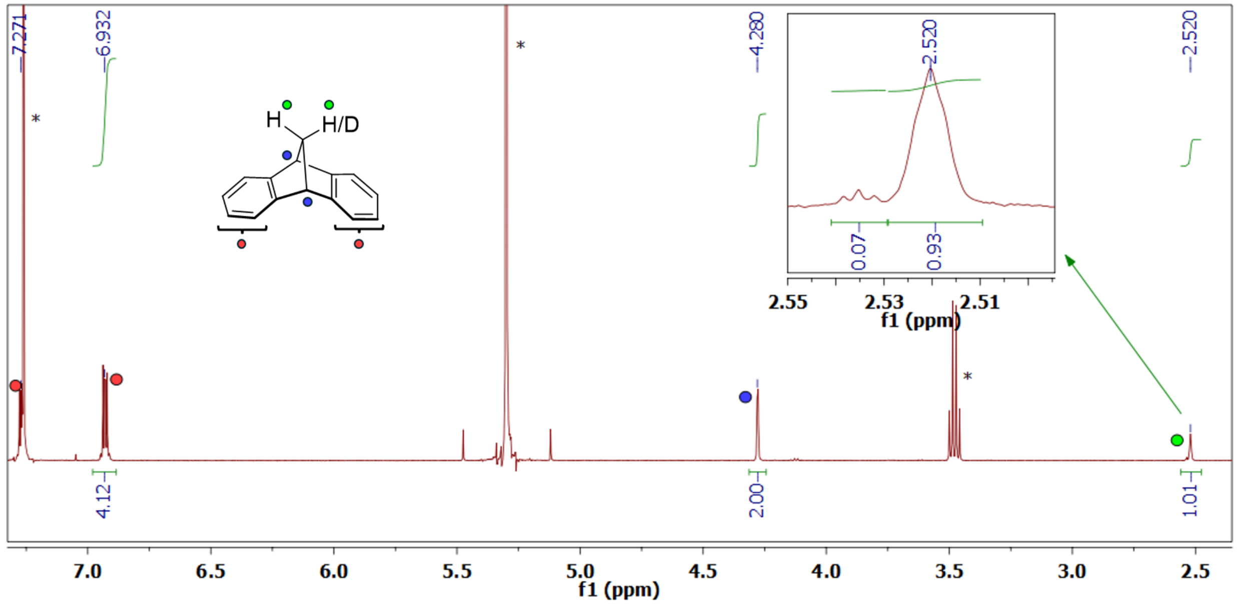

Figure S5. ^{1}H NMR spectrum (500 MHz, 298 K, $CDCl_3$) of the reaction of ^{t}BuLi with **MA-I**, quenched with D_2O after 30 minutes at -78 °C and worked up as described above. * = residual solvents (dichloromethane, diethyl ether, and chloroform).

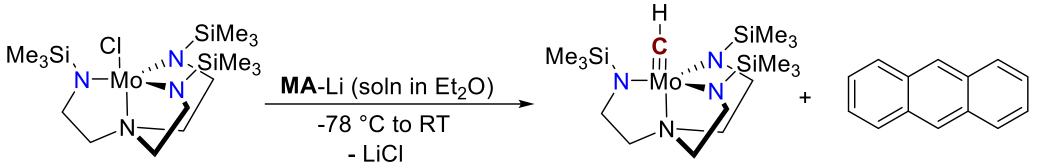

(TMS-TREN)MoCl (TMS-TREN)Mo≡CH

Eq. S4

Formation of (TMS-TREN)Mo≡CH (Equation S4). (TMS-TREN)MoCl (95 mg, 0.193 mmol, 1 eq) was dissolved in 5 mL of diethyl ether in a scintillation vial, forming a bright orange solution. The solution was cooled while stirring using a dry ice-acetone bath at -78 °C. In a separate vial, **MA**-I (125 mg, 0.386 mmol, 2 eq) was suspended in 5 mL of diethyl ether and similarly cooled to -78 °C. To the **MA**-I suspension, ^{t}BuLi solution (0.772 mmol, 0.52 mL, 1.5 M in pentane, 4 equiv) was added dropwise using a 1 mL glass syringe. Within a few seconds, the suspension transformed into a clear yellow solution, indicating the formation of the alkyllithium species (**MA**-Li). The freshly prepared **MA**-Li solution was then added to the pre-cooled (TMS-TREN)MoCl solution. A color change from orange to brown was observed. The reaction mixture was stirred overnight, allowing the reaction mixture to warm slowly from -78 °C to room temperature. The solvent was then removed *in vacuo*. The residue was extracted with toluene and filtered through a Celite pad before the solvent was removed *in vacuo*. The resulting crude product was analyzed by ^{1}H NMR (Figure S6). Addition of 1,3,5-trimethoxybenzene as an internal standard was used to determine that the reaction proceeded in 97% NMR yield. The NMR is consistent with the reported spectrum of (TMS-TREN)Mo≡CH, including the characteristic methylidyne hydrogen peak at 5.55 ppm in the ^{1}H NMR and the methylidyne carbon peak at 283.39 ppm in the ^{13}C NMR.[1] The crude product could be purified further by recrystallization from cold pentane, which removed the **MA**-H impurity; however, we were unable to fully remove the anthracene byproduct from (TMS-TREN)Mo≡CH due to their similar solubility (Figure S7).

X-ray quality single crystals of (TMS-TREN)Mo≡CH were obtained as pale-yellow prisms by layering a concentrated toluene solution of the crude reaction mixture with pentane at room temperature.

Practical note: The protocol described here for the preparation of (TMS-TREN)Mo≡CH uses an excess (2 equiv) of **MA**-Li. While good conversion to the product was still observed with less **MA**-Li (1.2 equiv), a larger excess was used to ensure that no traces of residual (TMS-TREN)MoCl starting material remained. The use of excess **MA**-Li had no apparent detrimental effect other than the formation of increased amounts of the **MA**-H side product, which was readily removed by recrystallization.

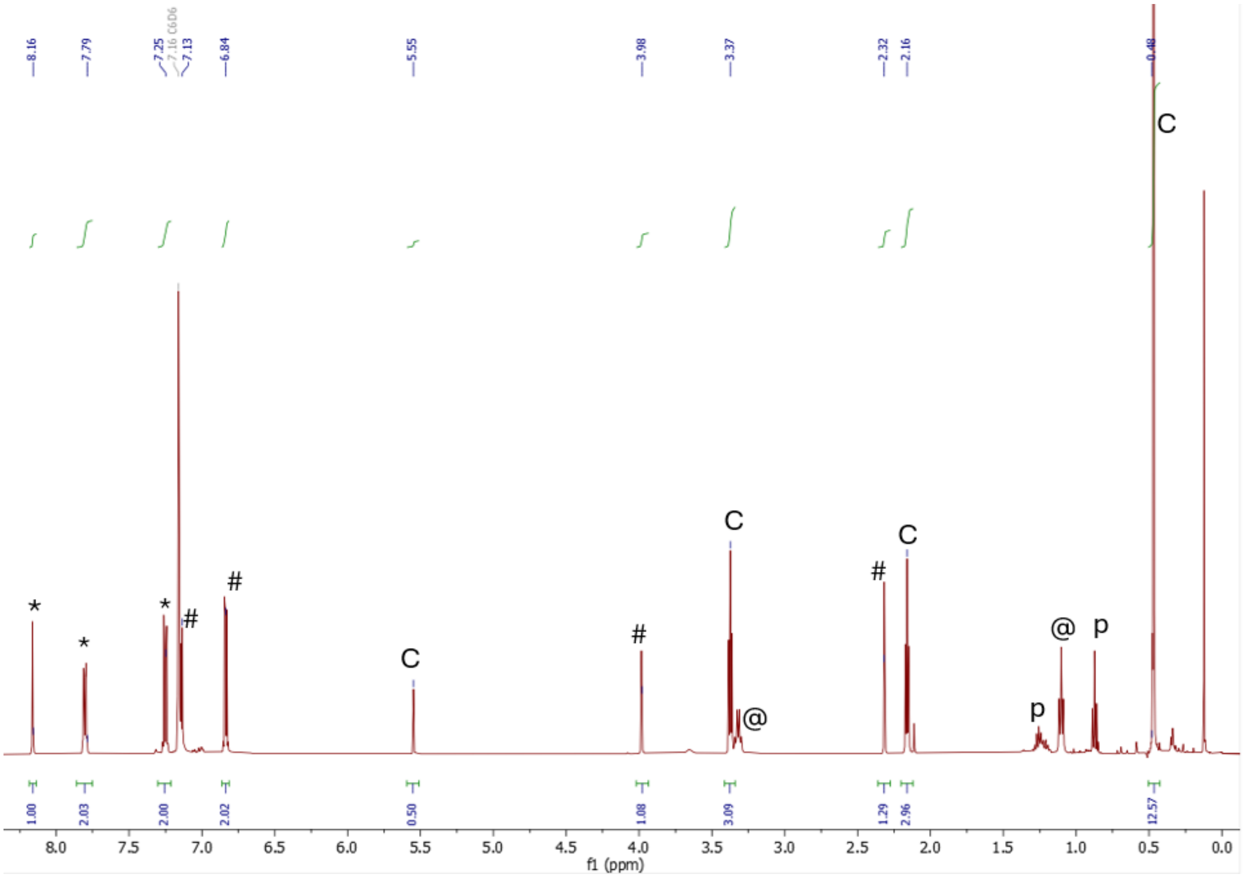

Figure S6. ^{1}H NMR spectrum (500 MHz, 298 K, C_6D_6) of the crude reaction mixture from treatment of (TMS-TREN)MoCl with **MA-**Li showing a 1:1 ratio of the expected products (TMS-TREN)Mo≡CH and anthracene. (* = anthracene; # = **MA**-H; C = (TMS-TREN)Mo≡CH; p = pentane; @ = Et_2O).

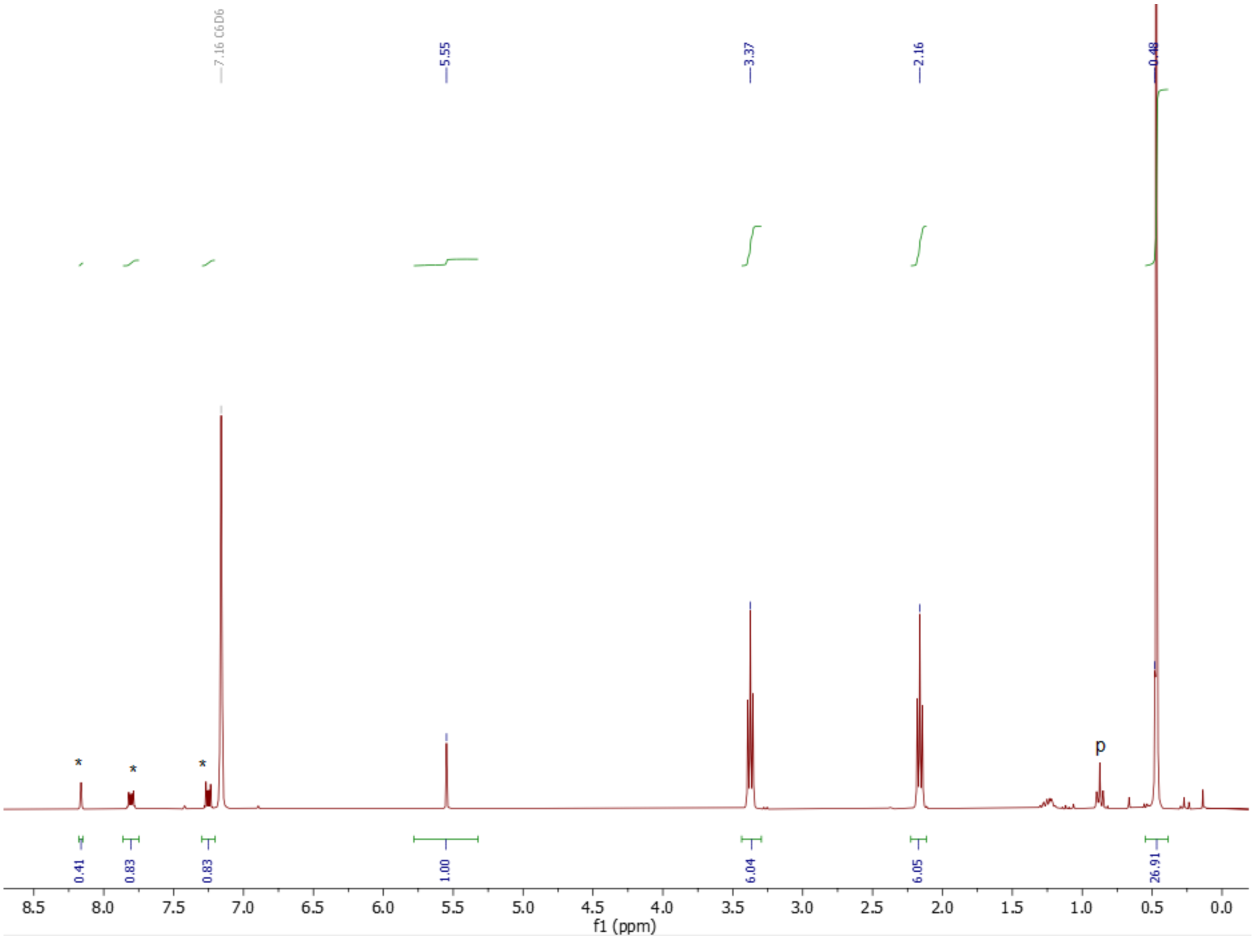

Figure S7. ^{1}H NMR spectrum (500 MHz, 298 K, C_6D_6) from the reaction to form (TMS-TREN)Mo≡CH after recrystallization from pentane (* =anthracene and p = residual pentane).

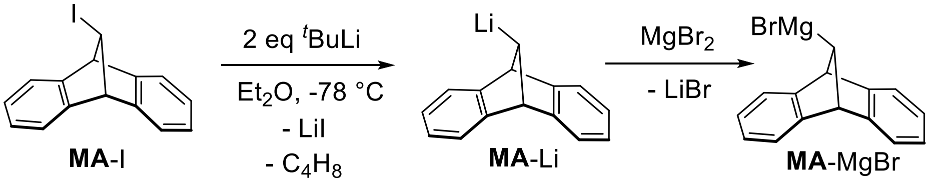

Eq. S5

In-situ preparation and stability of MA-MgBr (Equation S5). MA-I (15.5 mg, 0.0487 mmol) was suspended in 3 mL of diethyl ether (Et_2O) in a 20 mL scintillation vial and cooled to -78 °C. To the cooled solution, 2 equivalents of ^{t}BuLi (1.5 M in pentane, 0.1 mmol) were added dropwise. After 15 minutes, 10 mg of $MgBr_2$ (0.0543 mmol) was introduced. An initial white turbidity was observed upon addition, which gradually cleared to give a transparent solution after 1 hour. The stability of the Grignard species was assessed via D_2O quenching after 3 days and 10 days. NMR analysis (see Figure S8) confirmed approximately 88% formation of **MA**-D at both time points.

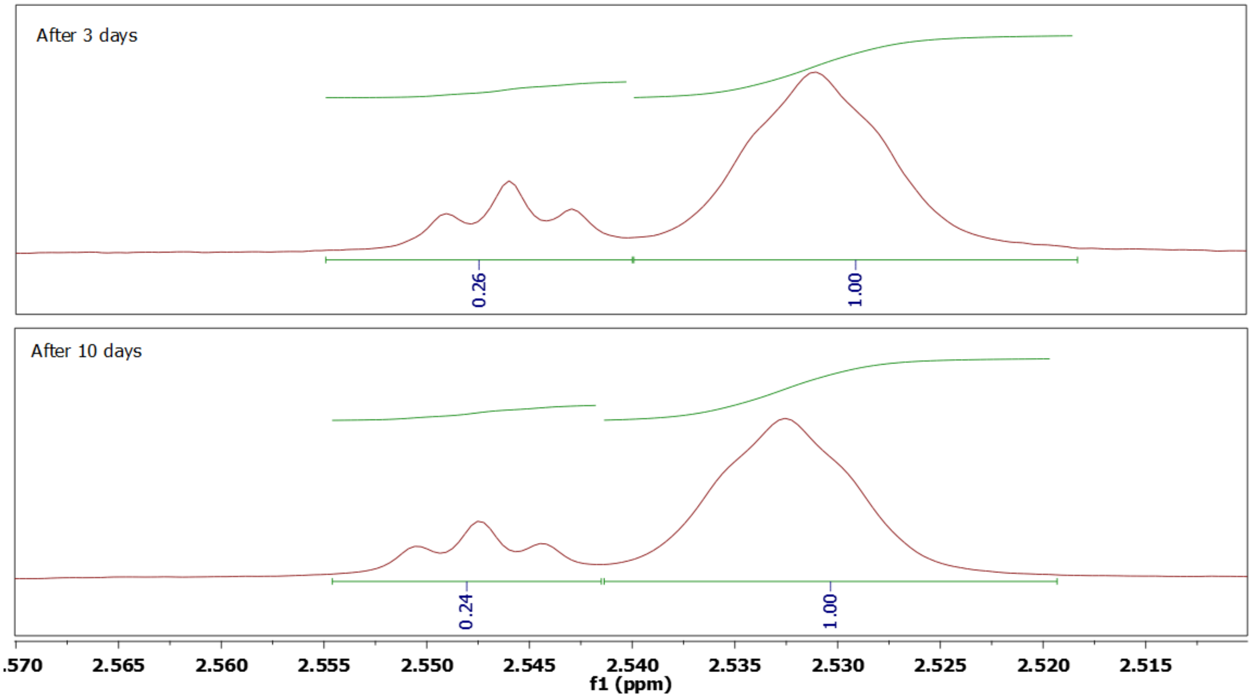

Figure S8. ^{1}H NMR spectrum (500 MHz, 298 K, $CDCl_3$) of the product of quenching **MA**-MgBr (generated *in situ* as described above) with D_2O after storage in Et_2O solution at room temperature for 3 days (top) and 10 days (bottom). The peak shown corresponds to the bridging CH_2 or CHD group, see Figure S5.

II. UV-Vis data and kinetic analyses

Stability of MA-Li at -20 °C. In a 20 mL scintillation vial, a solution of **MA-**Li was prepared by dissolving 10 mg (0.0314 mmol) of iodomethanoanthracene (**MA-I**) in 2.7 mL of diethyl ether, cooling to –78 °C, and adding 2 equivalents of ^{t}BuLi (45 μL, 1.5 M in pentane) dropwise. After 15 minutes, 200 μL of this solution were transferred to a quartz cuvette containing 3 mL of pre-cooled diethyl ether mounted in a cryostat in a UV-Vis spectrometer. The UV-Vis spectrum of the solution was monitored at -20 °C for 4 hours using UV-vis spectroscopy; during this time, the intensity of the spectrum decreased by approximately 10%, indicating slow decomposition (Figure S8). The solution was then allowed to warm to room temperature in the cuvette overnight and the spectrum was recorded again (black spectrum labelled with legend as "overnight at RT" in Figure S8), which suggested complete degradation of the lithiated species.

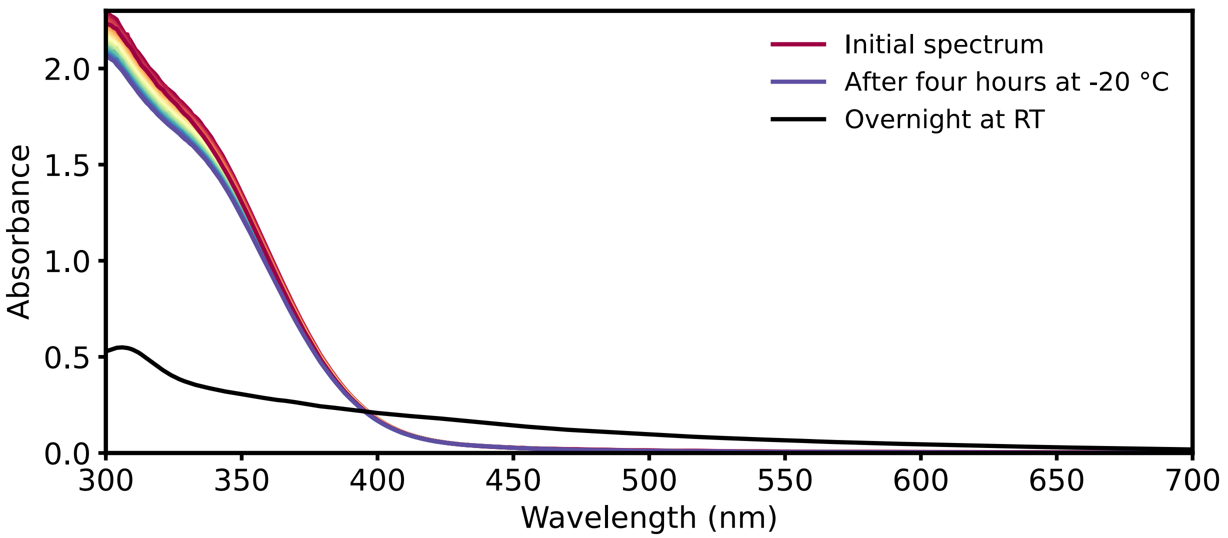

Figure S9. Decomposition of **MA**-Li in Et_2O at -20 °C and room temperature, monitored using UV-Vis spectroscopy.

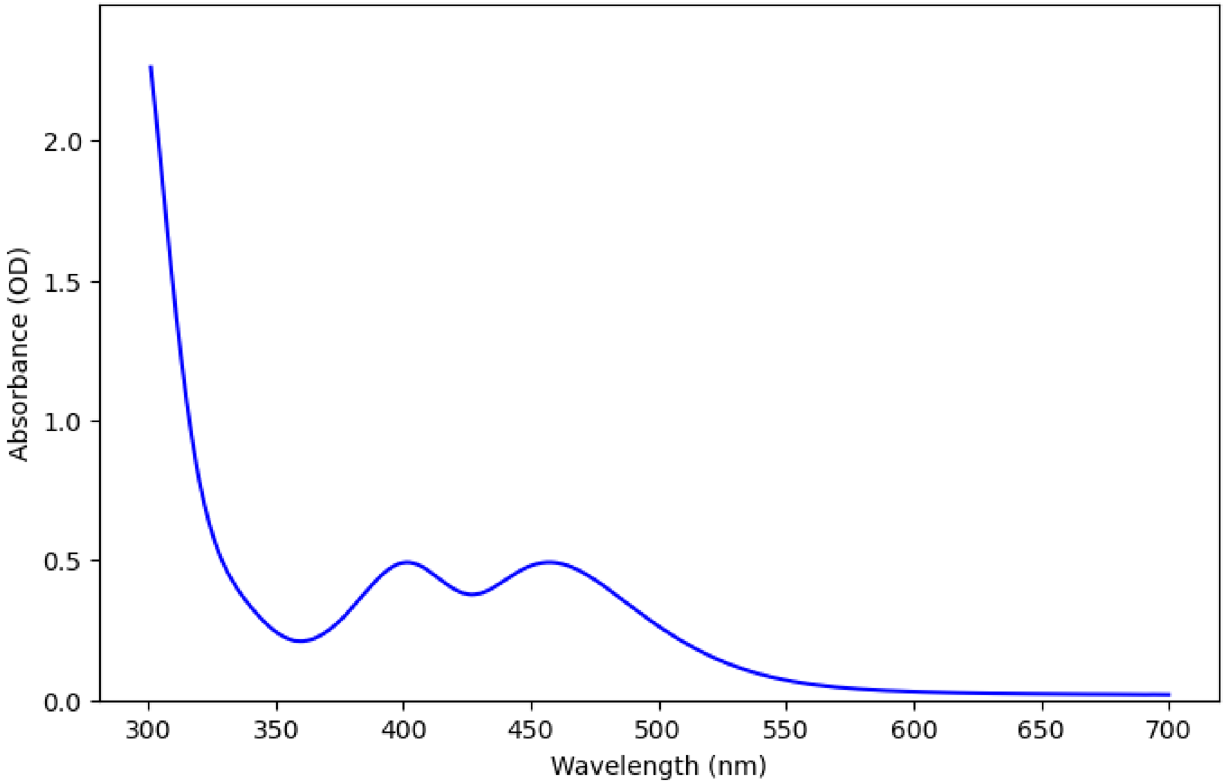

Figure S10. Room-temperature UV-Vis spectrum of (TMS-TREN)MoCl complex (ε = 1105 M^{-1} cm^{-1}, at 457 nm)

In-Situ Reaction Monitoring by Low-Temperature UV-Vis (-20 °C)

Data analysis. The absorbance spectra acquired at each time point, after solvent background subtraction, were decomposed into a linear combination of subspectra for the known components of the reaction; fitting was carried out using the lmfit module (version 1.3.3) implemented in Python with the Levenberg-Marquardt algorithm. Data were typically fit in the region from 340 nm to 550 or 600 nm. The subspectra included in the fit were for anthracene, **MA**-Li, the (L)MoCl starting material, and the (L)Mo≡CH methylidyne product (where L = TMS-TREN). Reference spectra for anthracene and **MA**-Li were measured at -20 °C in separate experiments. For (L)Mo≡CH, since samples were invariably contaminated with anthracene, a reference spectrum was produced by subtracting the anthracene contribution to the spectrum in post-processing, and the extinction coefficient of the complex could be determined after accounting for the known anthracene concentration (from NMR) in the sample; while this may introduce some uncertainty, this species has almost negligible absorbance in the region of the data fitting so this has little effect on the results. For (L)MoCl, a spectrum was measured for each experiment before the addition of the **MA**-Li sample which was used as the reference spectrum for that experiment and used to determine the precise initial concentration based on the measured extinction coefficient of this species. **MA**-H, which is known to form as a byproduct in the reaction, has no significant absorbance in the fitted region, so the spectrum of this species was not considered. For the fitting, the concentration of (L)Mo≡CH was assumed to be equal to the concentration of anthracene and was not independently varied.

The concentrations of anthracene and (L)MoCl extracted from the spectral decomposition were used as inputs into the kinetic data analysis. The initial concentration of **MA**-Li was taken from the experimental conditions, but the **MA**-Li data from the spectral fits was not used as input into the kinetic analysis because of a higher degree of uncertainty in the extinction coefficient of **MA**-Li and its less well-defined absorbance features, as well as the existence of side pathways of **MA**-Li decomposition (see below). Code adapted from the chemical_kinetics python module (https://github.com/flboudoire/chemical-kinetics) was used to model and fit the concentration *vs.* time data; this module sets up a kinetic model and integrates the resulting ordinary differential equations to determine the predicted concentration of each species over time. The lmfit module is then used to fit this to the data. We adapted this module to fit the gradients (first derivative) of the concentrations rather than the actual concentrations, which we found to give a more satisfactory fit the data.

The model used included the following processes:

$$\text{(TMS-TREN)MoCl} + \textbf{MA}\text{-Li} \xrightarrow{k_1} \textbf{Int1} \qquad \textit{Eq. S6}$$

$$\textbf{Int1} \xrightarrow{k_2} \text{(TMS-TREN)Mo}{\equiv}\text{CH} \qquad \textit{Eq. S7}$$

$$\textbf{MA}\text{-Li} \xrightarrow{k_3} \text{(decomp)} \qquad \textit{Eq. S8}$$

In addition to the processes (Eq. S6 and Eq. S7) involving the molybdenum complex, we included an additional process (Eq. S8) for other decomposition processes of **MA**-Li. In our kinetics experiments as well as our control reactions with **MA**-Li alone we have observed that there can be significant variability in this rate; this alkyllithium reagent is air sensitive and the presence of small amounts of adventitious water or oxygen (which could be introduced during sample transfer when the reagent is injected, for example) likely affect this rate. Omitting this process from the kinetic model resulted in notably worse fits to the data, although the order-of-magnitude of the values of k_1 and k_2 extracted from the fit didn't change. Table S1 below summarizes the rate constants

determined for experiments with different initial concentrations of **MA**-Li and (L)MoCl, while Figures S11-S15 show data and fits for experiments with different initial concentrations of reactants.

Table S1. Kinetic Parameters for Reactions of (TMS-TREN)MoCl with MA-Li

Experiment Code	Concentration of (L)MoCl (mM)	Concentration of **MA**-Li (mM)	k_1 ($M^{-1}s^{-1}$)	k_2 (s^{-1})	k_3 (s^{-1})
RM148	0.17	0.43	29±2	0.10±0.01	$1.9 \pm 1 \times 10^{-3}$
RM156	0.34	0.43	16±1	0.14±0.02	$1 \pm 0.2 \times 10^{-2}$
RM157	0.14	1.23	23±2	0.16±0.03	$1.7 \pm 0.3 \times 10^{-2}$
RM158	0.194	0.86	23±1	0.14±0.01	$6 \pm 3 \times 10^{-3}$
RM160	0.16	0.43	35±2	0.17±0.03	$1 \pm 0.1 \times 10^{-2}$
Average	--	--	25±6	0.14±0.02	--

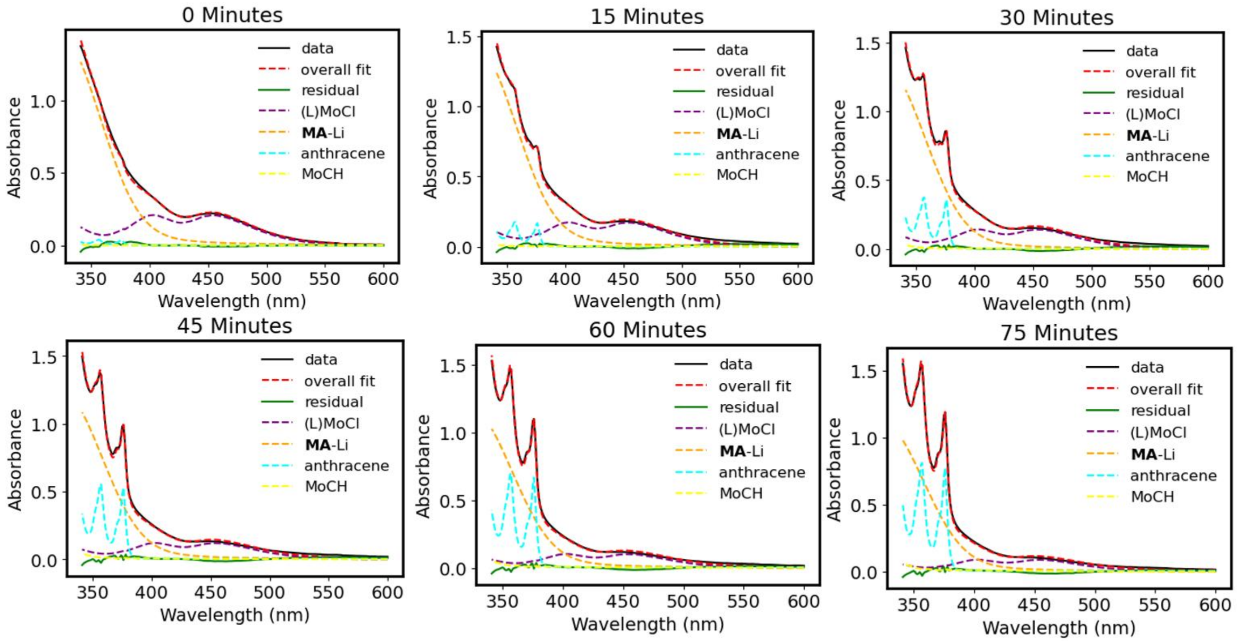

Figure S11. Spectra and spectral decomposition fits for several timepoints from a reaction at -20 °C with initial concentrations of 0.14 mM (TMS-TREN)MoCl and 0.43 mM **MA**-Li (experiment code RM148). Fits of the concentration and kinetic model for this reaction were given in the main text of this article.

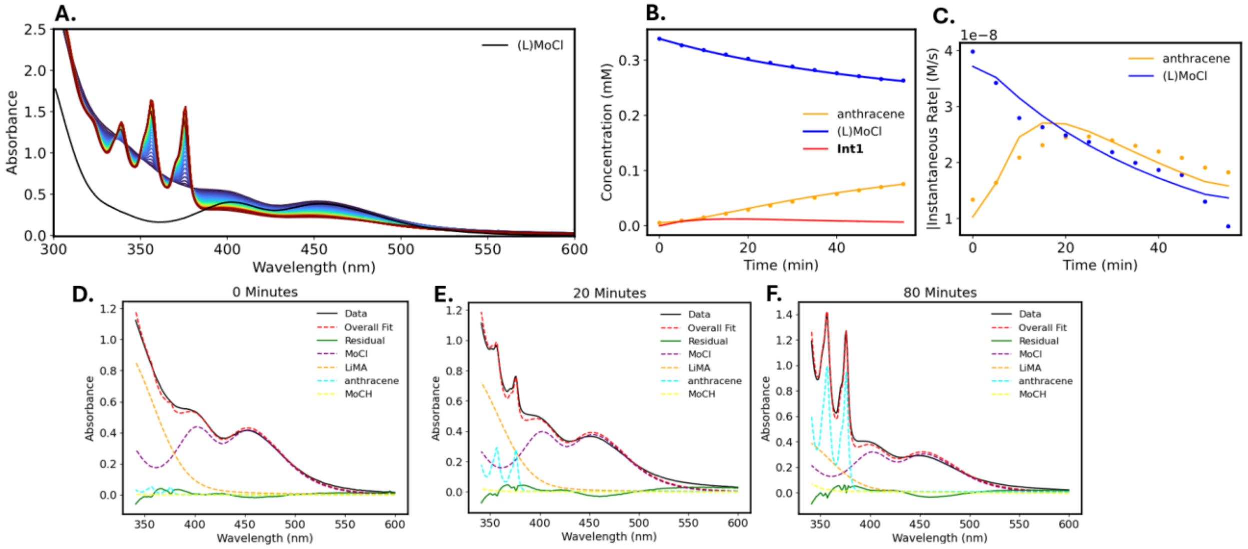

Figure S12. Spectra (A) and spectral decomposition fits for several timepoints (D-F) from a reaction at -20 °C with initial concentrations of 0.27 mM (TMS-TREN)MoCl and 0.43 mM **MA**-Li (experiment code RM156). In panel A, the black line shows the spectrum of (TMS-TREN)MoCl prior to injection of **MA**-Li; solid-colored spectra show reaction after injection at 5-minute intervals (progressing from blue to red). Panel B shows the concentrations of anthracene and (TMS-TREN)MoCl (L = TMS-TREN) over the first 60 minutes of reaction. Dots represent measured concentrations determined from analysis of the UV-Vis spectra; lines represent calculated concentrations based on the kinetic model described in the text. The calculated concentration of the proposed intermediate **Int-1** is also shown. Panel C shows the gradients of the concentrations of anthracene and (TMS-TREN)MoCl, comparing the values calculated from the measured concentrations (dots) and the calculated model (lines). Plotted as the absolute value to facilitate comparison.

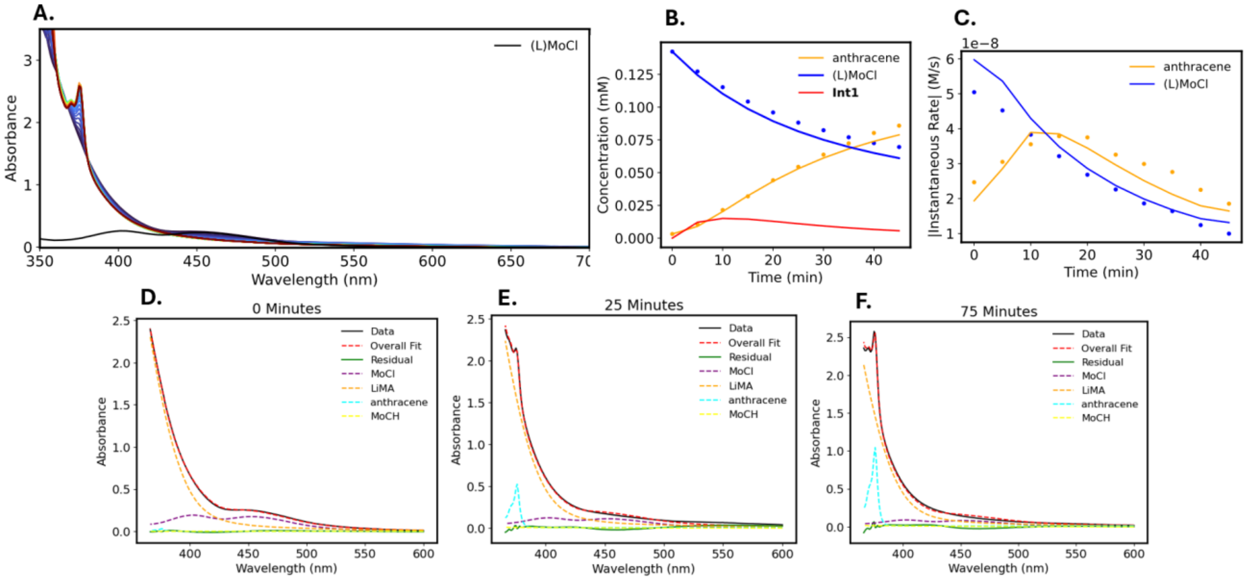

Figure S13. Spectra (A) and spectral decomposition fits for several timepoints (D-F) from a reaction at -20 °C with initial concentrations of 0.12 mM (TMS-TREN)MoCl and 1.23 mM **MA**-Li (experiment code RM157). In panel A, the black line shows the spectrum of (TMS-TREN)MoCl prior to injection of

MA-Li; solid-colored spectra show reaction after injection at 5-minute intervals (progressing from blue to red). Panel B shows the concentrations of anthracene and (TMS-TREN)MoCl (L = TMS-TREN) over the first 60 minutes of reaction. Dots represent measured concentrations determined from analysis of the UV-Vis spectra; lines represent calculated concentrations based on the kinetic model described in the text. The calculated concentration of the proposed intermediate **Int-1** is also shown. Panel C shows the gradients of the concentrations of anthracene and (TMS-TREN)MoCl, comparing the values calculated from the measured concentrations (dots) and the calculated model (lines). Plotted as the absolute value to facilitate comparison.

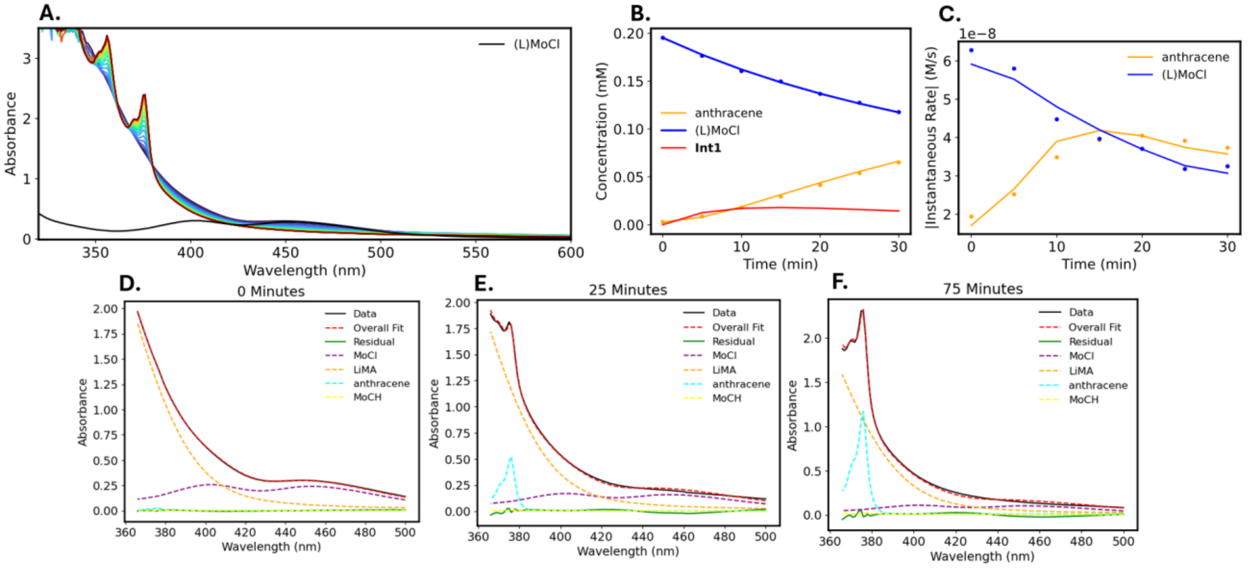

Figure S14. Spectra (A) and spectral decomposition fits for several timepoints (D-F) from a reaction at -20 °C with initial concentrations of 0.16 mM (TMS-TREN)MoCl and 0.86 mM **MA**-Li (experiment code RM158). In panel A, the black line shows the spectrum of (TMS-TREN)MoCl prior to injection of **MA**-Li; solid-colored spectra show reaction after injection at 5-minute intervals (progressing from blue to red). Panel B shows the concentrations of anthracene and (TMS-TREN)MoCl (L = TMS-TREN) over the first 60 minutes of reaction. Dots represent measured concentrations determined from analysis of the UV-Vis spectra; lines represent calculated concentrations based on the kinetic model described in the text. The calculated concentration of the proposed intermediate **Int-1** is also shown. Panel C shows the gradients of the concentrations of anthracene and (TMS-TREN)MoCl, comparing the values calculated from the measured concentrations (dots) and the calculated model (lines). Plotted as the absolute value to facilitate comparison.

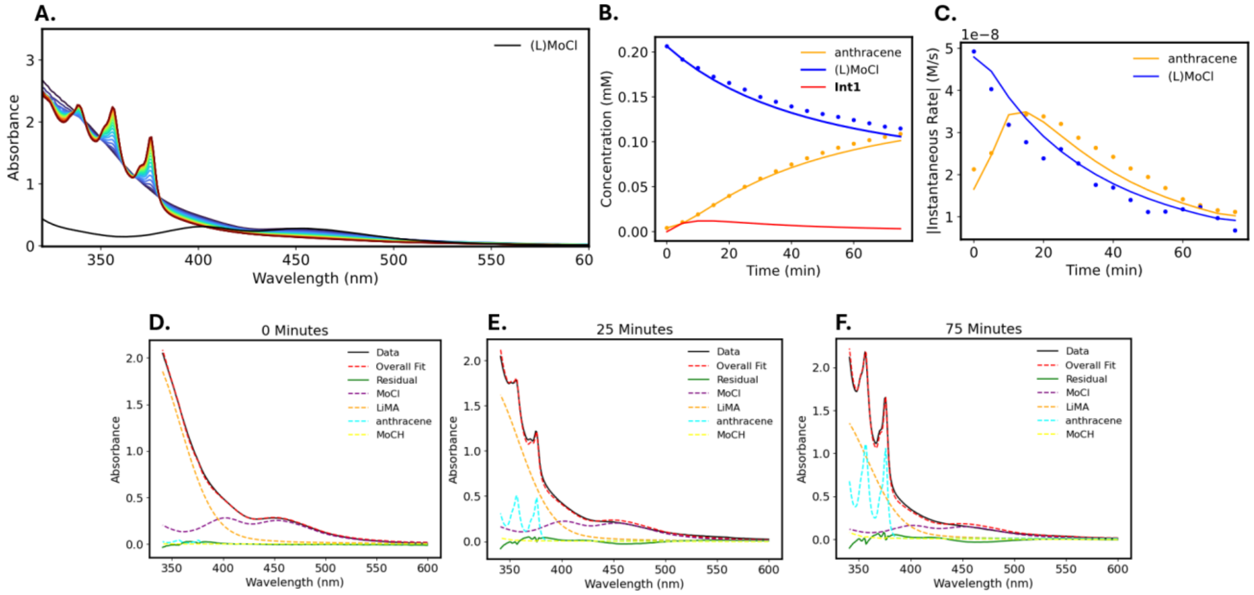

Figure S15. Spectra (A) and spectral decomposition fits for several timepoints (D-F) from a reaction at -20 °C with initial concentrations of 0.16 mM (TMS-TREN)MoCl and 0.43 mM **MA**-Li (experiment code RM160). In panel A, the black line shows the spectrum of (TMS-TREN)MoCl prior to injection of **MA**-Li; solid-colored spectra show reaction after injection at 5-minute intervals (progressing from blue to red). Panel B shows the concentrations of anthracene and (TMS-TREN)MoCl (L = TMS-TREN) over the first 60 minutes of reaction. Dots represent measured concentrations determined from analysis of the UV-Vis spectra; lines represent calculated concentrations based on the kinetic model described in the text. The calculated concentration of the proposed intermediate **Int-1** is also shown. Panel C shows the gradients of the concentrations of anthracene and (TMS-TREN)MoCl, comparing the values calculated from the measured concentrations (dots) and the calculated model (lines). Plotted as the absolute value to facilitate comparison.

III. Crystallographic Details

General crystallographic information. Crystals were mounted on a cryoloop using an oil cryoprotectant. The X-ray intensity data was measured using a three-circle goniometer with a fixed χ angle at 54.75° on a Bruker AXS D8 Venture diffractometer equipped with a Photon III X-ray detector, using molybdenum K_α radiation (λ = 0.7107 Å). The frames were integrated with the help of the Bruker SAINT software using a narrow-frame algorithm.[5] Data were corrected for absorption effects using the multi-scan method with SADABS.[6] Structure solution was carried out using the SHELXT[7] software package. Using the Bruker SHELXTL Software Package, the structure was solved, and refinement of the structures was carried out by least squares procedures on weighted F^2 values using the SHELXL-2019/1.[8] Some refinement tasks were carried out with the assistance of the Olex2[9] GUI. Unless otherwise stated below, all non-hydrogen atoms were refined with anisotropic displacement parameters; all hydrogen atoms were refined isotropically on calculated positions using a riding model with their U_{iso} values constrained to 1.5 times the U_{eq} of their pivot atoms for terminal sp^3 carbon atoms and 1.2 times for all other carbon atoms.

Single crystal X-ray diffraction (SCXRD) for (TMS-TREN)Mo≡CH.

The methylidyne compound (TMS-TREN)Mo≡CH crystallizes from toluene/pentane layering in space group *Pbca* with two independent molecules of the compound in the asymmetric unit. The structure

collected at 100 K (sample code rm-2-113) showed significant unresolved disorder on the methylidyne moiety of one of the two molecules in the asymmetric unit which manifests as an elongated ellipsoid for its atomic displacement parameters (see below; there was also substantial additional disorder in the ligand of this molecule). We initially suspected that this could be due to partial occupancy by an impurity of the starting chloride complex (TMS-TREN)MoCl. To test this, we recollected new data on a new sample (sample code rm-2-181), which was confirmed (by NMR on the same batch of crystals) to be free of (TMS-TREN)MoCl (Figure S16).

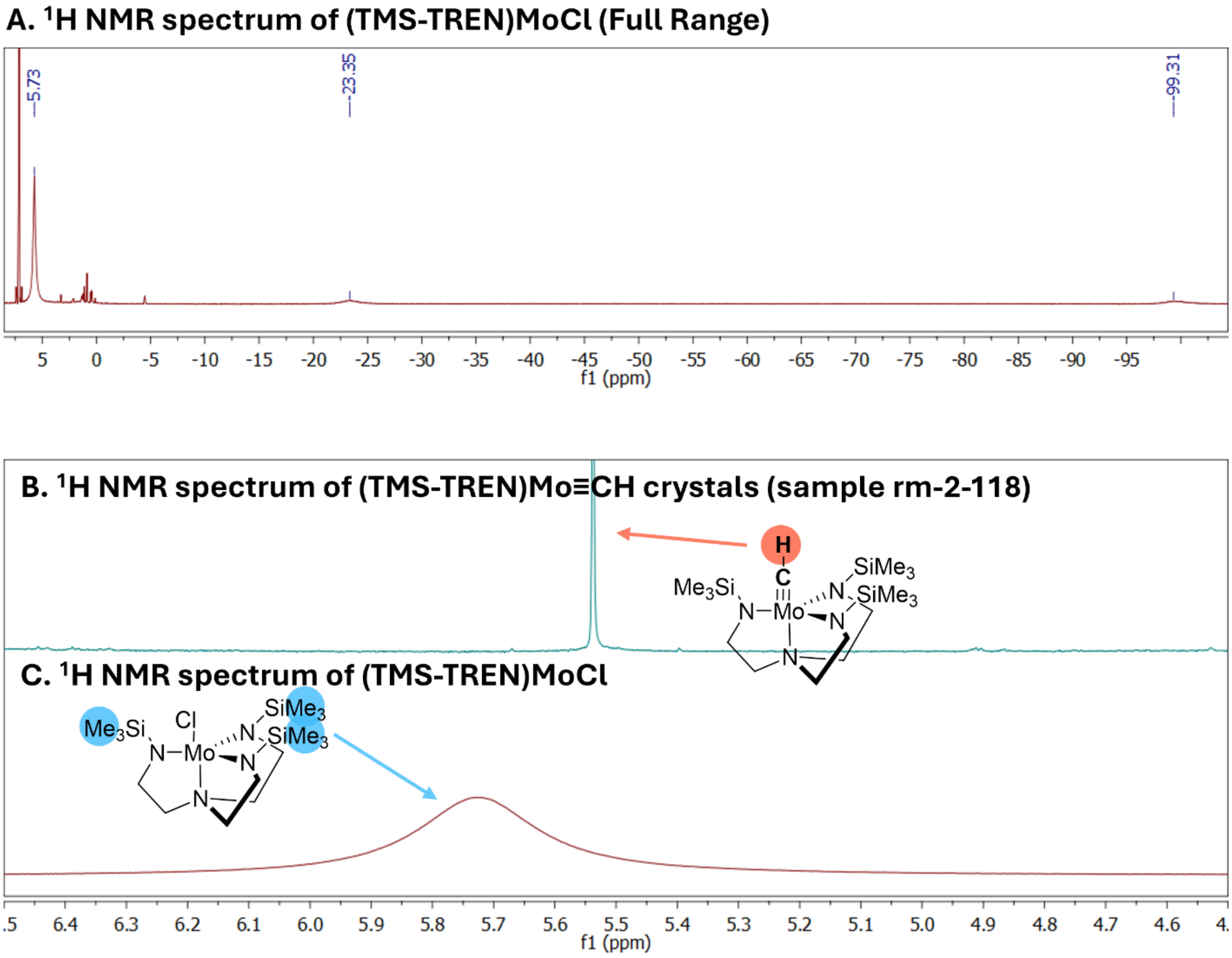

Figure S16. Comparison of the ^{1}H NMR spectra (500 MHz, 298 K, C_6D_6) of (TMS-TREN)Mo≡CH crystals from sample rm-2-181 and the spectrum of the precursor (TMS-TREN)MoCl. (A) Full range of the ^{1}H NMR spectrum of the paramagnetic (TMS-TREN)MoCl complex; the peak at 5.73 ppm is assigned as arising from the -$SiMe_3$ groups. (B) and (C) show a zoom-in of the region between 4.5 ppm and 6.5 ppm where the methylidyne C*H* resonates in (TMS-TREN)Mo≡CH (B, at 5.54 ppm) and the -$SiMe_3$ groups resonate in (TMS-TREN)MoCl. It can be observed in spectrum B that there is no detectable (TMS-TREN)MoCl present in the crystallized sample of the methylidyne complex.

^{1}H NMR data for these crystals (rm-2-181) showed no discernible presence of the chloride complex (Figure S16), or of any compound other than (TMS-TREN)Mo≡CH with some contamination of the bulk sample with anthracene and **MA**-H. Single crystal XRD analysis of a crystal from this batch showed similar disorder as the first sample (rm-2-113); therefore, this suggests that chloride contamination is not the source of the observed disorder, and chloride was not included in the crystallographic

model. However, the XRD data from the second batch of crystals (rm-2-181) was lower-quality overall, so the structural data presented herein is from sample rm-2-113.

SCXRD details for (TMS-TREN)Mo≡CH (100 K, sample code rm-2-113). The complex crystallized as yellow prisms in orthorhombic space group *Pbca* with two independent molecules of the complex in the asymmetric unit. On one of the two molecules, the bridging ethyl groups of the TREN ligand are modeled as disordered over two positions in an 18:82 ratio, and one of the trimethylsilyl groups is modeled as disordered over two positions in a 19:81 ratio. Rigid bond restraints as well as similarity restraints on the atomic displacement parameters of neighboring (1,2 and 1,3) atoms were used to help with modeling the disordered components. ISOR restraints were used on the carbons of the minor component of the disordered trimethylsilyl group and EADP constraints were used to help with modeling the disordered ethyl bridges for carbons lying on the same position. The methylidyne hydrogen was placed at a calculated position using a riding model assuming a linear Mo-C-H angle. Additional metrics for data collection and refinement are provided in Table S2.

Table S2.

**Compound**	(TMS-TREN)Mo≡CH	
**Identification code**	rm-2-113	
**CCDC Deposition Number**	2486403	
**Chemical formula**	$C_{16}H_{40}MoN_4Si_3$	
**Formula weight**	937.46 g/mol	
**Temperature**	100(2) K	
**Wavelength**	0.71073 Å	
**Crystal size**	(0.504 x 0.456 x 0.363) mm^3	
**Crystal system**	orthorhombic	
**Space group**	*Pbca*	
**Unit cell dimensions**	a = 16.8581(17) Å	α = 90°
	b = 16.8406(17) Å	β = 90°
	c = 34.208(3) Å	γ = 90°
**Volume**	9711.8(17) $Å^3$	
**Z**	8	
**Density (calculated)**	1.282 g/cm^3	
**Absorption coefficient**	0.695 mm^{-1}	
**F(000)**	3968	
**Theta range for data collection**	1.81 to 30.529°	
**Index ranges**	-24<=h<=24, -23<=k<=24, -48<=l<=48	
**Reflections collected**	397235	
**Independent reflections**	14811 [R(int) = 0.0827]	
**Coverage of independent reflections**	99.8%	

**Max. and min. transmission**	0.787 and 0.721	
**Refinement method**	Full-matrix least-squares on F^2	
**Refinement program**	SHELXL-2019 (Sheldrick, 2019)	
**Function minimized**	$\Sigma w(F_o^2 - F_c^2)^2$	
**Data / restraints / parameters**	14811 / 27 / 528	
**Goodness-of-fit on F^2**	1.063	
**Final R indices**	12741 data; I>2σ(I)	R_1 = 0.0346, wR_2 = 0.0797
	all data	R_1 = 0.0457, wR_2 = 0.0852
**Weighting scheme**	$w=1/[\sigma^2(F_o^2)+(0.0315P)^2+13.0914P]$ where $P=(F_o^2+2F_c^2)/3$	
**Largest diff. peak and hole**	2.461 and -0.820 eÅ^{-3}	
**R.M.S. deviation from mean**	0.156 eÅ^{-3}	

The structure of one of the two independent molecules was shown in the main text (Figure 5). A depiction of the whole asymmetric unit is given in Figure S17 and a depiction of the second independent molecule, including disorder components, is given in Figure S18. Selected bond lengths and angles are given in Tables S3-S4.

Table S3. Metrical parameters for molecule 1 out of 2 in the asymmetric unit of (TMS-TREN)Mo≡CH (sample rm-2-113)

Rm-2-113 Molecule 1			
Bond	Bond length (Å)	Angle	Bond angle (°)
Mo1-C1	1.802(2)	C1-Mo1-N4	179.51(8)
Mo1-N1	1.991(2)	C1-Mo1-N1	100.87(8)
Mo1-N2	1.992(2)	C1-Mo1-N2	101.18(8)
Mo1-N3	1.995(2)	C1-Mo1-N3	100.23(8)
Mo1-N4	2.324(2)	Mo1-N1-Si1	124.47(9)
N1-Si1	1.755(2)	Mo1-N2-Si2	124.13(9)
N2-Si2	1.749(2)	Mo1-N3-Si3	123.82(9)
N3-Si3	1.749(2)		

Table S4. Metrical parameters for molecule 2 out of 2 in the asymmetric unit of (TMS-TREN)Mo≡CH (sample rm-2-113)

Rm-2-113-Molecule 2			
Bond	Bond length (Å)	Angle	Bond angle (°)
Mo1A-C1A	1.877(3)	C1A-Mo1A-N4A	178.60(8)
Mo1A-N1A	1.9804(16)	C1A-Mo1A-N1A	101.59(8)
Mo1A-N2A	1.9967(18)	C1A-Mo1A-N2A	100.12(9)

Mo1A-N3A	1.9928(17)	C1A-Mo1A-N3A	100.23(8)
Mo1A-N4A	2.3031(17)	Mo1A-N1A-Si1A	124.23(9)
N1A-Si1A	1.7512(18)	Mo1A-N2A-Si2A	125.59(13)
N2A-Si2A	1.7492(17)	Mo1A-N3A-Si3A	125.77(10)
N3A-Si3A	1.7470(18)		

Table S5. Previously Reported Crystallographically Characterized Terminal Molybdenum Methylidyne Complexes and Mo-C Bond Lengths

Compound name in paper	Mo≡CH bond distance (Å)	Formal oxidation state of Mo	Reference
**1-CH**	1.702(5)	6	[10]
**4**	1.764(2)	4	[11]
**4'**	1.781(2)	4	[11]
**[2][BAr$^F_4$]**	1.753(2)	5	[12]

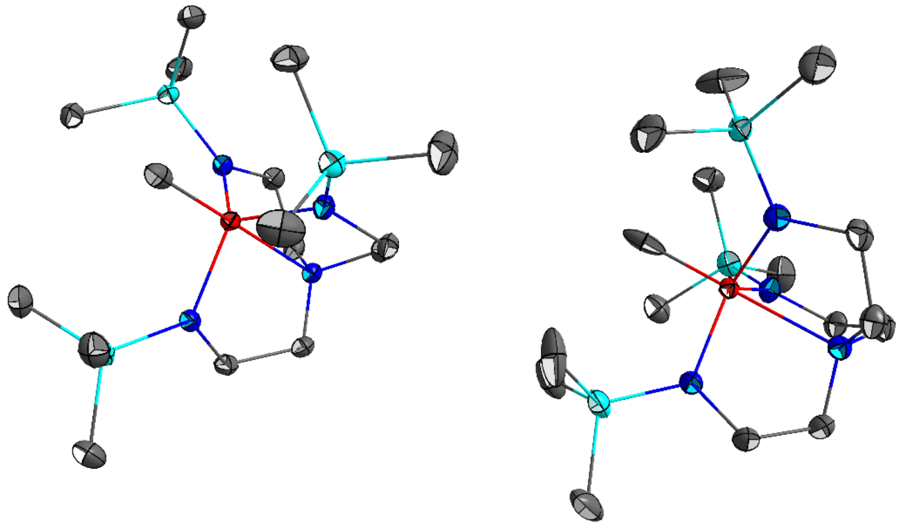

Figure S17. Asymmetric unit contents of the crystal structure of (TMS-TREN)Mo≡CH, sample rm-2-113. Thermal ellipsoids are shown at 50% probability; hydrogens and minor components of disorders are omitted. "Molecule 1", shown in the main text, is on the left in this image and "molecule 2" is on the right.

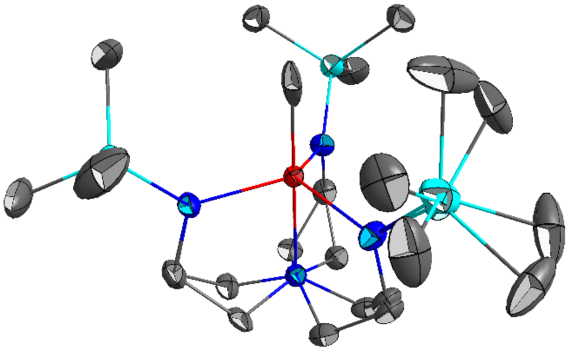

Figure S18. Depiction of "Molecule 2" from the structure of (TMS-TREN)Mo≡CH, sample rm-2-113, showing disordered components of the ethyl bridges and one of the trimethylsilyl groups. Thermal ellipsoids are shown at 50% probability; hydrogens are omitted.

IV. Computational details

Computational studies were performed using the Gaussian 16 package.[13] All stationary points were optimized with density functional theory (DFT) with the PBE0 functional[14,15] along with D3-Grimme dispersion corrections,[16] using basis set BS1. BS1 employed 6-31G(d,p)[17–19] for lighter atoms (Si, C, O, N, Li, H) and LanL2DZ[20–22] for the Mo atom. Solvent effects were included during geometry optimizations via the implicit SMD[23] with parameters for diethyl ether. Transition states were verified by the presence of a single imaginary frequency corresponding to displacement along the reaction coordinate, and each was confirmed to connect the appropriate minima. The minimum-energy crossing points (MECP) were located using the Gaussian interface package, MECPro, developed by Ess and co-workers.[24]

For improved reliability of the computed energetics, single-point energy calculations were performed using the B3LYP[25,26] functional with the def2-TZVP[27] basis set for all atoms, based on geometries optimized at the SMD(diethyl ether)-PBE0-D3/BS1 level theory. Accordingly, the final reported energetic values are at the SMD(diethyl ether)-B3LYP-D3/def2-TZVP//SMD(diethyl ether)-PBE0-D3/BS1 level theory, abbreviated as SMD (diethyl ether)-B3LYP-D3/def2-TZVP//PBE0/BS1.

We searched for open-shell singlet states to probe possible diradical character in the intermediates and transition states. Notably, for **Int2**, the open-shell singlet solution (**OSS-Int2**) is significantly more stable, indicating substantial biradical character; this singlet biradical lies slightly lower in energy (by 2.1 kcal/mol) than the corresponding triplet **T-Int2**. The spin density plot in **Figure S22** shows that the unpaired electrons are delocalized over the anthracene π-system and are strongly coupled to the Mo center. This delocalization is similar for **T-Int2** and **OSS-Int2**.

For all other intermediates and transition state species, no lower-energy singlet state with significant diradical character was located in our calculations.

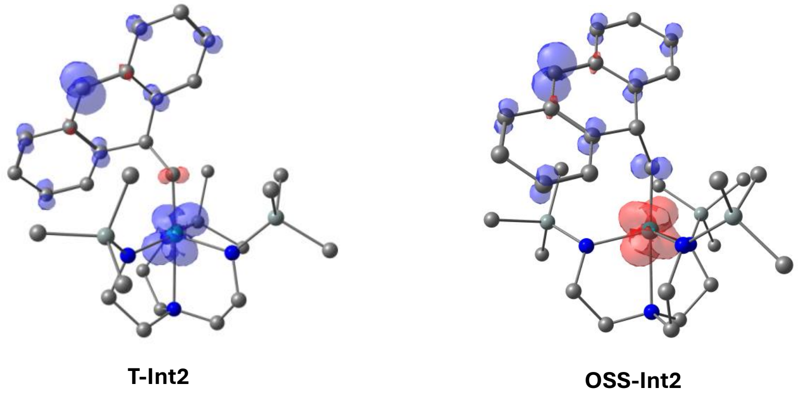

Figure S19. Spin density plots for **Int2** in the triplet (T) and open-shell singlet (OSS) states. Hydrogen atoms are omitted for clarity. Blue and red colors represent α and β spins.

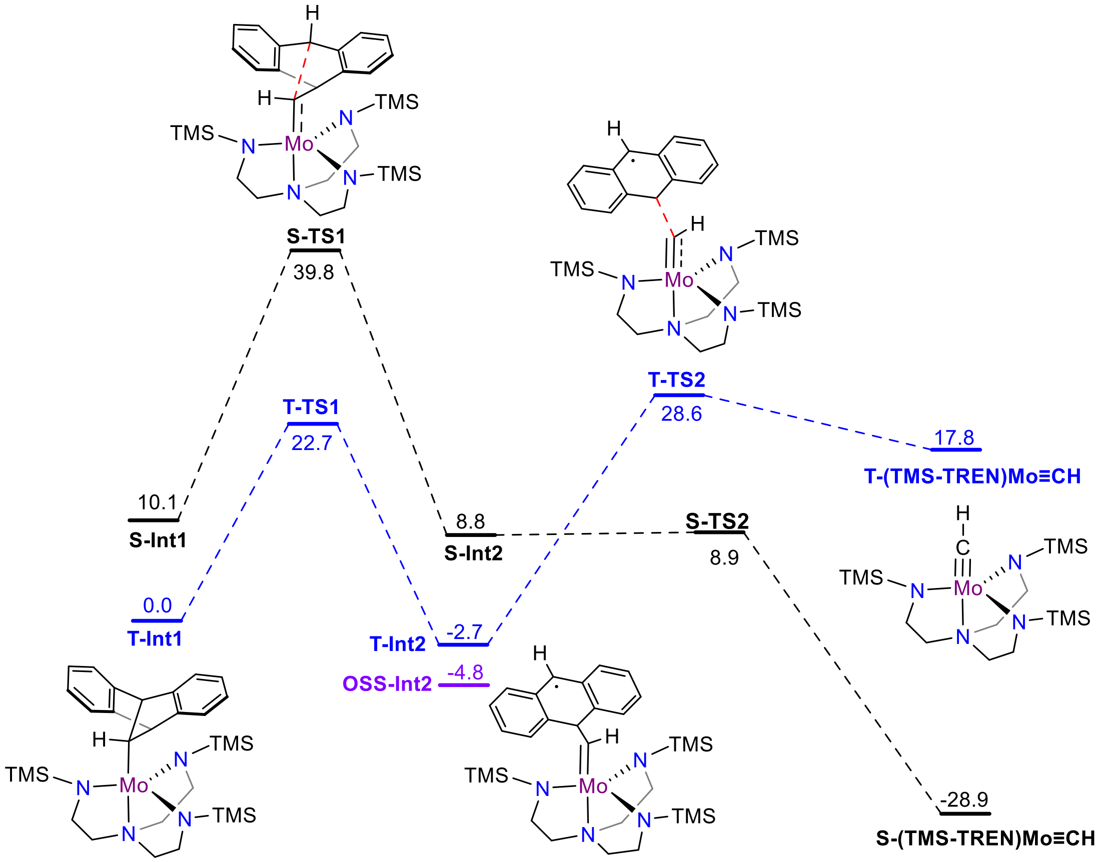

Figure S20. Gibbs free energy diagram (kcal mol^{-1}) at the SMD(diethyl ether)-B3LYP-D3/def2-TZVP//PBE0/BS1 level of theory for the transformation between **Int1** and (TMS-TREN)Mo≡CH.

Effect of lithium coordination. The possible effect of lithium coordination on the stability of the intermediates was considered (see discussion in main text). To account for the energy required to dissociate Li^+ from diethyl ether solvent (Figure S20), which would otherwise require extensive explicit-solvent modeling and is complicated by the uncertainty of how many solvent molecules are coordinated to each lithium ion at given reaction conditions, we used an experimental estimate of approximately 15.0 kcal mol^{-1} for the enthalpy of partial Li^+ desolvation in diethyl ether.[28] This value corresponds to the enthalpy of dissociation of one Et_2O molecule from $Li(Et_2O)_3^+$, and therefore we implicitly assume that two solvent molecules remain coordinated to Li^+ after it coordinates to **Int-1**. There is some uncertainty in this assumption; if more than one Et_2O molecule dissociates from the solvated lithium cation, the energy cost for desolvation will be higher. Consideration of the energetics involved in lithium solvation is further complicated by the presence of iodide in the reaction mixture, which may interact with Li^+ to form various species in Et_2O solution.[29] Because we are primarily interested in how Li^+ coordination is able to stabilize the C-C bond cleavage transition state, we considered further investigation of the lithium binding equilibria and energetics to be beyond the scope of the current investigation.

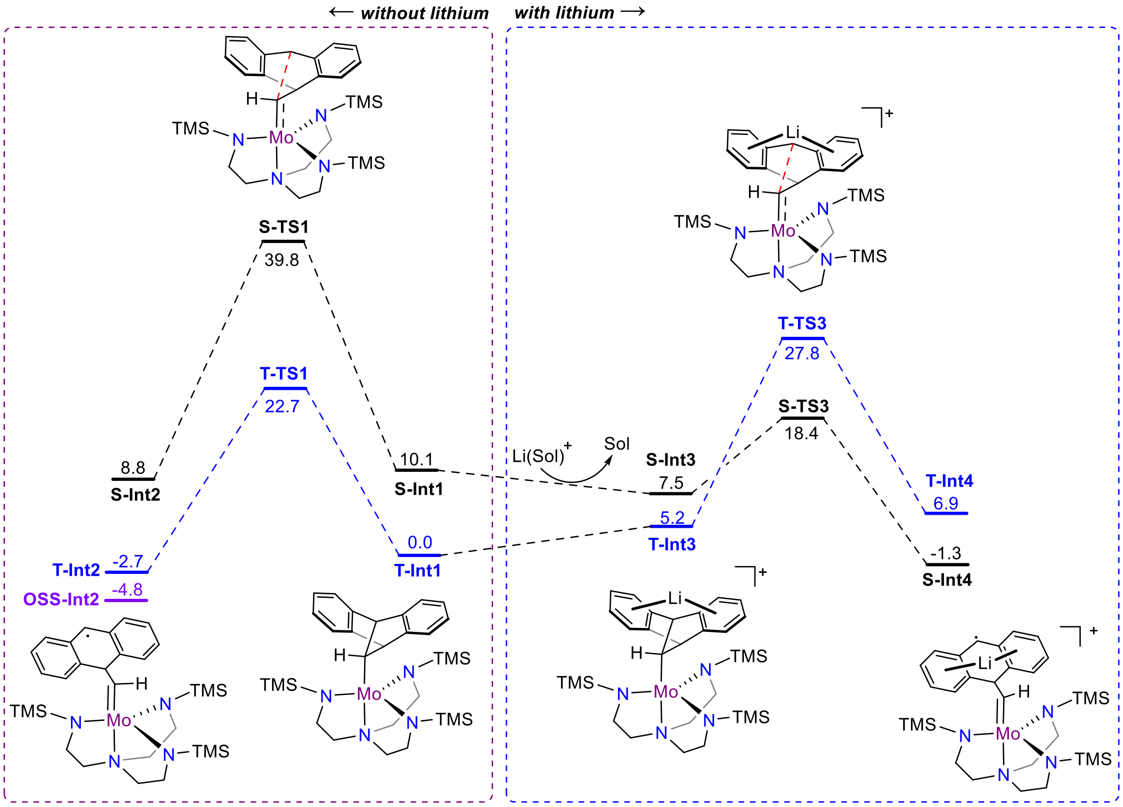

Figure S21. Gibbs free energy (kcal mol^{-1}) comparison of the first C–C bond cleavage with and without involvement of a lithium cation. The report values were computed at the SMD(diethyl ether)-B3LYP-D3/def2-TZVP//PBE0/BS1 level of theory. Solvent molecules coordinated to lithium were not explicitly included in the calculations of the intermediates and $Li(Sol)^+$ is used as a general representation of the possible solvation states of lithium cations in solution. See text above for further discussion.

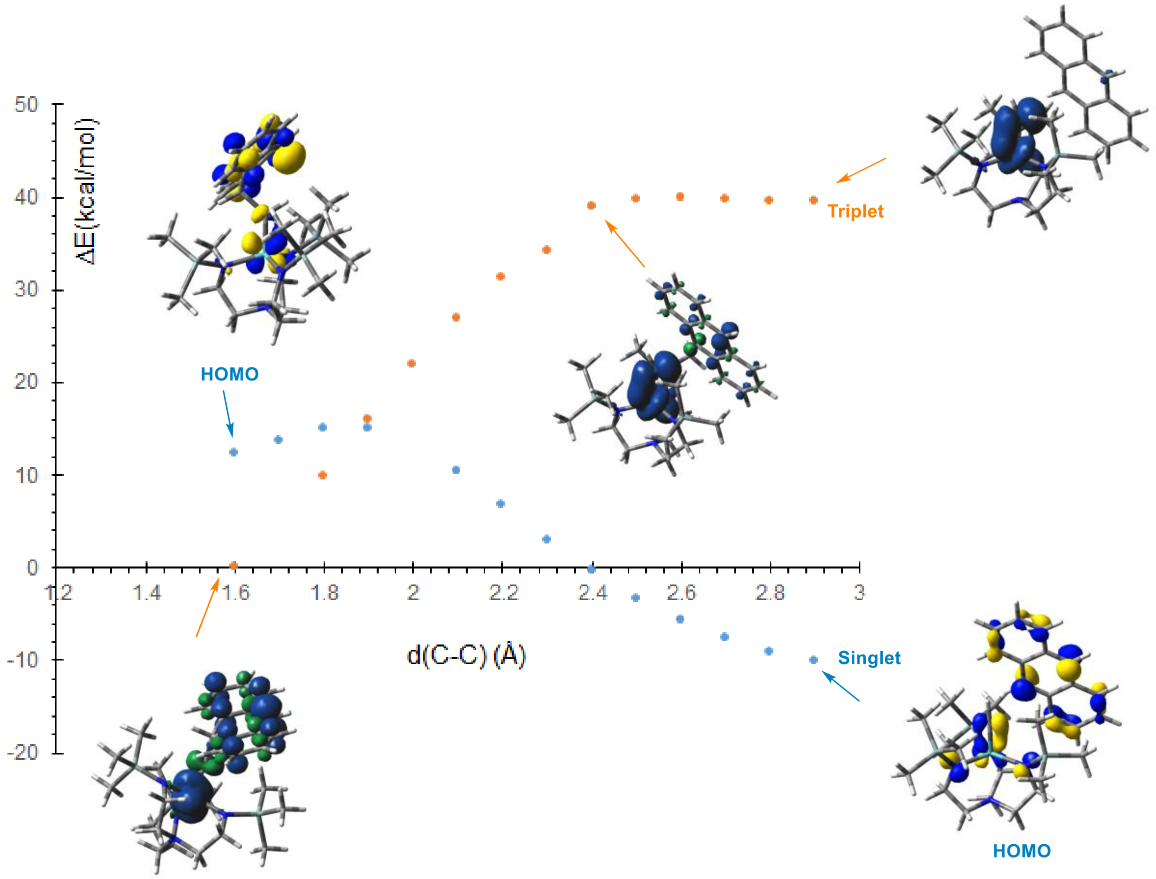

Figure S22. Electronic energy comparison during elongation of the second C–C bond (starting from the triplet diradical **T-Int2**), calculated at the SMD(diethyl ether)-PBE0/BS1 level of theory on constrained surfaces, with spin-density isosurfaces shown for selected points on the triplet surface and HOMO isosurfaces shown for selected points on the singlet surface. Since the open-shell singlet and triplet states of **Int2** are similar in geometry and relative energy, the located minimum energy crossing point for the triplet state is a good approximation of the same crossing point from **OSS-Int2** to the product.

Relatively low barriers for C-C single bond cleavage reactions are fairly rare, especially for unstrained substrates. Table S6 shows some representative examples from the recent literature.

Table S6. Comparison to previously reported calculated energy barriers for C-C bond cleavage reactions from the literature.

Transition Metal	Activation Barrier	Note	Reference
Hf	26.4	β-Aryl elimination, non-strained substrate	[30]
Rh	28.7	β-Aryl elimination, non-strained substrate	[31]
Ni	18.1	Cyclobutanone ring-opening	[32]
Ru	14.4	Cyclopropane ring-opening	[33]

Table S7. Tabulated energetic values for the reported stationary structures located at SMD(diethyl ether)-PBE0/BS1 level of theory, including electronic energy (E), electronic energy with zero-point energy (E + ZPE), enthalpy (H), and Gibbs free energy (G) in Hartree, along with the number of imaginary frequencies for each structure.

Compound	E	E + ZPE	H	G	# Imag Freq
**S-Int1**	-2326.6047	-2325.8546	-2325.8090	-2325.9288	0
**S-Int2**	-2326.5816	-2325.8350	-2325.7886	-2325.9107	0
**S-Int3**	-2334.0241	-2333.2715	-2333.2247	-2333.3461	0
**S-Int4**	-2334.0153	-2333.2645	-2333.2171	-2333.3409	0
**S-(TMS-TREN)Mo≡CH**	-1787.6661	-1787.1175	-1787.0813	-1787.1812	0
**T-Int1**	-2326.6192	-2325.8701	-2325.8242	-2325.9461	0
**T-Int2**	-2326.6025	-2325.8573	-2325.8105	-2325.9346	0
**T-Int3**	-2334.0370	-2333.2795	-2333.2339	-2333.3527	0
**T-Int4**	-2334.0037	-2333.2550	-2333.2072	-2333.3318	0
**T-(TMS-TREN)Mo≡CH**	-1787.5861	-1787.0407	-1787.0036	-1787.1082	0
**S-TS1**	-2326.5534	-2325.8061	-2325.7608	-2325.8796	1
**S-TS2**	-2326.5769	-2325.8319	-2325.7853	-2325.9083	1
**S-TS3**	-2333.9933	-2333.2407	-2333.1947	-2333.3138	1
**T-TS1**	-2326.5663	-2325.8207	-2325.7748	-2325.8968	1
**T-TS2**	-2326.5377	-2325.7954	-2325.7481	-2325.8736	1
**T-TS3**	-2333.9795	-2333.2294	-2333.1841	-2333.3017	1

Calculated UV-Vis Spectra

Excited-state calculations were carried out using time-dependent DFT (TDDFT) with the B3LYP functional and the def2-TZVP basis set at the ground-state geometries. These data are reported at the same SMD(diethyl ether)–B3LYP-D3/def2-TZVP//PBE0/BS1 level of theory as the energetics.

As a benchmark, we calculated the predict UV-Vis spectrum of (TMS-TREN)MoCl; the experimentally measured spectrum of this complex, shown in Figure S10, has two prominent peaks in the visible region at 461 nm and 399 nm, attributed to *d*-*d* transitions. The computationally predicted UV-Vis spectrum of this complex in the triplet state is shown in Figure S23 and reproduces the presence of two low-energy visible absorbances, but shifted to lower energy (549 and 502 nm). This represents an energy offset of approximately -0.4 eV between experiment and computation.

The simulated UV-Vis spectra of **T-Int-1** is shown in Figures S24, and the corresponding NTO analysis is given in Table S9. The predicted lowest-energy transition occurs at 647 nm, somewhat lower energy than the feature observed experimentally (550 nm) that we hypothesized could correspond to this species. However, the energy offset (-0.3 eV) is similar to that observed for the benchmark calculation on the chloride complex.

The simulated UV-Vis spectra and NTO's of **S-Int1** are also given for comparison (Figure S25, Table S10).

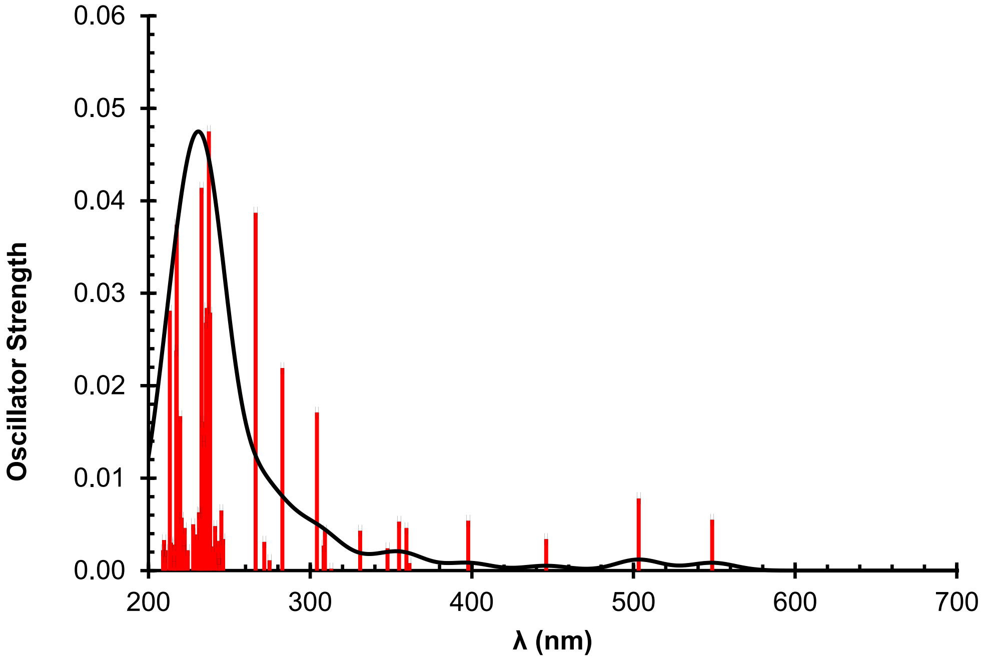

Figure S23. Simulated UV–vis spectrum of (TMS-TREN)MoCl at the SMD(diethyl ether)–B3LYP-D3/def2-TZVP//BS1 level of theory.

Table S8. Selected natural transition orbitals (NTOs) for transitions in (TMS-TREN)MoCl. Hydrogen atoms are omitted for clarity.

Excitation (nm)	HOTO (Highest Occupied Transition Orbital)	LUTO (Lowest Unoccupied Transition Orbital)
λ = 548.7	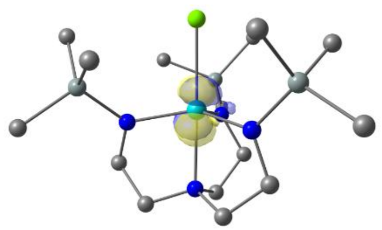	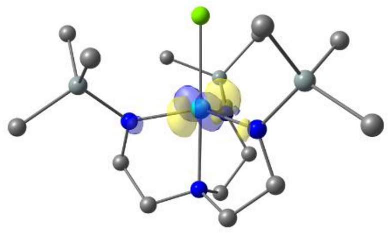
λ = 503.2	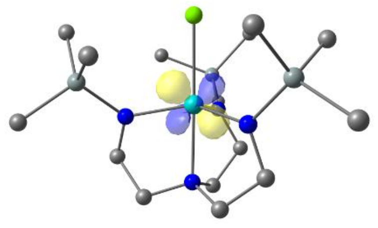	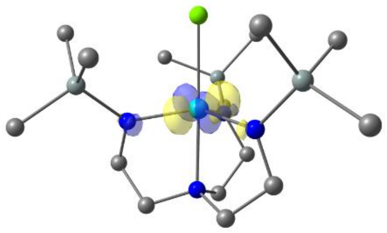

λ = 446.0	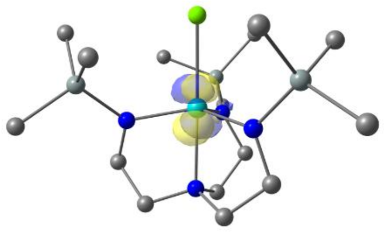	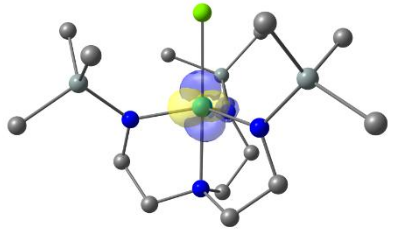
λ = 397.8	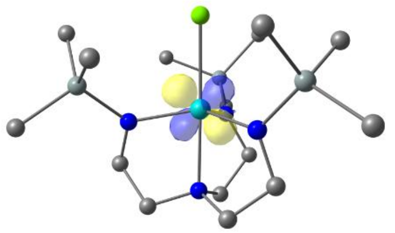	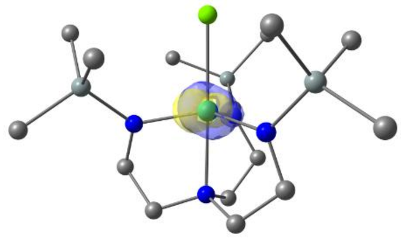

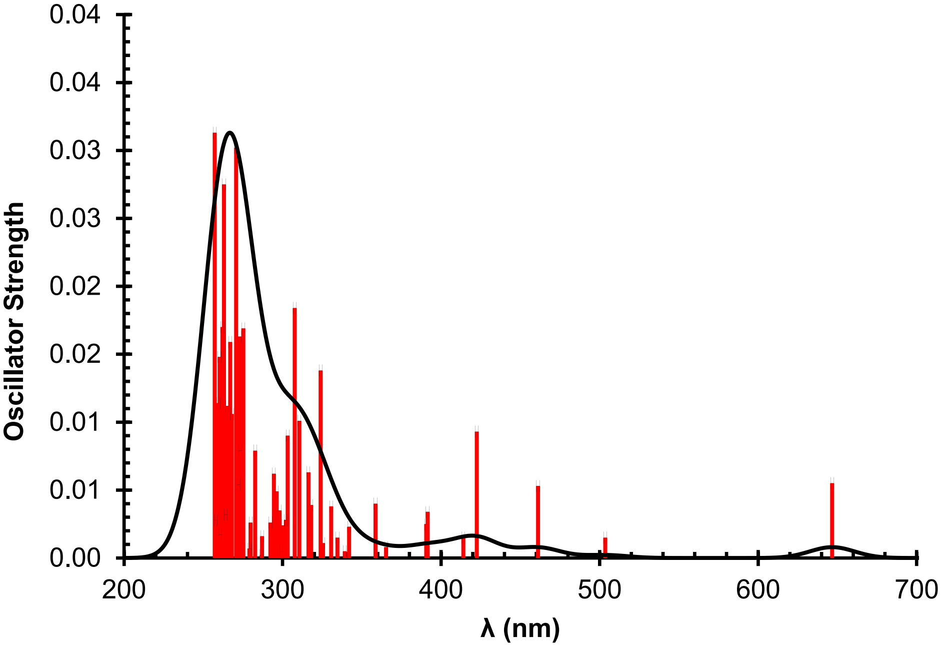

Figure S24. Simulated UV–vis spectrum of **T-Int1** at the SMD(diethyl ether)–B3LYP-D3/def2-TZVP//BS1 level of theory.

Table S9. Selected natural transition orbitals (NTOs) for transitions with λ > 450 nm in T-Int1. Hydrogen atoms are omitted for clarity.

Excitation (nm)	HOTO (Highest Occupied Transition Orbital)	LUTO (Lowest Unoccupied Transition Orbital)
λ = 646.7	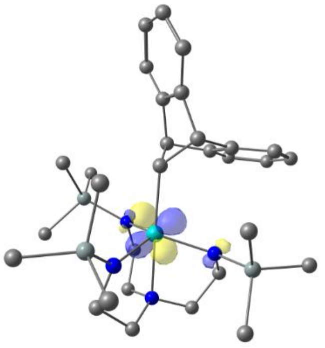	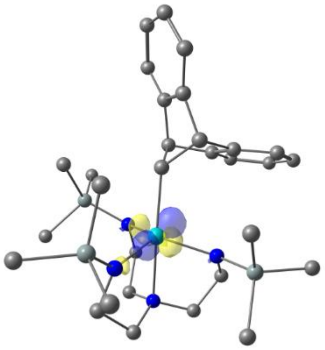
λ = 503.5	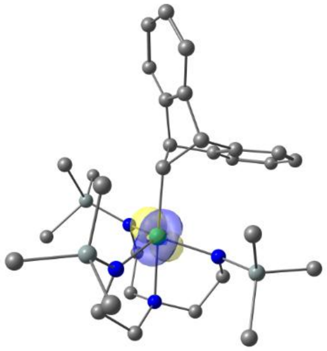	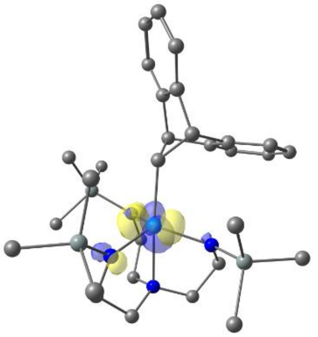
λ = 461.1	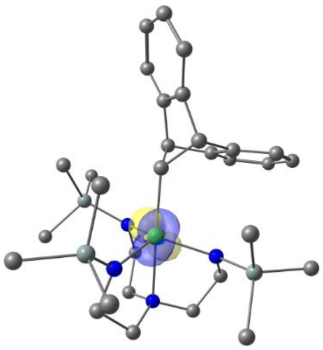	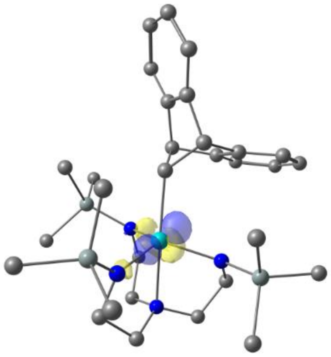

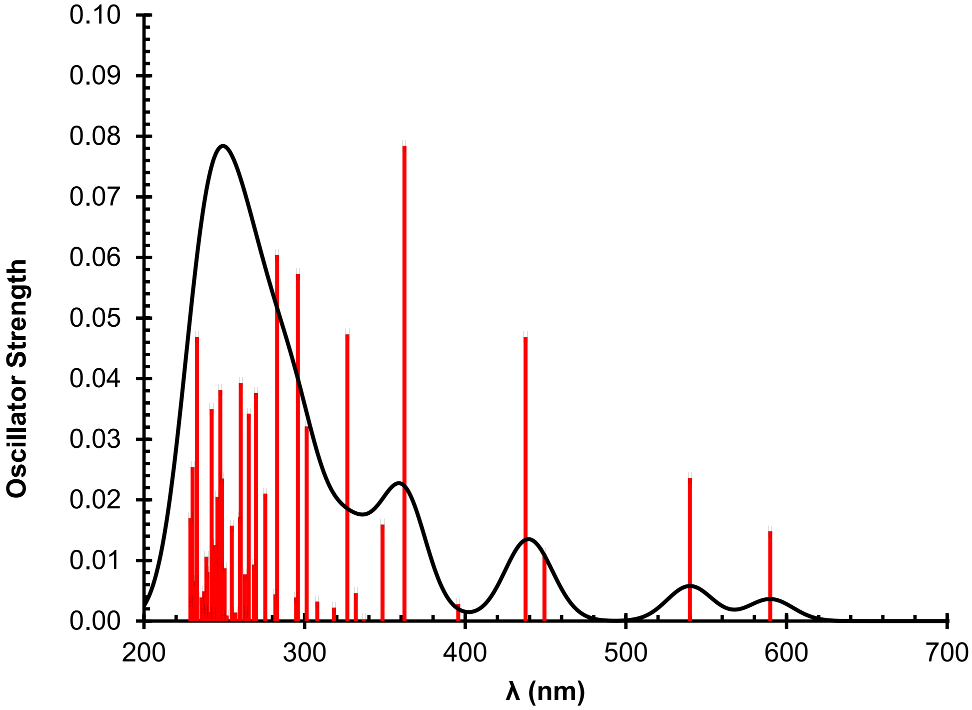

Figure S25. Simulated UV–vis spectrum of **S-Int1** at the SMD(diethyl ether)–B3LYP-D3/def2-TZVP//BS1 level of theory.

Table S10. Selected natural transition orbitals (NTOs) for transitions with λ > 450 nm in S-Int-1. Hydrogen atoms are omitted for clarity.

Excitation (nm)	HOTO (Highest Occupied Transition Orbital)	LUTO (Lowest Unoccupied Transition Orbital)
λ = 589.9	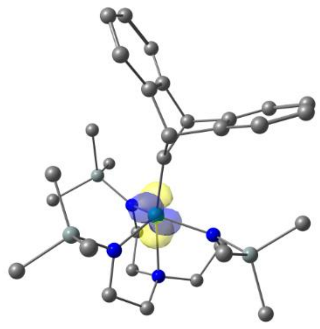	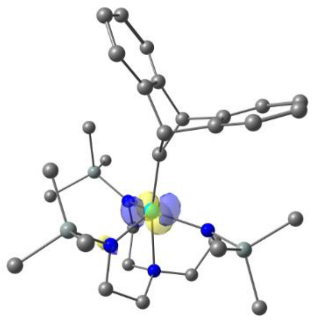

λ = 539.8	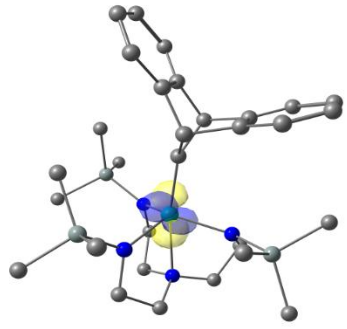	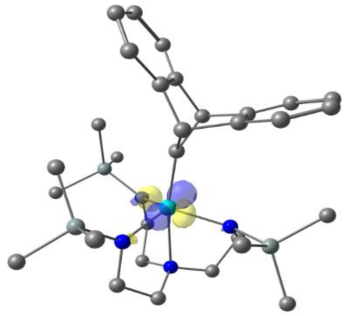

The UV-Vis spectra for **Int2** were also calculated in the open-shell singlet (Figure S26), triplet (Figure S27, Table S10), and closed-shell singlet (Figure S28) states. In all spin states this species would be expected to show absorbance in the visible region, and we cannot rule out that it could contribute to the observed spectra during the reaction.

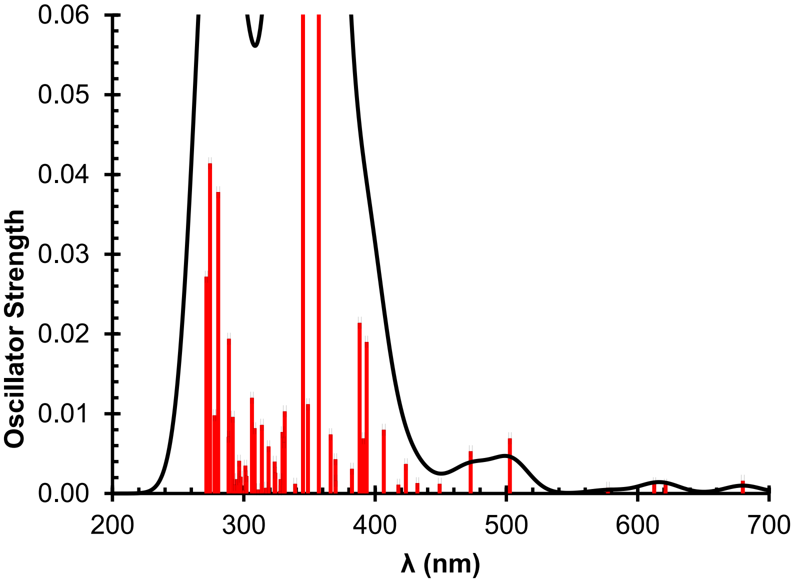

Figure S26. Simulated UV–vis spectrum of **OSS-Int2** at the SMD(diethyl ether)–UB3LYP-D3/def2-TZVP//BS1 level of theory.

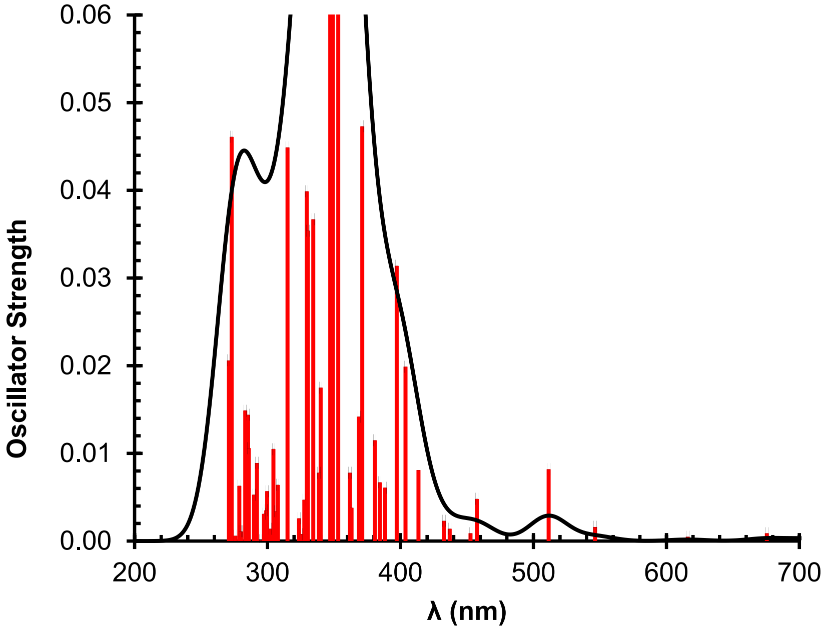

Figure S27. Simulated UV–vis spectrum of **T-Int2** at the SMD(diethyl ether)–UB3LYP-D3/def2-TZVP//BS1 level of theory.

Table S11. Selected natural transition orbitals (NTOs) for transitions with λ > 450 nm in **T-Int2**. Hydrogen atoms are omitted for clarity.

Excitation (nm)	HOTO (Highest Occupied Transition Orbital)	LUTO (Lowest Unoccupied Transition Orbital)
λ = 546.3	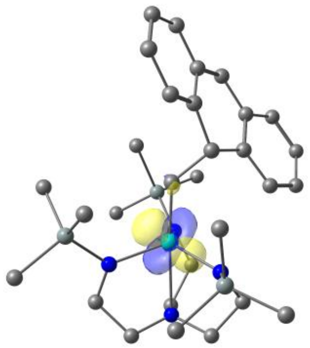	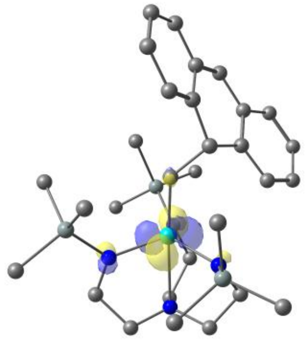

λ = 511.4	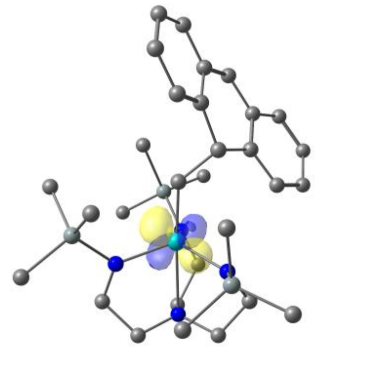	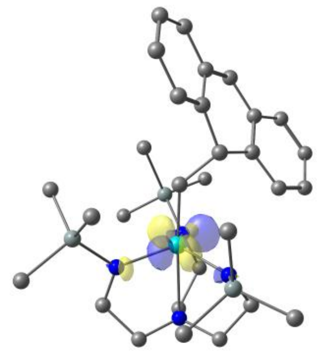
λ = 457.5	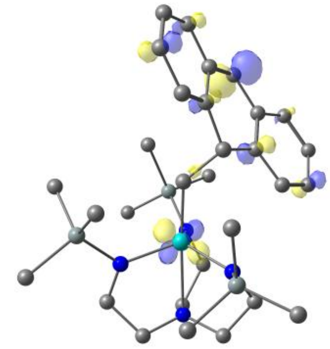	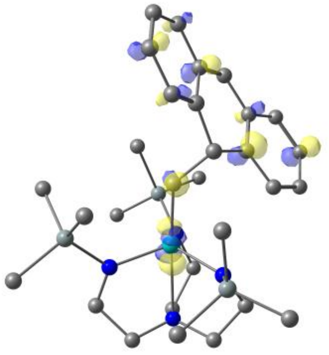

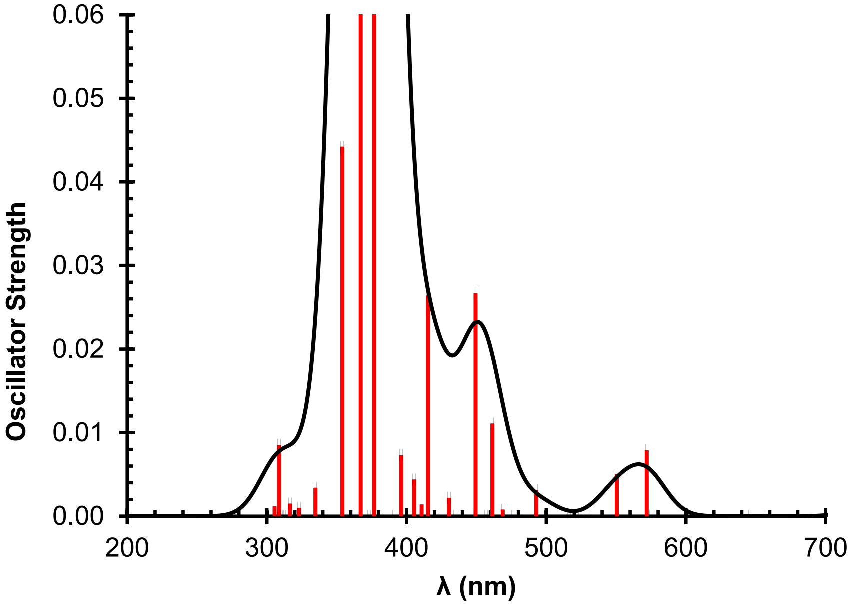

Figure S28. Simulated UV–vis spectrum of **S-Int2** (close-shell) at the SMD(diethyl ether)–B3LYP-D3/def2-TZVP//BS1 level of theory.

XYZ coordinates

88
S-Int1 el energy= -2326.60466489

C	3.462439	1.572321	0.657485
C	3.128311	0.512195	1.687340
H	2.879852	2.469411	0.877456
H	3.878526	-0.293475	1.656526
H	4.531480	1.838075	0.708267
H	3.239567	0.982082	2.676790
C	3.864653	0.030718	-1.214840
H	4.139727	-0.638684	-0.394899
H	4.790409	0.393471	-1.688975
C	2.987128	-0.733350	-2.180413
H	2.749942	-0.108313	-3.054232
H	3.560117	-1.586161	-2.569255
Mo	0.889565	0.177239	-0.279799
N	1.752853	-1.153871	-1.496220
N	1.775819	-0.033479	1.496890
N	3.066924	1.132142	-0.677752
C	2.909385	2.236395	-1.636685
H	3.213121	3.173069	-1.160441
H	3.559320	2.085679	-2.506529
C	1.434319	2.314919	-2.049142
H	1.230099	1.557500	-2.819117
H	1.223588	3.289947	-2.516011
N	0.621449	2.070312	-0.870437
Si	1.236466	-0.944301	2.911670
Si	0.058058	3.482339	0.011740
Si	1.575318	-2.915078	-1.593678
C	0.366575	0.194895	4.135541
H	-0.538455	0.640186	3.711083
H	0.080272	-0.354510	5.040687
H	1.024104	1.016406	4.443120
C	2.734377	-1.683218	3.799730
H	3.416052	-0.925977	4.200761
H	2.390314	-2.293145	4.643885
H	3.312927	-2.339569	3.138824
C	0.134111	-2.413588	2.520702
H	-0.070068	-2.938378	3.462787
H	-0.830794	-2.166748	2.074097
H	0.648890	-3.122549	1.864091
C	-0.400295	2.973790	1.756742
H	0.498941	2.730940	2.331933
H	-0.925736	3.785458	2.273439
H	-1.049902	2.096841	1.759938
C	-1.368651	4.278376	-0.911590
H	-1.795732	5.113822	-0.344162
H	-1.027252	4.675155	-1.875498
H	-2.164085	3.557325	-1.109834
C	1.392053	4.820036	0.133473
H	1.763844	5.128721	-0.850171
H	0.951098	5.709947	0.600919
H	2.250789	4.525508	0.745492
C	-0.106397	-3.572305	-1.090456
H	-0.366458	-3.448233	-0.037436
H	-0.905225	-3.130129	-1.691744
H	-0.106164	-4.650489	-1.296569
C	2.910907	-3.722042	-0.535064
H	2.814729	-3.407330	0.510489
H	2.840446	-4.815821	-0.565535
H	3.916950	-3.446901	-0.872401
C	1.789917	-3.449508	-3.392477
H	1.604926	-4.526817	-3.480722
H	1.062956	-2.937497	-4.033988
H	2.788064	-3.256153	-3.797671
C	-4.519489	2.766990	-0.030527
C	-3.785279	1.967402	0.855106
C	-3.702666	1.488634	-1.927709
C	-4.481129	2.529179	-1.402594
H	-5.124680	3.582062	0.357211
H	-3.827144	2.149742	1.926086
H	-3.682976	1.301470	-2.998500
H	-5.057207	3.160640	-2.073693
C	-3.017956	0.939160	0.333801
C	-2.221694	-0.175894	0.989562
C	-2.152606	-0.555020	-1.250082
C	-2.973229	0.703400	-1.050897
C	-1.108462	-0.385154	-0.089762
H	-0.628743	-1.384077	0.089612
C	-3.043760	-1.434582	0.739432
C	-3.007623	-1.665665	-0.649439
H	-1.914785	-0.008111	2.022790
H	-1.774065	-0.732988	-2.258369
C	-4.382419	-3.397333	1.026173
C	-3.729233	-2.288267	1.584840
C	-3.666744	-2.747990	-1.203008
C	-4.353887	-3.621815	-0.346607

H -4.916426 -4.088012 1.673513
H -3.756768 -2.113971 2.657808
H -3.646736 -2.930680 -2.274779
H -4.866434 -4.486037 -0.760940

88
S-Int2 el energy= -2326.58162749
C -3.895117 -0.615968 -0.965192
C -2.878875 -1.723196 -1.177347
H -3.943124 -0.019082 -1.880065
H -2.887514 -2.393488 -0.302064
H -4.896094 -1.033427 -0.775835
H -3.205947 -2.324543 -2.033677
C -3.781906 -0.239895 1.462249
H -4.074957 -1.290696 1.380719
H -4.631620 0.312457 1.888792
C -2.557794 -0.158507 2.367231
H -2.306957 0.883495 2.595403
H -2.779431 -0.629200 3.333646
Mo -0.944225 0.084043 0.038016
N -1.435602 -0.819660 1.691393
N -1.533617 -1.187325 -1.378949
N -3.451225 0.257735 0.121128
C -3.758983 1.672305 -0.067789
H -3.880122 1.857069 -1.138608
H -4.693920 1.959881 0.437276
C -2.591870 2.506720 0.420112
H -2.529926 2.492711 1.516241
H -2.786872 3.555211 0.157023
N -1.341949 2.023013 -0.179170
Si -0.559260 -1.758635 -2.735562
Si -0.561269 3.329448 -1.095428
Si -1.015225 -2.427019 2.333312
C 0.242759 -0.310260 -3.625686
H 1.117943 0.056279 -3.081499
H 0.589825 -0.632466 -4.615078
H -0.456744 0.519998 -3.769862
C -1.684634 -2.634021 -3.974284
H -2.512256 -1.999796 -4.312515
H -1.092274 -2.895864 -4.859455
H -2.107459 -3.566442 -3.583926
C 0.819425 -2.949431 -2.280455
H 1.373850 -3.209339 -3.191343
H 1.534726 -2.496691 -1.587365
H 0.452171 -3.879942 -1.836898
C 1.054461 2.894257 -1.921014
H 0.950713 2.128622 -2.692949
H 1.415502 3.805883 -2.414640
H 1.824641 2.580178 -1.213303
C -0.245520 4.769035 0.075613
H 0.202229 5.608507 -0.469873
H -1.156553 5.137045 0.558923
H 0.457761 4.464277 0.857879
C -1.760298 3.865602 -2.452522
H -2.728558 4.202448 -2.066564
H -1.333238 4.695111 -3.029049
H -1.945543 3.040022 -3.149762
C -0.160216 -3.435712 1.016479
H -0.891547 -3.777968 0.277498
H 0.605196 -2.871498 0.483464
H 0.309283 -4.321723 1.459802
C -2.588705 -3.341013 2.823702
H -3.253762 -3.492485 1.966544
H -2.309075 -4.333767 3.198148
H -3.158007 -2.846725 3.617636
C 0.032453 -2.218406 3.881147
H 0.150736 -3.181555 4.392358
H 1.033587 -1.831998 3.665944
H -0.445287 -1.528061 4.586195
C 2.726817 3.571575 2.182099
C 3.398731 3.055707 1.095880
C 1.663995 1.443388 2.577753
C 1.857827 2.764610 2.946051
H 2.870817 4.614022 2.456219
H 4.069225 3.686746 0.516596
H 0.968645 0.813784 3.130202
H 1.343177 3.178391 3.808437
C 3.251195 1.691083 0.717398
C 3.960819 1.106145 -0.350468
C 2.024190 -0.466031 0.955842
C 2.342689 0.894629 1.483736
C 0.936329 -0.003131 -0.076978
H 1.479366 0.497904 -0.897508
C 4.058332 -0.308407 -0.465743
C 3.189872 -1.130594 0.308053
H 4.626332 1.728586 -0.944268
H 1.565506 -1.098219 1.716128
C 5.196868 -2.324407 -1.228140

C 5.042784 -0.949278 -1.255491
C 3.388441 -2.507990 0.353085
C 4.387680 -3.116371 -0.403321
H 5.966203 -2.790680 -1.839132
H 5.700545 -0.338136 -1.869900
H 2.737970 -3.115688 0.977769
H 4.525113 -4.193067 -0.365681

89
S-Int3 el energy= -2334.02410599
C 3.823947 0.605962 -0.651415
C 3.588333 -0.419869 0.439960
H 3.800465 1.600074 -0.202412
H 3.775228 -1.432781 0.049169
H 4.815559 0.461760 -1.109538
H 4.351400 -0.250124 1.214904
C 2.881826 -0.571585 -2.580608
H 3.495081 -1.348710 -2.116651
H 3.381604 -0.255070 -3.507973
C 1.500030 -1.146505 -2.844761
H 0.881372 -0.415832 -3.383705
H 1.583070 -2.023766 -3.503299
Mo 0.753556 0.021251 -0.289064
N 0.909848 -1.475407 -1.549415
N 2.223794 -0.343321 0.987627
N 2.748096 0.544006 -1.641860
C 2.470057 1.821837 -2.316285
H 3.115605 2.594304 -1.890144
H 2.703789 1.759150 -3.386596
C 1.006992 2.213132 -2.102990
H 0.359283 1.639415 -2.779451
H 0.864086 3.271389 -2.366861
N 0.653432 1.931041 -0.718228
Si 2.182963 -0.714513 2.708118
Si 0.779285 3.278263 0.433855
Si 0.865495 -3.222306 -1.228024
C 0.456186 -0.952713 3.404835
H -0.033374 -1.851779 3.019631
H 0.543916 -1.066792 4.492824
H -0.201225 -0.101034 3.214652
C 3.005306 0.698197 3.652661
H 2.413391 1.617509 3.582870
H 3.110697 0.449406 4.715614
H 4.007988 0.916300 3.267362
C 3.163855 -2.292301 3.048493
H 3.119954 -2.545525 4.114633
H 2.763510 -3.145865 2.489591
H 4.221040 -2.186370 2.781560
C 0.024863 2.816553 2.086587
H 0.313929 1.808365 2.394986
H 0.391184 3.512934 2.850388
H -1.065903 2.879018 2.078795
C -0.121705 4.760778 -0.298312
H -1.093361 4.477746 -0.714743
H -0.292675 5.511209 0.482994
H 0.457735 5.246046 -1.091137
C 2.557980 3.794590 0.775224
H 3.116619 4.049958 -0.131184
H 2.551597 4.686830 1.414206
H 3.104221 3.012280 1.312399
C 0.275689 -3.629098 0.502886
H 0.772519 -3.011020 1.254650
H -0.806208 -3.516369 0.618460
H 0.523475 -4.675801 0.717096
C 2.585196 -3.958373 -1.447923
H 3.275156 -3.584176 -0.683341
H 2.538863 -5.049455 -1.345817
H 3.016252 -3.741493 -2.431232
C -0.300610 -4.022153 -2.472784
H -0.315398 -5.109624 -2.332061
H -1.326575 -3.658575 -2.345298
H -0.001360 -3.829454 -3.508870
C -3.566791 3.132997 -1.150395
C -3.123860 2.591963 0.073918
C -3.064108 1.042573 -2.298854
C -3.531342 2.372478 -2.321252
H -3.947302 4.149253 -1.181309
H -3.170455 3.185426 0.982207
H -3.073162 0.443229 -3.204977
H -3.884867 2.802533 -3.253108
C -2.638804 1.291623 0.089245
C -2.245001 0.357702 1.218177
C -2.242414 -0.890222 -0.673205
C -2.624304 0.513501 -1.092805
C -1.220579 -0.557083 0.477031
H -1.080580 -1.463709 1.076237
C -3.447075 -0.580366 1.316708
C -3.447039 -1.352356 0.137173

H -1.945746 0.836952 2.148067
H -1.912283 -1.551247 -1.473769
C -5.459325 -1.671296 2.018828
C -4.453589 -0.720631 2.258012
C -4.449916 -2.275414 -0.109720
C -5.457983 -2.435474 0.855117
H -6.244893 -1.816135 2.755231
H -4.460477 -0.127949 3.169295
H -4.453890 -2.874251 -1.017193
H -6.242478 -3.169562 0.693917
Li -4.825078 1.131230 -0.697944

89
S-Int4 el energy= -2334.01532943
C -3.919756 0.022502 0.733529
C -3.096037 1.249688 1.076962
H -3.836662 -0.684295 1.563231
H -3.231099 2.020197 0.301435
H -4.980264 0.293932 0.618854
H -3.511647 1.670049 2.002772
C -3.747943 0.011017 -1.729027
H -4.097819 1.025870 -1.521228
H -4.556677 -0.527093 -2.241491
C -2.497516 0.092150 -2.596708
H -2.177926 -0.912149 -2.900423
H -2.700123 0.643882 -3.522387
Mo -0.936619 -0.188045 -0.198370
N -1.454419 0.747293 -1.797890
N -1.661867 0.957761 1.224085
N -3.391916 -0.636446 -0.463703
C -3.553270 -2.094344 -0.480944
H -3.923193 -2.421974 0.493746
H -4.281880 -2.407672 -1.238870
C -2.195393 -2.732374 -0.728691
H -1.900304 -2.631539 -1.781495
H -2.246490 -3.812062 -0.536037
N -1.211210 -2.085004 0.143092
Si -0.936277 1.703158 2.663363
Si -0.564738 -3.146090 1.422114
Si -1.124422 2.457988 -2.239149
C 0.930080 1.606532 2.704103
H 1.382072 2.095298 1.836508
H 1.266672 2.153898 3.594174
H 1.331258 0.593389 2.774555
C -1.675251 0.835385 4.164601
H -1.469681 -0.240423 4.157916
H -1.260783 1.248919 5.091586
H -2.762865 0.964592 4.208551
C -1.408580 3.525294 2.762918
H -1.198175 3.896600 3.773342
H -0.823339 4.131538 2.064223
H -2.470223 3.706800 2.565292
C 0.565913 -2.233901 2.599726
H 0.151473 -1.273189 2.914388
H 0.678814 -2.848167 3.501425
H 1.569072 -2.071876 2.192681
C 0.371000 -4.537784 0.583191
H 1.182645 -4.136438 -0.034024
H 0.815038 -5.201612 1.334604
H -0.273124 -5.148325 -0.058504
C -2.030036 -3.811952 2.393473
H -2.754204 -4.337292 1.761777
H -1.682095 -4.523437 3.151769
H -2.554711 -3.003735 2.915595
C -0.338059 3.357564 -0.802689
H -1.112340 3.623530 -0.078040
H 0.423930 2.780505 -0.275787
H 0.118727 4.288583 -1.159258
C -2.743119 3.318708 -2.644995
H -3.417345 3.329755 -1.781999
H -2.525031 4.362823 -2.901527
H -3.275594 2.881362 -3.495377
C -0.037028 2.426787 -3.767903
H 0.070776 3.437864 -4.178195
H 0.965844 2.043925 -3.554176
H -0.476081 1.797892 -4.550781
C 3.063510 -3.252638 -2.481615
C 3.592258 -2.866157 -1.268250
C 2.023277 -1.101216 -2.762681
C 2.285673 -2.367805 -3.255234
H 3.257772 -4.256891 -2.849637
H 4.201235 -3.558296 -0.691436
H 1.384206 -0.415502 -3.316125
H 1.887152 -2.682365 -4.214733
C 3.359294 -1.562117 -0.740984
C 3.984654 -1.072739 0.440498
C 2.065701 0.585937 -0.854099
C 2.539990 -0.684142 -1.526495

C 0.942791 0.012546 -0.001694
H 1.369380 -0.632644 0.779401
C 3.991853 0.330452 0.725322
C 3.180560 1.208343 -0.059060
H 4.617014 -1.736817 1.022543
H 1.677436 1.290329 -1.592287
C 4.989133 2.304311 1.761158
C 4.885087 0.930675 1.653192
C 3.335205 2.595893 0.040221
C 4.235222 3.154757 0.936973
Li 4.820365 -0.003203 -1.285297
H 5.500831 0.286317 2.276411
H 2.707335 3.236852 -0.575166
H 5.678538 2.729859 2.485910
H 4.336102 4.232504 1.017266

64
S-TMS-TREN)Mo≡CH el energy= -1787.66609321
C 1.253552 -0.824623 2.810709
C 2.388448 -0.392174 1.902706
H 1.088150 -1.894590 2.657431
H 2.733990 0.617457 2.180322
H 1.510585 -0.662775 3.869780
H 3.235393 -1.062216 2.101849
C 0.004607 1.277965 2.801024
H 1.028761 1.649559 2.700502
H -0.306236 1.420527 3.847545
C -0.882047 2.082571 1.866923
H -1.939841 1.879192 2.092545
H -0.722124 3.144267 2.093482
Mo 0.062164 -0.025009 0.053870
N -0.583476 1.799398 0.458397
N 1.982082 -0.444248 0.500900
N 0.010960 -0.141363 2.438129
C -1.202623 -0.878313 2.818633
H -0.901235 -1.882640 3.128080
H -1.698996 -0.402328 3.675600
C -2.160819 -1.007711 1.635737
H -2.743322 -0.081331 1.524776
H -2.887551 -1.795448 1.875208
N -1.438393 -1.308781 0.404545
Si 3.239754 -0.460273 -0.728860
Si -2.055163 -2.446480 -0.773135
Si -1.175788 2.916052 -0.773563
C 2.950383 -1.794431 -2.023296
H 2.093938 -1.571768 -2.665244
H 3.835485 -1.889413 -2.664853
H 2.773321 -2.766003 -1.549002
C 4.899191 -0.850009 0.090053
H 4.906127 -1.835146 0.570811
H 5.686871 -0.855834 -0.673228
H 5.182400 -0.105103 0.842237
C 3.421665 1.215275 -1.569080
H 4.278371 1.225036 -2.254160
H 2.523367 1.468920 -2.139236
H 3.577481 2.006426 -0.825917
C -0.634031 -3.439237 -1.500691
H -0.057148 -3.929831 -0.708112
H -1.002911 -4.215757 -2.182044
H 0.051706 -2.794566 -2.058389
C -3.008629 -1.623889 -2.173654
H -3.442753 -2.378258 -2.841542
H -3.826750 -1.004130 -1.790124
H -2.352753 -0.983623 -2.771896
C -3.223345 -3.656996 0.086696
H -4.148927 -3.183142 0.432136
H -3.508656 -4.449382 -0.616000
H -2.748019 -4.141217 0.947850
C 0.148442 3.330431 -2.038675
H 1.072178 3.654018 -1.547041
H 0.385515 2.474454 -2.675940
H -0.194883 4.148718 -2.683833
C -1.680096 4.532300 0.064333
H -0.838284 5.019734 0.569682
H -2.053168 5.231915 -0.693474
H -2.481140 4.393092 0.798936
C -2.697914 2.219684 -1.629740
H -3.139027 2.950419 -2.318503
H -2.443789 1.320115 -2.197387
H -3.464206 1.943478 -0.895835
C 0.126855 -0.023824 -1.705008
H 0.183900 -0.079815 -2.795539

88
T-Int1 el energy= -2326.61915507
C 3.856171 -0.210406 -0.564487
C 3.400470 -1.192651 0.497547

H	4.043993	0.751127	-0.081866
H	3.362319	-2.213898	0.090469
H	4.792943	-0.547700	-1.035647
H	4.170638	-1.206610	1.282596
C	2.674725	-1.120044	-2.506063
H	3.025095	-2.032294	-2.015393
H	3.304025	-0.946776	-3.391741
C	1.211125	-1.305375	-2.870273
H	0.857599	-0.431416	-3.436752
H	1.119783	-2.156474	-3.561304
Mo	0.779833	-0.096377	-0.249364
N	0.404586	-1.491587	-1.661032
N	2.085625	-0.817447	1.036750
N	2.792360	-0.008324	-1.554454
C	2.830209	1.313462	-2.209467
H	3.618270	1.909482	-1.742571
H	3.083949	1.209743	-3.272359
C	1.486169	2.022697	-2.032513
H	0.755112	1.607162	-2.740846
H	1.596094	3.078906	-2.319150
N	0.989001	1.876743	-0.668202
Si	1.922723	-1.161451	2.760913
Si	1.345716	3.182326	0.438552
Si	-0.114537	-3.154472	-1.435202
C	0.170776	-0.992488	3.403286
H	-0.490613	-1.774941	3.019695
H	0.203160	-1.094953	4.495376
H	-0.283554	-0.024278	3.178270
C	3.027195	0.034940	3.709890
H	2.644560	1.058803	3.629427
H	3.064775	-0.224816	4.774668
H	4.056779	0.033254	3.334398
C	2.491356	-2.929470	3.081678
H	2.367083	-3.183339	4.141199
H	1.896788	-3.642061	2.498024
H	3.545308	-3.085914	2.828018
C	0.492799	2.932142	2.092948
H	0.587073	1.902251	2.450449
H	0.962478	3.582322	2.840935
H	-0.569236	3.185031	2.051643
C	0.821094	4.832724	-0.306741
H	-0.204102	4.782988	-0.686380
H	0.867338	5.622540	0.452993
H	1.472762	5.139989	-1.132390
C	3.193711	3.314916	0.825713
H	3.806805	3.452890	-0.072015
H	3.379983	4.175753	1.480599
H	3.550289	2.420354	1.349356
C	-0.888236	-3.518851	0.236159
H	-0.252434	-3.185882	1.062729
H	-1.882380	-3.082902	0.366025
H	-0.991535	-4.608143	0.320878
C	1.380730	-4.304693	-1.575095
H	2.083222	-4.129481	-0.751526
H	1.059346	-5.351916	-1.516757
H	1.927341	-4.185733	-2.517037
C	-1.339451	-3.644604	-2.785633
H	-1.574394	-4.714148	-2.723157
H	-2.281583	-3.092714	-2.694106
H	-0.940600	-3.454021	-3.788566
C	-2.772796	3.713153	-1.395728
C	-2.536472	3.165053	-0.125712
C	-2.753447	1.502559	-2.398406
C	-2.870726	2.895582	-2.517444
H	-2.880163	4.789479	-1.504020
H	-2.463925	3.812594	0.744543
H	-2.852962	0.862768	-3.272287
H	-3.050868	3.339361	-3.493206
C	-2.384497	1.795374	-0.017100
C	-2.138147	0.871238	1.163359
C	-2.373629	-0.459960	-0.651327
C	-2.511494	0.968152	-1.145984
C	-1.290248	-0.251454	0.465421
H	-1.303557	-1.131167	1.119973
C	-3.444673	0.109561	1.355362
C	-3.592138	-0.714290	0.223745
H	-1.740830	1.340544	2.062438
H	-2.171484	-1.201426	-1.421264
C	-5.487481	-0.764267	2.245633
C	-4.387159	0.100066	2.366417
C	-4.680880	-1.557036	0.095737
C	-5.631742	-1.580492	1.128968
H	-6.232436	-0.798517	3.036503
H	-4.279943	0.735395	3.242586
H	-4.798678	-2.196000	-0.776537
H	-6.488120	-2.245957	1.055934

88

T-Int2 el energy= -2326.60252769
C -3.916666 -0.875252 -0.719931
C -3.210746 -0.185271 -1.871306
H -4.342809 -0.103815 -0.073244
H -2.875898 -0.926696 -2.613381
H -4.739096 -1.510218 -1.087091
H -3.946622 0.448566 -2.379327
C -2.561345 -2.897575 -0.569117
H -2.727740 -2.800077 -1.646074
H -3.184163 -3.729135 -0.204942
C -1.084374 -3.173797 -0.349889
H -0.898914 -3.425919 0.705072
H -0.822848 -4.081302 -0.913491
Mo -0.905433 -0.271715 -0.044631
N -0.263190 -2.032433 -0.761011
N -2.072402 0.600117 -1.382608
N -2.945747 -1.635497 0.068340
C -3.272596 -1.757708 1.494647
H -4.105887 -1.085005 1.717817
H -3.598224 -2.778565 1.740443
C -2.069748 -1.348034 2.341116
H -1.307686 -2.139922 2.297449
H -2.373467 -1.294960 3.395269
N -1.493174 -0.085224 1.880819
Si -1.710612 2.110465 -2.236030
Si -2.014175 1.394671 2.666660
Si 0.971541 -2.445276 -1.935624
C -1.177720 3.486328 -1.074988
H -0.200759 3.286799 -0.629031
H -1.102547 4.423509 -1.640846
H -1.900468 3.640749 -0.267358
C -3.273897 2.711867 -3.106604
H -4.118695 2.817778 -2.416052
H -3.074532 3.703641 -3.530609
H -3.586155 2.064003 -3.932648
C -0.384151 1.874791 -3.551862
H -0.360327 2.738047 -4.228310
H 0.613685 1.775609 -3.112954
H -0.579260 0.981989 -4.156776
C -0.694428 2.728393 2.602921
H -0.310452 2.866131 1.589764
H -1.120861 3.682984 2.933844
H 0.144802 2.493607 3.266123
C -2.393198 1.054063 4.483669
H -1.547206 0.576075 4.990980
H -2.587517 2.005473 4.993894
H -3.277420 0.423967 4.628967
C -3.586466 2.077500 1.873342
H -4.437915 1.407337 2.041774
H -3.851926 3.057466 2.288576
H -3.460129 2.189760 0.791350
C 2.072846 -1.013735 -2.420331
H 1.511904 -0.215538 -2.913157
H 2.599793 -0.585479 -1.565162
H 2.825457 -1.380631 -3.130357
C 0.095179 -3.045658 -3.500818
H -0.501341 -2.237389 -3.940321
H 0.819298 -3.374304 -4.256412
H -0.577189 -3.889167 -3.305340
C 2.048860 -3.853883 -1.295999
H 2.795104 -4.140333 -2.047150
H 2.582381 -3.550300 -0.389550
H 1.464494 -4.749443 -1.056315
C 2.057061 -2.539301 2.675875
C 1.616633 -1.278461 2.276233
C 4.086780 -2.224225 1.421953
C 3.297107 -3.015360 2.238396
H 1.438445 -3.147106 3.330682
H 0.649804 -0.902420 2.603906
H 5.060191 -2.577644 1.089890
H 3.644520 -3.998757 2.543148
C 2.387661 -0.473856 1.441611
C 1.863599 0.872041 0.981404
C 4.504273 -0.095595 0.244070
C 3.659250 -0.938191 1.017669
C 0.728011 0.743393 -0.047033
H 0.939460 1.341487 -0.944419
C 2.940060 1.787601 0.448881
C 4.185255 1.269115 0.008881
H 1.416786 1.351564 1.863446
H 5.461700 -0.480186 -0.098347
C 3.608788 3.999824 -0.300175
C 2.683250 3.148043 0.300437
C 5.106819 2.141458 -0.615799
C 4.821541 3.486168 -0.772154
H 3.385142 5.057829 -0.403769
H 1.736862 3.547182 0.658546
H 6.055063 1.737089 -0.962180

H 5.542576 4.142814 -1.251253

89
T-Int3 el energy= -2334.03697580
C 3.861803 0.303639 -0.703284
C 3.619385 -0.680566 0.424785
H 3.914790 1.307582 -0.278561
H 3.731478 -1.715078 0.068831
H 4.814103 0.093231 -1.213535
H 4.409812 -0.526409 1.173133
C 2.760781 -0.879247 -2.542815
H 3.262300 -1.702374 -2.026363
H 3.329107 -0.650389 -3.455709
C 1.333753 -1.302852 -2.843262
H 0.831805 -0.522883 -3.434401
H 1.351440 -2.192148 -3.489825
Mo 0.840728 -0.063065 -0.249985
N 0.607515 -1.549244 -1.592267
N 2.280576 -0.493077 1.006698
N 2.730202 0.281332 -1.641100
C 2.525236 1.559603 -2.353723
H 3.234358 2.293212 -1.962893
H 2.736217 1.439950 -3.423721
C 1.096443 2.060293 -2.130680
H 0.409704 1.501408 -2.783455
H 1.025612 3.105755 -2.463183
N 0.685596 1.900613 -0.735331
Si 2.230113 -0.756213 2.757254
Si 0.930225 3.290341 0.303810
Si 0.359437 -3.263251 -1.276956
C 0.488983 -0.779640 3.450019
H -0.071230 -1.665965 3.138004
H 0.564679 -0.806072 4.544326
H -0.095000 0.105899 3.184037
C 3.187922 0.634823 3.588611
H 2.671203 1.592680 3.460550
H 3.287141 0.450235 4.664967
H 4.199161 0.742514 3.180717
C 3.046304 -2.408267 3.137820
H 3.017515 -2.611344 4.214844
H 2.525356 -3.228268 2.630033
H 4.096075 -2.434275 2.826596
C 0.132458 3.059704 1.990503
H 0.300508 2.060064 2.401536
H 0.578916 3.777281 2.689221
H -0.944460 3.247904 1.972599
C 0.210356 4.832057 -0.512055
H -0.814927 4.667752 -0.858865
H 0.196575 5.660837 0.206127
H 0.805452 5.161725 -1.370878
C 2.753171 3.652022 0.645800
H 3.334647 3.817343 -0.267810
H 2.841684 4.561477 1.253647
H 3.221192 2.835419 1.206815
C -0.258101 -3.630907 0.457456
H 0.370049 -3.154539 1.217356
H -1.296824 -3.337045 0.630716
H -0.192994 -4.715072 0.612830
C 1.998965 -4.182581 -1.453694
H 2.705518 -3.860369 -0.679491
H 1.840756 -5.260487 -1.326387
H 2.475023 -4.037671 -2.429438
C -0.857330 -3.982168 -2.526920
H -0.927269 -5.070549 -2.411790
H -1.864833 -3.571320 -2.394612
H -0.549082 -3.780350 -3.559065
C -3.287256 3.137650 -1.513720
C -2.862478 2.741414 -0.229444
C -2.940783 0.881399 -2.366275
C -3.318800 2.223994 -2.569428
H -3.602577 4.162807 -1.681521
H -2.859302 3.456372 0.587742
H -3.002041 0.163839 -3.179767
H -3.657058 2.545885 -3.549505
C -2.459284 1.427245 -0.039060
C -2.100167 0.631012 1.200434
C -2.223944 -0.861369 -0.496995
C -2.518039 0.495857 -1.101711
C -1.159380 -0.450846 0.574668
H -1.068971 -1.270807 1.294699
C -3.356382 -0.200677 1.451131
C -3.434440 -1.126118 0.391383
H -1.741143 1.205188 2.051055
H -1.965124 -1.639620 -1.209915
C -5.401623 -1.065807 2.344855
C -4.338836 -0.150956 2.425533
C -4.494527 -2.012447 0.296462
C -5.478166 -1.980881 1.298644

H -6.169776 -1.063311 3.113179
H -4.285176 0.560544 3.245778
H -4.558456 -2.730693 -0.517273
H -6.305089 -2.684504 1.260455
Li -4.660556 1.307151 -0.792067

89
T-Int4 el energy= -2334.00370605
C 4.020234 0.469160 -0.095306
C 3.592256 -0.705777 0.759513
H 3.987639 1.368806 0.524388
H 3.762144 -1.654354 0.228378
H 5.053597 0.335305 -0.452483
H 4.252772 -0.727148 1.638096
C 3.256349 -0.297652 -2.295305
H 3.710205 -1.207172 -1.890748
H 3.930478 0.102608 -3.067297
C 1.897003 -0.662031 -2.870240
H 1.453220 0.215908 -3.359116
H 2.029543 -1.407601 -3.664627
Mo 0.914139 0.026616 -0.218281
N 1.018116 -1.158847 -1.803631
N 2.178099 -0.601962 1.151552
N 3.077644 0.660307 -1.199912
C 2.964494 2.055707 -1.648534
H 3.506873 2.688883 -0.941845
H 3.429723 2.189560 -2.634885
C 1.499359 2.485963 -1.666906
H 1.009831 2.084847 -2.567277
H 1.455102 3.578938 -1.782137
N 0.787881 2.043813 -0.475835
Si 1.910997 -1.181021 2.806918
Si 0.583052 3.210599 0.815408
Si 0.616801 -2.880108 -1.874646
C 0.117606 -1.419053 3.296324
H -0.410731 -2.110082 2.633668
H 0.116417 -1.854643 4.303726
H -0.445374 -0.482645 3.348903
C 2.680342 0.072378 3.983545
H 2.120916 1.014555 3.966169
H 2.670041 -0.302046 5.014155
H 3.721327 0.297378 3.725851
C 2.775269 -2.842539 3.015192
H 2.662765 -3.194410 4.047662
H 2.336280 -3.602383 2.358979
H 3.847947 -2.792029 2.801480
C -0.569371 2.530569 2.132369
H -0.134155 1.670076 2.650304
H -0.757642 3.306021 2.885128
H -1.530789 2.223022 1.711077
C -0.115631 4.833305 0.159045
H -1.094080 4.685075 -0.308089
H -0.239685 5.544997 0.984275
H 0.543040 5.306240 -0.577845
C 2.235394 3.599797 1.645452
H 2.951169 4.043676 0.943534
H 2.105548 4.309431 2.472175
H 2.685828 2.688356 2.055310
C 0.225485 -3.568867 -0.176566
H 1.130418 -3.549902 0.440103
H -0.538824 -2.991509 0.346628
H -0.105544 -4.611935 -0.256513
C 2.103096 -3.849730 -2.514928
H 2.986794 -3.686440 -1.887791
H 1.870736 -4.921208 -2.475877
H 2.371928 -3.616324 -3.550219
C -0.798821 -3.180020 -3.087273
H -0.964110 -4.254749 -3.231040
H -1.746537 -2.739727 -2.759791
H -0.558138 -2.751976 -4.067862
C -3.261180 3.587103 -1.501912
C -3.823089 2.949890 -0.411062
C -2.033598 1.576261 -2.059858
C -2.371743 2.897835 -2.337216
H -3.513041 4.621921 -1.713447
H -4.524673 3.471748 0.234433
H -1.323727 1.049930 -2.693245
H -1.934333 3.398751 -3.195538
C -3.507245 1.598222 -0.124608
C -4.184007 0.875673 0.904330
C -2.121411 -0.482415 -0.587716
C -2.579890 0.910415 -0.963679
C -0.899396 -0.357455 0.326352
H -1.183669 -0.359050 1.383279
C -4.117892 -0.549665 0.968892
C -3.214098 -1.253888 0.114689
H -4.867535 1.403727 1.563339
H -1.820919 -1.003505 -1.503299

C -5.047502 -2.669482 1.715800
C -5.018130 -1.288644 1.775678
C -3.293911 -2.643999 0.046146
C -4.199575 -3.351534 0.834924
Li -4.784871 0.026036 -1.212822
H -5.694049 -0.749122 2.434065
H -2.622557 -3.180196 -0.617861
H -5.739573 -3.223737 2.342551
H -4.238280 -4.434735 0.771843

64
T-TMS-TREN)Mo≡CH el energy= -1787.58600820
C 1.147583 0.685872 2.693480
C 0.966125 1.991135 1.947619
H 2.103475 0.248653 2.391878
H 0.151422 2.585094 2.389805
H 1.164631 0.840436 3.783897
H 1.882905 2.584628 2.085230
C -1.236565 0.097455 2.760737
H -1.340273 1.183150 2.680073
H -1.385371 -0.185740 3.813712
C -2.265838 -0.548390 1.857033
H -2.261249 -1.639979 2.026806
H -3.259347 -0.196518 2.165254
Mo -0.056014 -0.021072 -0.071051
N -1.997319 -0.217710 0.462457
N 0.700673 1.719340 0.537420
N 0.105407 -0.268021 2.296186
C 0.477950 -1.660921 2.598776
H 1.470184 -1.665678 3.059210
H -0.222902 -2.099114 3.318868
C 0.519365 -2.472731 1.299758
H -0.500639 -2.799508 1.046397
H 1.107150 -3.386705 1.464705
N 1.076589 -1.651130 0.245260
Si 1.002143 3.087342 -0.534438
Si 2.393407 -2.157561 -0.778299
Si -3.250025 -0.536008 -0.721205
C 0.309296 2.790008 -2.251380
H -0.764686 2.578237 -2.230440
H 0.459063 3.695424 -2.853176
H 0.811919 1.963431 -2.762435
C 2.857168 3.387066 -0.663198
H 3.352476 2.544468 -1.158216
H 3.069018 4.291576 -1.245935
H 3.319607 3.514954 0.322209
C 0.179606 4.620667 0.189861
H 0.315400 5.481110 -0.476039
H -0.897968 4.462654 0.315280
H 0.595145 4.892779 1.166315
C 3.318594 -0.633407 -1.376264
H 3.585130 0.009705 -0.529944
H 4.241787 -0.909007 -1.900546
H 2.700128 -0.046784 -2.063327
C 1.781841 -3.156104 -2.254024
H 2.602813 -3.438571 -2.924014
H 1.285965 -4.076127 -1.922968
H 1.053025 -2.575679 -2.830768
C 3.579074 -3.232484 0.222571
H 3.125073 -4.180045 0.533139
H 4.462588 -3.479526 -0.378378
H 3.926374 -2.713560 1.123589
C -3.341108 0.805992 -2.038369
H -3.381024 1.797202 -1.571390
H -2.486133 0.783535 -2.719059
H -4.252745 0.683117 -2.636672
C -4.928755 -0.522613 0.154901
H -5.124541 0.434010 0.653874
H -5.727595 -0.674514 -0.581348
H -5.023105 -1.318442 0.902269
C -3.061855 -2.237182 -1.511922
H -3.849662 -2.426080 -2.251403
H -2.090710 -2.325770 -2.009448
H -3.124918 -3.023548 -0.749827
C -0.258632 -0.394242 -1.924288
H -0.237724 -0.239159 -3.008852

88
S-TS1 el energy= -2326.55339534
C -2.818622 2.181882 0.573417
C -1.617523 2.580977 1.398947
H -3.335514 1.365587 1.084876
H -1.182789 3.516348 1.012302
H -3.521235 3.022641 0.462282
H -1.984157 2.815415 2.410190
C -1.912085 2.693203 -1.661201
H -1.435452 3.498108 -1.097083

H	-2.750762	3.121113	-2.230276
C	-0.862850	2.026794	-2.528499
H	-1.314030	1.190863	-3.082757
H	-0.510755	2.727958	-3.299836
Mo	-0.492646	0.320196	-0.157530
N	0.214757	1.555216	-1.657822
N	-0.598465	1.520252	1.415922
N	-2.385421	1.661913	-0.725385
C	-3.415416	0.812932	-1.363146
H	-4.307320	0.796475	-0.730251
H	-3.706698	1.232492	-2.332238
C	-2.851662	-0.591275	-1.499533
H	-2.190832	-0.647458	-2.377239
H	-3.665260	-1.305736	-1.698829
N	-2.112936	-0.907995	-0.292162
Si	0.414584	1.701840	2.862007
Si	-2.921952	-1.957642	0.843274
Si	1.605893	2.623979	-1.770542
C	2.047882	0.774470	2.858438
H	2.702080	1.254727	3.597264
H	1.945654	-0.274477	3.149652
H	2.562182	0.800838	1.896856
C	-0.555904	1.148263	4.383447
H	-0.666927	0.059279	4.411877
H	-0.030326	1.451601	5.297420
H	-1.559110	1.587979	4.420557
C	0.804775	3.538377	3.098957
H	1.293837	3.981156	2.224189
H	-0.091678	4.129847	3.313296
H	1.485272	3.659154	3.950481
C	-2.266934	-1.795526	2.596523
H	-2.162400	-0.741351	2.869913
H	-2.988745	-2.253612	3.284507
H	-1.303723	-2.287619	2.756064
C	-2.865184	-3.773994	0.333102
H	-1.871066	-4.202847	0.490885
H	-3.578509	-4.361276	0.924513
H	-3.119530	-3.904740	-0.724640
C	-4.754143	-1.483277	0.940908
H	-5.260844	-1.513576	-0.029900
H	-5.280014	-2.184758	1.600276
H	-4.889428	-0.479691	1.360644
C	2.738269	2.606383	-0.267876
H	3.390368	3.486827	-0.326713
H	2.151897	2.695376	0.649709
H	3.384939	1.731970	-0.173293
C	1.063397	4.435713	-1.880594
H	0.609451	4.763946	-0.938028
H	1.952268	5.057685	-2.044440
H	0.360939	4.657131	-2.689979
C	2.622274	2.225569	-3.310873
H	1.999373	2.220835	-4.213117
H	3.420531	2.962165	-3.463437
H	3.093199	1.239029	-3.229219
C	-0.116864	-3.585371	-3.075352
C	0.031656	-3.742318	-1.693082
C	0.965393	-1.418221	-2.975252
C	0.338710	-2.429038	-3.707181
H	-0.594741	-4.368212	-3.658152
H	-0.343859	-4.635397	-1.201620
H	1.318844	-0.515241	-3.463613
H	0.212210	-2.314403	-4.780665
C	0.644587	-2.738864	-0.962068
C	0.821634	-2.598459	0.527000
C	1.750820	-0.650051	-0.625689
C	1.132852	-1.583414	-1.600334
C	0.329485	-1.176634	0.879369
H	0.356653	-1.052791	1.969955
C	2.298640	-2.506000	0.818888
C	2.791542	-1.351471	0.190603
H	0.301218	-3.360653	1.115699
H	2.095718	0.289261	-1.037310
C	4.461076	-2.945647	1.752237
C	3.116129	-3.305081	1.599567
C	4.133465	-1.015691	0.318861
C	4.962790	-1.814846	1.114708
H	5.115356	-3.553899	2.370891
H	2.720476	-4.189748	2.092634
H	4.540842	-0.144831	-0.188157
H	6.009504	-1.548174	1.234158

88

S-TS2 el energy= -2326.57689589

C	-4.049384	-0.313955	-0.634501
C	-3.187943	-1.543463	-0.855382
H	-4.156486	0.203923	-1.591356
H	-3.167999	-2.144251	0.068135
H	-5.054425	-0.588070	-0.279122

H -3.664125 -2.166742 -1.622034
C -3.602402 0.252174 1.711265
H -4.022230 -0.756980 1.755626
H -4.335797 0.938852 2.159292
C -2.294723 0.251816 2.496140
H -1.916797 1.275141 2.615056
H -2.492484 -0.103975 3.516399
Mo -0.956244 0.134517 0.006953
N -1.296415 -0.585241 1.818951
N -1.831163 -1.164803 -1.233440
N -3.369982 0.589971 0.296629
C -3.549334 2.006576 -0.011046
H -3.726090 2.097602 -1.086318
H -4.416619 2.432625 0.516928
C -2.279939 2.767117 0.313304
H -2.148911 2.856159 1.401116
H -2.410716 3.798048 -0.045195
N -1.113014 2.121951 -0.301009
Si -1.078949 -1.820375 -2.681932
Si -0.177716 3.293013 -1.231696
Si -0.994218 -2.169771 2.536343
C -0.321858 -0.439937 -3.713154
H 0.624757 -0.098484 -3.286027
H -0.114402 -0.793863 -4.730490
H -0.995846 0.420802 -3.785637
C -2.400136 -2.653629 -3.744382
H -3.219029 -1.971825 -4.002011
H -1.941847 -2.981360 -4.685449
H -2.833041 -3.541660 -3.270516
C 0.264779 -3.087708 -2.336885
H 0.695694 -3.433566 -3.285061
H 1.078979 -2.657483 -1.746217
H -0.118074 -3.964296 -1.803945
C 1.379986 2.648294 -2.038591
H 1.179782 1.879274 -2.789131
H 1.853156 3.494272 -2.554066
H 2.099480 2.257674 -1.316524
C 0.309352 4.734671 -0.117208
H 0.810147 5.518032 -0.699078
H -0.549616 5.194313 0.383643
H 1.007705 4.392195 0.653570
C -1.286160 3.947387 -2.616857
H -2.214345 4.398219 -2.248491
H -0.759841 4.715038 -3.197188
H -1.557610 3.139291 -3.306305
C -0.429836 -3.359437 1.204087
H -1.299115 -3.739468 0.656546
H 0.223645 -2.876563 0.476137
H 0.095967 -4.218179 1.637224
C -2.593837 -2.856667 3.265245
H -3.378401 -2.950878 2.506481
H -2.395288 -3.863103 3.654724
H -2.990414 -2.263182 4.095611
C 0.243115 -2.061489 3.953906
H 0.303050 -3.023925 4.477054
H 1.254073 -1.799919 3.625800
H -0.071215 -1.309576 4.687536
C 3.033122 3.325933 2.103443
C 3.746451 2.736085 1.090113
C 1.710141 1.323727 2.387358
C 2.006122 2.613664 2.768750
H 3.256981 4.347279 2.400265
H 4.536543 3.283237 0.581263
H 0.905224 0.773806 2.868696
H 1.454907 3.087412 3.576182
C 3.486360 1.391784 0.694542
C 4.244086 0.739357 -0.291677
C 2.068265 -0.633302 0.884762
C 2.432471 0.687526 1.358287
C 0.813476 -0.121847 -0.393874
H 1.519810 0.089210 -1.202108
C 4.118954 -0.647867 -0.504233
C 3.096636 -1.377243 0.181674
H 5.026293 1.287870 -0.811174
H 1.465499 -1.217552 1.571113
C 4.951302 -2.738022 -1.434533
C 5.022962 -1.369026 -1.331731
C 3.063247 -2.778676 0.071358
C 3.974889 -3.457336 -0.713554
H 5.657732 -3.272000 -2.064834
H 5.788909 -0.815477 -1.870051
H 2.302580 -3.329181 0.617065
H 3.937511 -4.540776 -0.780835

89
S-TS3 el energy= -2333.99328896
C 3.804496 0.217179 -0.652505
C 3.427672 -0.776721 0.430874

H	3.865403	1.209131	-0.204113
H	3.473052	-1.802687	0.033938
H	4.793789	-0.032424	-1.066879
H	4.201646	-0.713457	1.208841
C	2.798362	-0.879891	-2.605776
H	3.323352	-1.707892	-2.121621
H	3.327426	-0.640465	-3.538831
C	1.364116	-1.297329	-2.854930
H	0.833773	-0.522203	-3.425422
H	1.338775	-2.200617	-3.478462
Mo	0.719572	-0.004326	-0.314509
N	0.713860	-1.519865	-1.557903
N	2.090689	-0.527882	0.997327
N	2.776547	0.263856	-1.692700
C	2.640051	1.556592	-2.372502
H	3.383156	2.256177	-1.980978
H	2.824470	1.452660	-3.447930
C	1.231407	2.104556	-2.121083
H	0.509777	1.584073	-2.766130
H	1.173032	3.166760	-2.390845
N	0.895618	1.883870	-0.719573
Si	1.968368	-1.090201	2.674319
Si	1.195903	3.217377	0.432280
Si	0.293286	-3.233767	-1.293790
C	0.228309	-1.013861	3.363878
H	-0.487880	-1.632291	2.815831
H	0.262266	-1.385435	4.396014
H	-0.151596	0.010711	3.395861
C	3.071508	-0.002518	3.746870
H	2.715272	1.033175	3.766470
H	3.076804	-0.371639	4.779427
H	4.110540	0.008330	3.399922
C	2.601870	-2.862391	2.773712
H	2.549083	-3.224551	3.807329
H	2.009537	-3.544823	2.154702
H	3.646604	-2.941560	2.453885
C	0.515157	2.767890	2.112424
H	0.946768	1.827139	2.462798
H	0.789796	3.549397	2.831053
H	-0.572031	2.676960	2.111033
C	0.381394	4.752406	-0.274354
H	-0.637483	4.545956	-0.613700
H	0.330338	5.530597	0.496497
H	0.950503	5.165327	-1.114563

C	3.031456	3.580323	0.650461
H	3.555299	3.764389	-0.293088
H	3.126345	4.492702	1.253360
H	3.549012	2.781955	1.191982
C	-0.555707	-3.559898	0.345159
H	-0.033286	-3.128710	1.202801
H	-1.597546	-3.221770	0.357302
H	-0.570836	-4.647346	0.492159
C	1.888318	-4.228109	-1.353686
H	2.549548	-3.950952	-0.524778
H	1.673288	-5.299707	-1.265213
H	2.437649	-4.081140	-2.289862
C	-0.869902	-3.772763	-2.668215
H	-0.427914	-3.683771	-3.665957
H	-1.152626	-4.822521	-2.525364
H	-1.788681	-3.176330	-2.650224
C	-3.174933	3.977340	-0.178749
C	-2.874639	3.010412	0.782916
C	-3.002131	2.305470	-1.936472
C	-3.210264	3.622413	-1.531989
H	-3.351664	5.005839	0.120223
H	-2.805702	3.283454	1.833169
H	-3.079555	2.035794	-2.987349
H	-3.423868	4.381222	-2.280023
C	-2.617820	1.702004	0.385762
C	-2.198518	0.522492	1.224103
C	-2.659790	-0.126370	-1.189566
C	-2.729477	1.310220	-0.975736
C	-1.008367	-0.136653	0.517246
H	-1.044889	-1.234814	0.616146
C	-3.295613	-0.507198	1.115000
C	-3.402855	-0.937752	-0.239109
H	-1.958569	0.790721	2.257632
H	-2.603968	-0.483460	-2.216732
C	-4.859350	-2.193494	1.826102
C	-4.030569	-1.112012	2.129382
C	-4.233159	-2.042702	-0.520751
C	-4.939402	-2.659775	0.508983
H	-5.420387	-2.686994	2.613699
H	-3.921776	-0.769739	3.155905
H	-4.325443	-2.403748	-1.542453
H	-5.573956	-3.511893	0.280045
Li	-4.763726	0.775136	-0.360735

88
T-TS1 el energy= -2326.56629511
C 3.597835 -1.309017 -0.380015
C 2.783182 -2.008128 0.694124
H 4.093080 -0.448084 0.074430
H 2.351173 -2.937895 0.294699
H 4.373338 -1.980922 -0.780773
H 3.470097 -2.305249 1.495198
C 2.289223 -1.865609 -2.367827
H 2.398693 -2.832772 -1.869206
H 2.931044 -1.871622 -3.261805
C 0.823191 -1.680730 -2.722341
H 0.693095 -0.767360 -3.320668
H 0.513082 -2.502522 -3.384798
Mo 0.669395 -0.224052 -0.204282
N 0.005457 -1.624390 -1.509194
N 1.716336 -1.130753 1.198271
N 2.706124 -0.815421 -1.431964
C 3.147130 0.419241 -2.099695
H 4.007416 0.824315 -1.558040
H 3.473089 0.213118 -3.128587
C 2.015316 1.443080 -2.070348
H 1.222628 1.141012 -2.768916
H 2.379369 2.405795 -2.450679
N 1.462955 1.566607 -0.720986
Si 1.435907 -1.145307 2.947734
Si 2.185013 2.791632 0.308724
Si -0.963272 -3.064834 -1.270702
C 0.830775 0.501189 3.610143
H -0.112199 0.817075 3.156955
H 0.664770 0.401139 4.690328
H 1.573812 1.290376 3.461811
C 3.073604 -1.501359 3.817967
H 3.880211 -0.849096 3.463642
H 2.946092 -1.308585 4.890241
H 3.404726 -2.540276 3.715158
C 0.213736 -2.476109 3.474147
H 0.220806 -2.570965 4.567197
H -0.810163 -2.233614 3.172041
H 0.471052 -3.455790 3.056078
C 0.999697 3.536342 1.561289
H 0.524617 2.790399 2.200041
H 1.567764 4.211334 2.213750
H 0.217411 4.128098 1.077649
C 2.803823 4.223243 -0.755244
H 2.012691 4.614743 -1.405072
H 3.124358 5.040256 -0.097181
H 3.661545 3.959883 -1.383510
C 3.667735 2.124629 1.273840
H 4.496245 1.862723 0.605498
H 4.042740 2.871810 1.984173
H 3.399350 1.226211 1.838948
C -1.773555 -3.151712 0.415397
H -1.001124 -3.171314 1.188638
H -2.454724 -2.328814 0.638411
H -2.335244 -4.091313 0.489513
C 0.154785 -4.589845 -1.371764
H 0.875318 -4.604606 -0.545659
H -0.447963 -5.503793 -1.298783
H 0.715953 -4.647586 -2.311143
C -2.231247 -3.280948 -2.657775
H -2.792493 -4.212388 -2.513600
H -2.956785 -2.464223 -2.724238
H -1.732755 -3.352192 -3.631853
C -1.524136 3.379275 -2.673100
C -1.499459 3.253942 -1.278065
C -2.399024 1.129746 -2.878781
C -1.954306 2.317102 -3.465744
H -1.187507 4.302154 -3.137809
H -1.126003 4.069718 -0.665643
H -2.760185 0.311126 -3.496545
H -1.957276 2.416244 -4.548200
C -1.908083 2.068473 -0.695227
C -1.820333 1.627016 0.746615
C -2.868191 -0.129325 -0.709659
C -2.397521 1.011946 -1.488255
C -1.030950 0.292357 0.735069
H -1.342764 -0.343724 1.577152
C -3.199254 1.246141 1.226751
C -3.704401 0.187243 0.441503
H -1.350876 2.377518 1.388065
H -3.022059 -1.074273 -1.217908
C -5.082435 1.057032 2.707644
C -3.882932 1.686296 2.345264
C -4.886549 -0.450987 0.820046
C -5.570865 -0.008522 1.955046
H -5.622069 1.389904 3.590126
H -3.480199 2.493495 2.953114

H -5.277130 -1.275100 0.227705
H -6.494550 -0.499360 2.250384

88
T-TS2 el energy= -2326.53769578
C 4.159767 -1.166630 0.162082
C 4.041322 0.342310 0.039503
H 4.336213 -1.575300 -0.836349
H 3.980208 0.796503 1.039621
H 5.000254 -1.463439 0.806578
H 4.950363 0.733657 -0.435639
C 2.806983 -1.669864 2.128277
H 3.576684 -0.981722 2.490451
H 3.019455 -2.663676 2.548805
C 1.453758 -1.142379 2.588392
H 0.678450 -1.899760 2.407901
H 1.484449 -1.004771 3.680099
Mo 1.142640 -0.157889 -0.097326
N 1.130556 0.099580 1.897635
N 2.840552 0.646916 -0.729487
N 2.881875 -1.702243 0.658602
C 2.521744 -2.994055 0.064176
H 2.931940 -3.012728 -0.949547
H 2.960394 -3.832992 0.627053
C 1.015569 -3.135883 -0.051213
H 0.567200 -3.315310 0.937912
H 0.824557 -4.053473 -0.628122
N 0.418863 -1.959521 -0.684143
Si 2.904124 1.646527 -2.174887
Si -0.744711 -2.356135 -1.936501
Si 1.114257 1.559484 2.855609
C 1.628366 1.026630 -3.406907
H 0.607659 1.156641 -3.033939
H 1.712000 1.581791 -4.349312
H 1.776012 -0.036470 -3.627406
C 4.613533 1.517128 -2.958136
H 4.895724 0.474800 -3.145865
H 4.611279 2.036820 -3.923925
H 5.397318 1.976517 -2.346083
C 2.549845 3.451755 -1.782246
H 2.607067 4.071038 -2.685718
H 1.545535 3.563256 -1.359236
H 3.267053 3.852931 -1.056994
C -1.797271 -0.918574 -2.514909
H -1.226287 -0.179833 -3.084161
H -2.587777 -1.308598 -3.169603
H -2.272517 -0.405526 -1.675253
C -1.908630 -3.713341 -1.332917
H -2.629014 -3.970179 -2.119234
H -1.381896 -4.634535 -1.060175
H -2.475932 -3.380081 -0.458716
C 0.190011 -3.043561 -3.431009
H 0.835037 -3.886415 -3.154721
H -0.500867 -3.399155 -4.205416
H 0.826246 -2.272235 -3.880798
C 0.957151 3.080371 1.772059
H 1.792131 3.138065 1.066819
H 0.032920 3.073378 1.187047
H 0.968157 3.985725 2.391717
C 2.729440 1.698517 3.828132
H 3.587020 1.808810 3.154406
H 2.709053 2.576710 4.485258
H 2.910561 0.823491 4.462952
C -0.287065 1.524581 4.121530
H -0.208828 2.378127 4.806204
H -1.274202 1.569265 3.649477
H -0.254793 0.614129 4.731689
C -3.778843 -2.929211 1.786030
C -4.518194 -2.174923 0.914087
C -2.154951 -1.149997 2.028207
C -2.577291 -2.414874 2.342690
H -4.102919 -3.932931 2.046887
H -5.430352 -2.571121 0.474671
H -1.233060 -0.744216 2.433694
H -1.994376 -3.032422 3.020610
C -4.112865 -0.853671 0.565100
C -4.856672 -0.054221 -0.311432
C -2.526114 0.995670 0.865357
C -2.913616 -0.325800 1.153549
C -0.346310 0.947274 -0.517235
H -0.722189 1.807808 -1.085551
C -4.481642 1.264871 -0.593236
C -3.310780 1.814759 0.033926
H -5.756115 -0.460248 -0.769015
H -1.686209 1.425607 1.400619
C -4.871600 3.392659 -1.703655
C -5.239442 2.094947 -1.470141
C -2.969813 3.171763 -0.222704

C -3.725885 3.940855 -1.067978
H -5.458473 4.013032 -2.375356
H -6.119764 1.674157 -1.949900
H -2.091478 3.587016 0.265944
H -3.452108 4.975181 -1.256280

89
T-TS3 el energy= -2333.97947261
C -3.798575 0.832511 -0.308281
C -3.178434 1.222120 1.021007
H -4.180867 -0.186933 -0.220727
H -2.868333 2.276287 1.001335
H -4.639467 1.496901 -0.558612
H -3.949316 1.137375 1.794771
C -2.477143 2.192288 -1.854622
H -2.803280 2.918035 -1.104790
H -3.033623 2.390877 -2.781894
C -0.977969 2.354496 -2.044209
H -0.628827 1.722621 -2.872432
H -0.762745 3.386203 -2.357152
Mo -0.739733 0.178806 -0.141833
N -0.280228 2.014665 -0.801121
N -2.019714 0.365587 1.323486
N -2.765408 0.842868 -1.348968
C -2.945744 -0.151946 -2.416966
H -3.725222 -0.856270 -2.111912
H -3.276943 0.325230 -3.349221
C -1.639215 -0.912768 -2.617430
H -0.894811 -0.255954 -3.088137
H -1.793846 -1.731310 -3.330570
N -1.126101 -1.401765 -1.335428
Si -1.900582 -0.252977 2.991629
Si -1.660685 -3.022796 -0.869814
Si 0.317551 3.427957 0.063866
C -1.084840 -1.938885 3.102711
H -0.050880 -1.938656 2.749574
H -1.073058 -2.235737 4.159125
H -1.642833 -2.701964 2.553510
C -3.646957 -0.456571 3.671407
H -4.290167 -1.029901 2.994096
H -3.588029 -1.013611 4.614347
H -4.142829 0.494789 3.890542
C -0.967331 0.925873 4.118713
H -1.022951 0.562842 5.152463
H 0.090463 1.001924 3.848786
H -1.396187 1.933727 4.099708
C -0.406317 -3.975501 0.155503
H -0.062067 -3.440861 1.042420
H -0.885614 -4.900589 0.499498
H 0.463123 -4.267349 -0.443527
C -1.948689 -4.055233 -2.421991
H -1.071227 -4.069819 -3.078794
H -2.146364 -5.090393 -2.117407
H -2.810453 -3.724195 -3.011179
C -3.289474 -2.961023 0.079504
H -4.116773 -2.676667 -0.581067
H -3.535032 -3.939311 0.509812
H -3.248182 -2.231716 0.894206
C 0.832327 3.009709 1.814625
H -0.051987 2.934707 2.453058
H 1.380889 2.067879 1.879282
H 1.465757 3.808511 2.219300
C -1.051080 4.724409 0.186243
H -1.899324 4.358287 0.775049
H -0.661341 5.615884 0.693663
H -1.427576 5.048636 -0.790093
C 1.718760 4.251280 -0.897439
H 1.934157 5.239784 -0.473846
H 2.648539 3.675939 -0.883781
H 1.439303 4.400664 -1.946974
C 2.322679 -0.887051 -3.726338
C 2.078069 -1.585490 -2.538196
C 2.938728 1.092444 -2.460953
C 2.738930 0.444467 -3.681473
H 2.171532 -1.377769 -4.683143
H 1.714240 -2.608754 -2.564286
H 3.282799 2.122172 -2.436756
H 2.919323 0.983263 -4.607436
C 2.248567 -0.939817 -1.319880
C 1.942336 -1.450177 0.066338
C 2.929430 0.892005 0.093333
C 2.698590 0.402357 -1.264308
C 0.966010 -0.444468 0.704204
H 1.111903 -0.370179 1.789694
C 3.220134 -1.374224 0.864853
C 3.671598 -0.031193 0.944999
H 1.528216 -2.459596 0.041876
H 3.056000 1.957226 0.253894

C 5.006189 -2.077331 2.318843
C 3.891440 -2.391680 1.532336
C 4.782683 0.271342 1.745603
C 5.435603 -0.754359 2.430638
H 5.526022 -2.863928 2.857341
H 3.529766 -3.415150 1.470473
H 5.136024 1.296385 1.821406
H 6.295069 -0.518461 3.051681
Li 4.582056 -1.002165 -1.006048

88
MECP el energy= -2326.57792518
C 3.937562 -0.542428 0.975429
C 2.920057 -1.651525 1.193098
H 3.989976 0.057265 1.888564
H 2.945613 -2.339059 0.329617
H 4.940411 -0.961675 0.788824
H 3.247257 -2.239544 2.059707
C 3.836074 -0.160337 -1.455246
H 4.131777 -1.211247 -1.376385
H 4.692113 0.394440 -1.872407
C 2.620730 -0.076943 -2.378393
H 2.381393 0.968329 -2.607805
H 2.865433 -0.537091 -3.345446
Mo 0.965346 0.121452 -0.055874
N 1.479086 -0.738344 -1.738665
N 1.567572 -1.126415 1.376345
N 3.497217 0.329056 -0.114327
C 3.771473 1.749019 0.081279
H 3.884448 1.932873 1.153378
H 4.705512 2.060105 -0.415853
C 2.590014 2.566384 -0.408886
H 2.541922 2.562534 -1.507117
H 2.769788 3.616328 -0.138297
N 1.341383 2.062281 0.173450
Si 0.621400 -1.737300 2.779202
Si 0.465650 3.410674 1.007619
Si 1.060989 -2.363075 -2.417662
C -0.300024 -0.324896 3.632339
H -1.153796 0.024541 3.046490
H -0.688574 -0.683000 4.592601
H 0.364494 0.521667 3.832976
C 1.824443 -2.456974 4.067018
H 2.617239 -1.751630 4.339577
H 1.255680 -2.677531 4.977762
H 2.291983 -3.393938 3.746551
C -0.627577 -3.091021 2.357028
H -1.118647 -3.416205 3.282044
H -1.411722 -2.737744 1.681143
H -0.151871 -3.967447 1.906462
C -1.104719 2.954002 1.927838
H -0.930548 2.229505 2.726312
H -1.473238 3.875039 2.396421
H -1.894091 2.579809 1.273941
C 0.071250 4.738438 -0.284874
H -0.387768 5.608292 0.198611
H 0.966924 5.085917 -0.810042
H -0.634851 4.348210 -1.024508
C 1.651239 4.135249 2.308988
H 2.587262 4.518799 1.890921
H 1.158744 4.970394 2.820019
H 1.899147 3.384295 3.066887
C 0.297871 -3.444103 -1.080879
H 1.073341 -3.781491 -0.386506
H -0.461345 -2.923522 -0.496166
H -0.160407 -4.332376 -1.530085
C 2.647346 -3.233873 -3.000092
H 3.370622 -3.354434 -2.187325
H 2.377752 -4.238981 -3.345115
H 3.146332 -2.732019 -3.834746
C -0.050058 -2.181443 -3.941184
H -0.153716 -3.151674 -4.440352
H -1.053332 -1.821014 -3.699321
H 0.390276 -1.483415 -4.661391
C -3.209346 3.492971 -2.023001
C -3.702997 2.902434 -0.881354
C -1.975909 1.489819 -2.568641
C -2.346746 2.784257 -2.888770
H -3.492050 4.514313 -2.266793
H -4.376500 3.452426 -0.227599
H -1.285878 0.943702 -3.209438
H -1.973919 3.255392 -3.793869
C -3.380696 1.553950 -0.552545
C -3.929538 0.876179 0.554614
C -2.020651 -0.478856 -0.972978
C -2.478952 0.860201 -1.420916
C -0.893174 0.023159 0.068043
H -1.492309 0.482327 0.882819

C -3.937701 -0.545190 0.590820
C -3.085799 -1.260557 -0.297856
H -4.578101 1.421314 1.237687
H -1.536183 -1.038546 -1.772547
C -4.927825 -2.662463 1.276375
C -4.829424 -1.289201 1.402519
C -3.228185 -2.642817 -0.435562
C -4.146791 -3.349944 0.333181
H -5.631082 -3.211105 1.898163
H -5.465478 -0.757682 2.106869
H -2.605570 -3.173135 -1.152341
H -4.250048 -4.424465 0.213610

88
OSS-Int2 el energy= -2326.60320621
C -4.000056 0.603069 -1.003253
C -3.655778 -0.828831 -0.638197
H -3.867978 0.719393 -2.082393
H -3.864017 -1.001265 0.429060
H -5.046058 0.842658 -0.757403
H -4.317825 -1.499406 -1.200125
C -3.456702 1.795276 1.056549
H -4.196412 1.049544 1.363488
H -3.931426 2.785551 1.123533
C -2.267738 1.685357 2.003890
H -1.586222 2.534994 1.852654
H -2.636047 1.787388 3.034333
Mo -0.976048 0.200173 -0.102544
N -1.567530 0.419894 1.803113
N -2.243840 -1.087900 -0.929327
N -3.059502 1.501955 -0.326829
C -2.679408 2.681092 -1.108919
H -2.803562 2.432812 -2.166748
H -3.327303 3.542417 -0.882000
C -1.214815 3.021901 -0.885891
H -1.067937 3.444823 0.118512
H -0.957618 3.836427 -1.579312
N -0.356247 1.849983 -1.082061
Si -1.848673 -2.526200 -1.871213
Si 0.837813 2.079417 -2.348338
Si -1.488024 -0.684602 3.160412
C -0.326822 -2.244597 -2.931044
H 0.584316 -2.137272 -2.336980
H -0.188304 -3.097803 -3.606593
H -0.442340 -1.346611 -3.547596
C -3.279187 -2.923980 -3.036521
H -3.580582 -2.050715 -3.626484
H -2.955301 -3.699305 -3.741520
H -4.165390 -3.308774 -2.520572
C -1.563441 -4.031751 -0.775750
H -1.386358 -4.924040 -1.388912
H -0.694129 -3.897394 -0.123899
H -2.432240 -4.233735 -0.138895
C 2.010446 0.637558 -2.564352
H 1.523945 -0.235867 -3.003597
H 2.821566 0.943332 -3.237632
H 2.460188 0.333587 -1.618007
C 1.863559 3.624338 -2.003412
H 2.560749 3.813022 -2.829026
H 1.247307 4.522342 -1.883714
H 2.451716 3.498352 -1.088602
C -0.074995 2.342405 -3.984294
H -0.743412 3.210892 -3.952664
H 0.631112 2.504366 -4.808034
H -0.681849 1.463648 -4.233305
C -1.084254 -2.432782 2.616925
H -1.786879 -2.768300 1.848345
H -0.073605 -2.524941 2.211712
H -1.168022 -3.111624 3.474878
C -3.186404 -0.750700 3.987627
H -3.949024 -1.111292 3.287480
H -3.160655 -1.448325 4.833672
H -3.518891 0.216993 4.378500
C -0.241521 -0.120030 4.460379
H -0.319668 -0.737845 5.363506
H 0.789677 -0.190304 4.097773
H -0.414961 0.920933 4.758201
C 3.249178 3.386615 1.555903
C 4.067679 2.473312 0.916476
C 1.578382 1.664435 1.843447
C 1.995293 2.981382 2.024430
H 3.579656 4.412837 1.691120
H 5.045765 2.774606 0.548949
H 0.597617 1.353543 2.192591
H 1.345136 3.689812 2.530468
C 3.662602 1.129803 0.735021
C 4.528158 0.191784 0.112017
C 1.899929 -0.697792 1.030449

C	2.386050	0.725992	1.205413
C	0.612295	-0.838134	0.214197
H	0.519084	-1.875627	-0.137312
C	4.205283	-1.188304	0.030929
C	2.961160	-1.655669	0.526052
H	5.488832	0.531667	-0.266373
H	1.619970	-1.052278	2.039626
C	4.841000	-3.473999	-0.520970
C	5.124805	-2.121067	-0.504092
C	2.708678	-3.026116	0.520915
C	3.630274	-3.935363	0.007086
H	5.560016	-4.176424	-0.933403
H	6.070222	-1.752604	-0.895154
H	1.767457	-3.392624	0.926082
H	3.406002	-4.998177	0.016249

References:

[1] R. R. Schrock, S. W. Seidel, N. C. Mösch-Zanetti, D. A. Dobbs, K.-Y. Shih and W. M. Davis, Synthesis and Decomposition of Alkyl Complexes of Tungsten(IV) That Contain a $[(Me_3SiNCH_2CH_2)_3N]^{3-}$ Ligand, *Organometallics* 1997, **16**, 5195–5208.

[2] W. T. Ford, R. Radue and J. A. Walker, The Mechanism of Addition of Benzyne to Cyclopentadienyl- and Indenyl-Magnesium Bromide, *J. Chem. Soc. Chem. Commun.* 1970, 966–967.

[3] U. Tilstam and H. Weinmann, Activation of Mg Metal for Safe Formation of Grignard Reagents on Plant Scale, *Org. Process Res. Dev.* 2002, **6**, 906–910.

[4] M. N. Paddon-Row, E. Cotsaris and H. K. Patney, The Synthesis of Rigid Norbornylogs for the Purpose of Studying Orbital Interactions through Bonds, *Tetrahedron* 1986, **42**, 1779–1788.

[5] Bruker, SAINT. Bruker AXS Inc., Madison, Wisconsin (USA), **2012**.

[6] Bruker, SADABS. Bruker AXS Inc., Madison, Wisconsin (USA), **2001**.

[7] G. M. Sheldrick, SHELXT – Integrated Space-Group and Crystal-Structure Determination, *Acta Crystallogr. Sect. Found. Adv.* 2015, **71**, 3–8.

[8] G. M. Sheldrick, Crystal Structure Refinement with SHELXL, *Acta Crystallogr. Sect. C Struct. Chem.* 2015, **71**, 3–8.

[9] O. V. Dolomanov, L. J. Bourhis, R. J. Gildea, J. A. K. Howard and H. Puschmann, OLEX2: A Complete Structure Solution, Refinement and Analysis Program, *J. Appl. Crystallogr.* 2009, **42**, 339–341.

[10] J. B. Greco, J. C. Peters, T. A. Baker, W. M. Davis, C. C. Cummins and G. Wu, Atomic Carbon as a Terminal Ligand: Studies of a Carbidomolybdenum Anion Featuring Solid-State ^{13}C NMR Data and Proton-Transfer Self-Exchange Kinetics, *J. Am. Chem. Soc.* 2001, **123**, 5003–5013.

[11] J. A. Buss, G. A. Bailey, J. Oppenheim, D. G. VanderVelde, W. A. I. Goddard and T. Agapie, CO Coupling Chemistry of a Terminal Mo Carbide: Sequential Addition of Proton, Hydride, and CO Releases Ethenone, *J. Am. Chem. Soc.* 2019, **141**, 15664–15674.

[12] G. A. Bailey, J. A. Buss, P. H. Oyala and T. Agapie, Terminal, Open-Shell Mo Carbide and Carbyne Complexes: Spin Delocalization and Ligand Noninnocence, *J. Am. Chem. Soc.* 2021, **143**, 13091–13102.

[13] M. J. Frisch, G. W. Trucks, H. B. Schlegel, G. E. Scuseria, M. A. Robb, J. R. Cheeseman, G. Scalmani, V. Barone, G. A. Petersson, H. Nakatsuji, X. Li, M. Caricato, A. V. Marenich, J. Bloino, B. G. Janesko, R. Gomperts, B. Mennucci, H. P. Hratchian and J. V., Gaussian 16, Revision C.01, Gaussian, Inc., Wallingford CT (USA), **2016**.

[14] C. Adamo and V. Barone, Toward Reliable Density Functional Methods without Adjustable Parameters: The PBE0 Model, *J. Chem. Phys.* 1999, **110**, 6158–6170.

[15] J. P. Perdew, M. Ernzerhof and K. Burke, Rationale for Mixing Exact Exchange with Density Functional Approximations, *J. Chem. Phys.* 1996, **105**, 9982–9985.

[16] S. Grimme, Semiempirical GGA-Type Density Functional Constructed with a Long-Range Dispersion Correction, *J. Comput. Chem.* 2006, **27**, 1787–1799.
[17] G. A. Petersson and M. A. Al-Laham, A Complete Basis Set Model Chemistry. II. Open-Shell Systems and the Total Energies of the First-Row Atoms, *J. Chem. Phys.* 1991, **94**, 6081–6090.
[18] P. C. Hariharan and J. A. Pople, The Influence of Polarization Functions on Molecular Orbital Hydrogenation Energies, *Theor. Chim. Acta* 1973, **28**, 213–222.
[19] W. J. Hehre, R. Ditchfield and J. A. Pople, Self—Consistent Molecular Orbital Methods. XII. Further Extensions of Gaussian—Type Basis Sets for Use in Molecular Orbital Studies of Organic Molecules, *J. Chem. Phys.* 1972, **56**, 2257–2261.
[20] M. Couty and M. B. Hall, Basis Sets for Transition Metals: Optimized Outer p Functions, *J. Comput. Chem.* 1996, **17**, 1359–1370.
[21] P. J. Hay and W. R. Wadt, Ab Initio Effective Core Potentials for Molecular Calculations. Potentials for K to Au including the outermost core orbitals, *J. Chem. Phys.* 1985, **82**, 299–310.
[22] P. J. Hay and W. R. Wadt, Ab initio effective core potentials for molecular calculations. Potentials for the transition metal atoms Sc to Hg, *J. Chem. Phys.* 1985, **82**, 270–283.
[23] A. V. Marenich, C. J. Cramer and D. G. Truhlar, Universal Solvation Model Based on Solute Electron Density and on a Continuum Model of the Solvent Defined by the Bulk Dielectric Constant and Atomic Surface Tensions, *J. Phys. Chem. B* 2009, **113**, 6378–6396.
[24] J. D. Snyder, L.-H. Hamill, K. E. Faleumu, A. R. Schultz and D. H. Ess, MECPro Version 1.0.6: Minimum Energy Crossing Program, Brigham Young University, Provo, Utah (US), 2020.
[25] A. D. Becke, Density-Functional Thermochemistry. III. The Role of Exact Exchange, *J. Chem. Phys.* 1993, **98**, 5648–5652.
[26] C. Lee, W. Yang and R. G. Parr, Development of the Colle-Salvetti Correlation-Energy Formula into a Functional of the Electron Density, *Phys. Rev. B* 1988, **37**, 785–789.
[27] F. Weigend and R. Ahlrichs, Balanced Basis Sets of Split Valence, Triple Zeta Valence and Quadruple Zeta Valence Quality for H to Rn: Design and Assessment of Accuracy, *Phys. Chem. Chem. Phys.* 2005, **7**, 3297–3305.
[28] R. L. Jarek, T. D. Miles, M. L. Trester, S. C. Denson and S. K. Shin, Solvation of Li^+ by Acetone, THF, and Diethyl Ether in the Gas Phase and the Ion−Molecule Association Mechanism, *J. Phys. Chem. A* 2000, **104**, 2230–2237.
[29] M. Brym, C. Jones, P. C. Junk and M. Kloth, X-ray Structural Characterization of Diethyl Ether Solvated Lithium Iodide Derived from a Metathesis Reaction, *Z. Für Anorg. Allg. Chem.* 2006, **632**, 1402–1404.
[30] P. S. Kulyabin, G. P. Goryunov, A. N. Iashin, D. Y. Mladentsev, D. V. Uborsky, C. Ehm, J. A. M. Canich, J. R. Hagadorn and A. Z. Voskoboynikov, Reversible C–C Bond Formation in Group 4 Metal Complexes: Nitrile Extrusion via β-aryl Elimination, *Chem. Sci.* 2024, **15**, 15825–15834.
[31] M. D. R. Lutz, S. Roediger, M. A. Rivero-Crespo and B. Morandi, "Mechanistic Investigation of the Rhodium-Catalyzed Transfer Hydroarylation Reaction Involving Reversible C–C Bond Activation" *J. Am. Chem. Soc.* 2023, **145**, 26657–26666.
[32] J. Ma, J. Duan and Z.-X. Yu, Ni(0)-Catalyzed Rearrangement of Vinylcyclobutanones (VCBOs) to Synthesize Six-Membered Non-Conjugated Enones, *Angewandte Chemie International Edition* 2025, **64**, e202417407.
[33] S. Kim, P.-P. Chen, K. N. Houk and R. R. Knowles, Reversible Homolysis of a Carbon–Carbon σ-Bond Enabled by Complexation-Induced Bond-Weakening, *J. Am. Chem. Soc.* 2022, **144**, 15488–15496.
